# Siderophore systems at play: system interactions as drivers of diversification in pathogenic *Vibrio*

**DOI:** 10.1128/mbio.01412-25

**Published:** 2025-08-18

**Authors:** Marta A. Lages, Lucía Ageitos, Larissa Buedenbender, Jaime Rodríguez, Manuel L. Lemos, Carlos Jiménez, Miguel Balado

**Affiliations:** 1 Department of Microbiology and Parasitology, Aquatic One Health Research Center (iARCUS), Universidade de Santiago de Compostela 16780, Santiago de Compostela, Spain; 2 CICA – Centro Interdisciplinar de Química e Bioloxía and Departamento de Química, Universidade da Coruña 16737, A Coruña, Spain; Weill Cornell Medicine, New York, New York, USA

**Keywords:** *Vibrio *virulence, siderophore, siderophore metabolome, genomic island, diversification, virulence evolution

## Abstract

The *irp*-HPI genomic island encodes the salicylate-type siderophore piscibactin, a key virulence factor in several pathogenic *Vibrio* species. To advance our understanding of the siderophore metabolome (siderome) in *Vibrio anguillarum*, we combined high-resolution metabolomics with genetic and phenotypic analyses of biosynthetic mutants. LC-MS/MS and Feature-Based Molecular Networking (FBMN) revealed the presence of known siderophores, vanchrobactin and piscibactin/photoxenobactin-like siderophores, alongside two non-reported catecholate analogs of the formed siderophores, 2-hydroxypiscibactin (2-OH-Pcb) and 2-hydroxyphotoxenobactin E (2-OH-PxbE). Functional characterization of mutants defective in piscibactin or vanchrobactin production demonstrated biosynthetic and uptake-level interactions between these systems. We show that the salicylate-activating enzyme Irp5 is a promiscuous aryl acid adenylation enzyme capable of activating 2,3-dihydroxybenzoate (DHBA) and driving the synthesis of catecholate-piscibactin/photoxenobactin and fluorinated analogs through precursor-directed biosynthesis. Our data support photoxenobactin E as the terminal product of the piscibactin/photoxenobactin pathway in *V. anguillarum*. Comparative genomics revealed frequent co-occurrence of *irp*-HPI with other siderophore systems, especially catecholate-type systems such as vanchrobactin. These findings provide new insight into the molecular basis of virulence in *Vibrionaceae* and highlight the ecological and evolutionary significance of siderophore system interplay, which may enhance metabolic diversity, promote environmental adaptability, and preserve virulence under mutational pressure.

**IMPORTANCE:**

Iron acquisition is critical for bacterial survival and virulence, especially under the iron-limited conditions encountered in host environments. This study uncovers unexpected siderophore diversification within the piscibactin pathway, including two novel catecholate analogs and fluorinated derivatives produced via precursor-directed biosynthesis. We identify Irp5 as a versatile salicylate-activating enzyme that drives this metabolic flexibility. Moreover, the piscibactin and vanchrobactin systems interact at both biosynthetic and uptake levels, forming a resilient and adaptable iron acquisition network that supports virulence. These findings advance our understanding of siderophore-mediated iron acquisition and illustrate how enzymatic promiscuity and system interplay can be leveraged for synthetic biology and antimicrobial development. Given the widespread distribution of *irp*-HPI in *Vibrionaceae*, including human pathogens, our study provides a foundation for the rational design of antimicrobial strategies.

## INTRODUCTION

**Fig 1:**
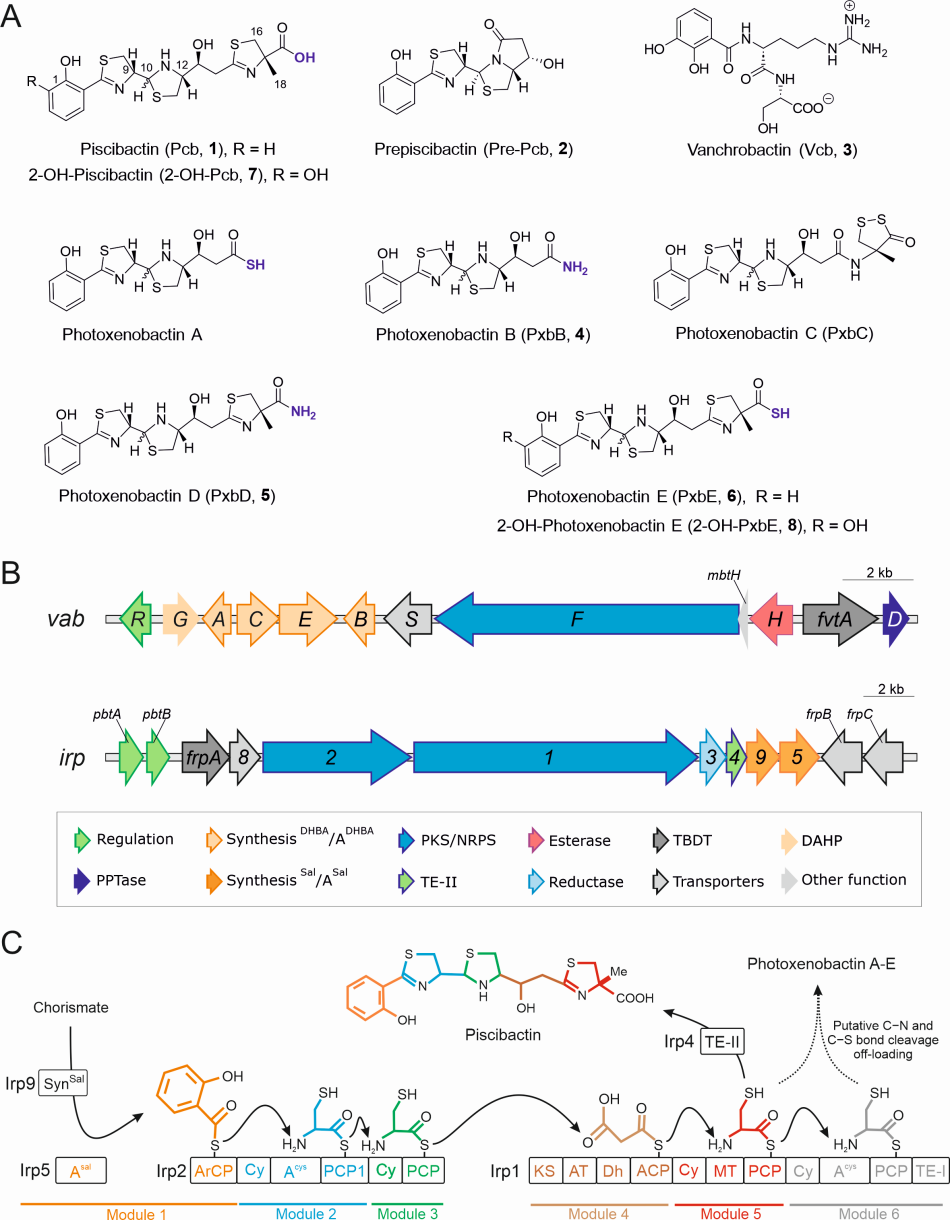
Chemical structures of siderophores, *V. anguillarum* gene clusters, and the previously described biosynthetic pathway of piscibactin (19, 20). (**A**) Chemical structures of the compounds isolated from *V. anguillarum* RV22. (**B**) Piscibactin (*irp*) and vanchrobactin (*vab*) gene maps; kb, kilobase; PPTase, phosphopantetheinyl transferase; DHBA, 2,3-dihydroxybenzoic acid; Sal, salicylic acid; A, adenylation domain; PKS/NRPS, polyketide synthase/non-ribosomal peptide synthetase; TE-II, type II thioesterase; TBDT, TonB-dependent transporter; DAHP, 3-deoxy-D-arabino-heptulosonate 7-phosphate synthase. (**C**) Piscibactin biosynthesis pathway, as proposed in previous studies (19, 20). Syn, synthase; A, adenylation domain; ACP, aryl carrier protein; Cy, heterocyclization; MT, methyltransferase; PCP, peptidyl carrier protein; PPTase, phosphopantetheinyl transferase; KS, ketosynthase; AT, acyltransferase; KR, ketoreductase; TE, thioesterase.

*Vibrionaceae* exhibit a broad ecological range and genome plasticity, primarily driven by horizontal gene transfer (HGT) (1). In recent decades, changes in geographical distribution and a gradual increase in the incidence of infectious diseases caused by these bacteria have been observed (2, 3). These changes have been linked to climate change and global rise in temperature (2, 3). Production of siderophores is advantageous to bacterial fitness in the environment and is a key virulence factor in relevant pathogenic bacteria (4, 5). Thus, understanding the molecular basis of siderophore biosynthesis, their chemical diversity, and transport mechanisms facilitates designing novel antimicrobial treatments and finding new therapeutic targets (6–11). Piscibactin (Pcb, **1**) is a yersiniabactin-like siderophore encoded by the high-pathogenicity island *irp*-HPI (Fig. 1A and B). Its production has a significant impact on the virulence of pathogenic bacteria that affect fish and mollusks, such as *V. anguillarum* (12, 13), *V. neptunius* (14), and *Photobacterium damselae* subsp. *piscicida* (15, 16). However, this pathogenic island is also found to be widely distributed in other *Vibrionaceae*, including species that are pathogenic to humans (17, 18).

Pcb (**1**), along with its possible biosynthetic intermediate prepiscibactin (Pre-Pcb, **2**), was first isolated from *P. damselae* subsp. *piscicida*, and a tentative biosynthesis pathway was proposed (Fig. 1C) (19). The validation of this pathway, including most enzymatic steps and the characterization of the ferri-piscibactin TonB-dependent outer membrane transporter (TBDT) FrpA, was achieved using the highly virulent *V. anguillarum* RV22 strain as the genetic background (12, 21, 22). The synthesis begins with the conversion of chorismate to salicylate, likely mediated by Irp9 (Fig. 1C) (15), followed by the activation of the salicylate moiety by the aryl acid adenylation enzyme Irp5. However, the functions attributed to Irp9 and Irp5 have not yet been confirmed. Subsequently, the polyketide synthase/non-ribosomal peptide synthetase (PKS/NRPS) hybrid enzymes Irp1 and Irp2 would form the final siderophore. Interestingly, Pcb could correspond to the early release of the nascent siderophore from module 5 of Irp1, mediated by the type II thioesterase (TE-II) Irp4 (21). It is also noticeable that the *irp*-HPI genomic island in *V. anguillarum* lacks the *entD* homolog, which encodes a 4'-phosphopantetheinyl transferase (PPTase) required to activate the thiolation domains of the PKS/NRPSs (23). In *V. anguillarum*, this deficiency is complemented *in trans* by the PPTase VabD, encoded within the vanchrobactin gene cluster (Fig. 1B) (12). This interaction allows for the *V. anguillarum* strains harboring *irp*-HPI island and the vanchrobactin gene cluster to produce the salicylate-type Pcb (**1**) and the catecholate-type vanchrobactin (Vcb, **3**) (Fig. 1A).

Pcb (**1**) and Vcb (**3**) production was described using traditional natural products methods, which involved bio-guided fractionations followed by LC-MS and NMR analyses (19, 24). This approach is time-consuming and often focuses on a limited subset of metabolites. Similarly, genomic studies centered on biosynthetic gene clusters (BGCs) fail to predict potential interactions between biosynthesis routes as the one observed between *irp*-HPI and vanchrobactin gene cluster (25). Recently, a set of five new piscibactin-like siderophores, named photoxenobactins A-E (PxbA-E) (Fig. 1A), was described from the insect symbiont *Photorhabdus*, which harbors homologs of the *irp* genes (26). The authors propose photoxenobactin C as the final product from the enzyme line, while the other analogs may arise from the early release of intermediates (Fig. 1C) (20). The integration of genomics and untargeted LC-MS/MS-based metabolomics offers holistic insights into the diversity of bacterial metabolites and their biosynthesis pathways (27).

This study demonstrates that the combination of cutting-edge metabolomics with genomic insights is an effective strategy that allows the complete characterization of bacterial siderophore metabolomes (siderome(s)) and their correlation with the BGCs. Our findings revealed the production of a rich array of piscibactin-like siderophores by *V. anguillarum*, including members of the photoxenobactin family. The complete characterization of these siderophores showed the presence of unreported catechol Pcb/Pxb-like siderophores. By constructing single and double mutants affecting piscibactin and vanchrobactin siderophore functions, the cooperative interactions between the *irp*-HPI and vanchrobactin pathways at both synthesis and uptake levels were uncovered, contributing to the robustness of iron acquisition mechanisms. This work offers new insights into the biosynthesis of piscibactin–photoxenobactin and the iron uptake routes of different piscibactin–photoxenobactin analogs*,* providing deeper understanding of the virulence pathways of *Vibrionaceae*.

## RESULTS

### *V. anguillarum* siderome includes seven piscibactin/photoxenobactin-like siderophores

**Fig 2:**
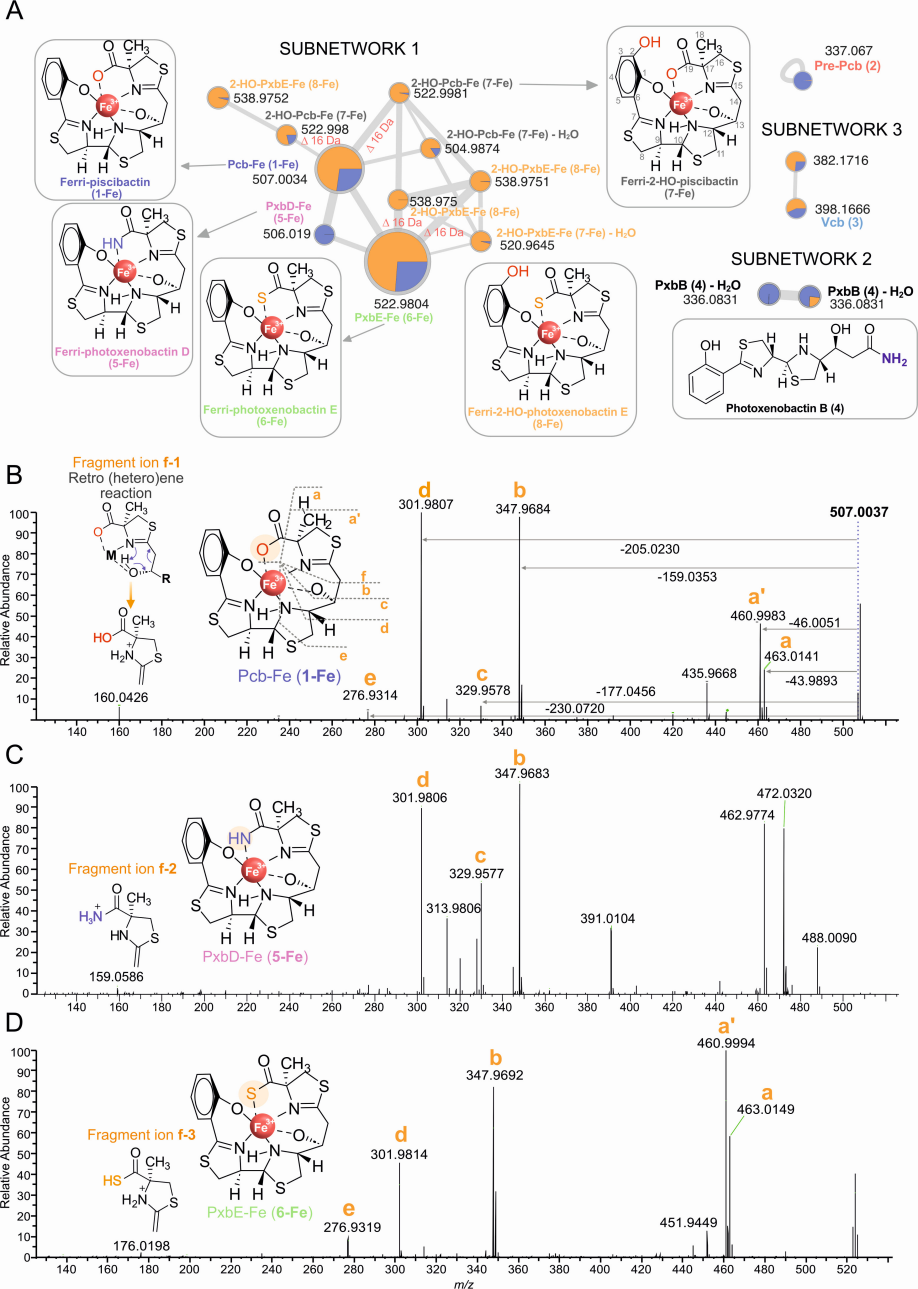
FBMN and MS/MS analysis of Pcb/Pxb-like siderophores in *V. anguillarum* RV22. (**A**) Subnetworks extracted from the cell-free supernatant of *V. anguillarum* RV22. The radius of the nodes refers to the sum intensity of the precursor ion, and edge width varies with the cosine scores. The pie chart inside each node refers to the relative abundance in H3 (orange) and H4 (blue) fractions. Consistent siderophore profiles were observed across the three technical replicates. (**B**) Fragmentation pattern of Pcb-Fe (**1-Fe**). (**C**) Fragmentation pattern of putative PxbD-Fe (**5-Fe**). (**D**) Fragmentation pattern of PxbE-Fe (**6-Fe**).

The cell-free supernatant from *V. anguillarum* RV22 wild-type strain cultured under iron-limiting conditions was fractionated using our SPE-HLB/LC-MS methodology, as described by Souto et al. for the isolation of Pcb (**1**) (19, 28, 29). The CAS-active fractions were subjected to untargeted LC-MS/MS and analyzed by Feature-Based Molecular Networking (FBMN), revealing several molecular subnetworks of closely related compounds (Fig. S1). In the first molecular network, ten nodes related to metal complex ions were detected by screening the iron isotopologue clusters (Fig. 2A, subnetwork 1) throughout the subnetworks. Three of them were tentatively identified as the [M + H]^+^ ions of the iron complexes of piscibactin (Pcb-Fe, **1-Fe**) (19), photoxenobactin E (PxbE-Fe, **6-Fe**), and photoxenobactin D (PxbD-Fe, **5-Fe**) (Fig. 2A) (26). Remarkably, two unidentified nodes at *m/z* 538.9752 and 522.9980 [M + H]^+^ exhibited a mass difference of Δ *m/z* 15.9947 Da to Pcb-Fe (**1-Fe**) and PxbE-Fe (**6-Fe**), respectively, likely indicating the presence of an additional oxygen atom in these novel Pcb/Pxb derivatives. The last two nodes, displaying ions at *m/z* 504.9874 and 520.9645, represent the in-source water-loss fragment ions [M + H-H_2_O]^+^ of these new derivatives (30).

Photoxenobactin B (**4**) was annotated in a second molecular network (Fig. 2A, subnetwork 2) from the [M + H-H_2_O]^+^ ion at *m/z* 336.0831 (calcd. for C_15_H_18_N_3_O_2_S_2_^+^, 336.0835)(26). Additionally, vanchrobactin (Vcb, **3**) (*m/z* 398.1666 [M + H]^+^, calcd. for C_16_H_23_N_5_O_7_^+^, 398.1676) (24) in subnetwork 3, and prepiscibactin (Pre-Pcb, **2**) (*m/z* 337.0670 [M + H]^+^, calcd. for 337.0675) (19), found as a single node, were also annotated (Fig. 2A).

Taken together, these data reveal that *V. anguillarum* primarily produces Pcb (**1**) and PxbE (**6**) in nearly equimolar proportions. According to the radii of the nodes displayed in the FBMN, which refers to the sum intensity of the precursor ion, Pcb (**1**) and PxbE (**6**) represent approximately 60–70% of the total siderophore pool. In contrast, Vcb (**3**) accounts for about 6% of the profile. The novel Pcb/Pxb derivatives make up about 10–15% of the siderome, comprising ~5% 2-OH-Pcb (**7**) and ~10% 2-OH-PxbE (**8**) (see chemical characterization below), while PxbB (**4**) and PxbD (**5**) collectively contribute an additional ~10% (Fig. 2).

### NMR and MS/MS characterization of novel catechol Pcb/Pxb-like siderophores

To elucidate the structure of the putative Pcb/Pxb-like siderophores, the cell-free supernatant from *V. anguillarum* RV22 Δ*vabF*, a mutant unable to produce Vcb (**3**) and known to overproduce Pcb (**1**) (12), was chelated with a gallium salt and submitted to our SPE-HLB/LC-MS methodology (29). Gallium closely mimics iron in terms of ionic radii and binding tendencies (31). Notably, the diamagnetic property of this metal allows the analysis of the gallium complexes by NMR spectroscopy, while its distinctive isotopic pattern (3:2, ^69^Ga:^71^Ga) facilitates their rapid localization by MS, making it a perfect metal core for the study of siderophores. In this way, Pcb-Ga (**1-Ga**) and PxbE-Ga (**6-Ga**), along with the two new Pcb/Pxb-Ga complex analogs, **7-Ga** and **8-Ga**, were purified by HPLC from the HLB-SPE fraction eluted with 1:1 MeCN:H_2_O (HLB-H3 fraction). All the Pcb/Pxb-like analogs exhibited the characteristic isotopic pattern of gallium in their (+)-HRESIMS, confirming their chelating properties and the previously proposed molecular formulas (Fig. S2, S5, S11, and S17) (32). The structures of Pcb-Ga (**1-Ga**) and PxbE-Ga (**6-Ga**) could readily be confirmed by comparing their experimental NMR data with the previously reported data (Table S5) (19, 26, 32). The PxbE-Ga complex (**6-Ga**) was previously reported by us as a tautomeric mixture, where the thiocarboxylic acid terminal can coordinate the Ga(III)-ion through the sulfur (thiol-form, **6A-Ga**) or the oxygen (thione-form, **6B-Ga**) atom (Table S6)(32).

The additional hydroxyl group, already deduced from the MS data, was proposed at position C-2 in **7-Ga** and **8-Ga** based on the comparison of their NMR spectral data with those of Pcb-Ga (**1-Ga**) and PxbE-Ga (**6-Ga**), respectively, indicating the presence of a 1,2,6-trisubstituted aromatic ring in these new analogs (Tables S5 and S6). This was confirmed following the proton-proton coupling constants of the aromatic protons, the absence of the H-2 signals (Fig. S12 and S18), the characteristic carbon chemical shifts of C-2 and C-1 (Fig. S13, S19, and S21), and HMBC correlations (Fig. S16 and S22). As previously observed for PxbE-Ga (**6-Ga**), a tautomeric mixture was also observed between the thiol (**8A-Ga**) and thione forms (**8B-Ga**) of the thiocarboxylic acid present in **8** (Table S6) (32). Due to the presence of this additional hydroxy group at position 2, the new analogs were named as 2-hydroxypiscibactin (2-OH-Pcb, **7**) and 2-hydroxyphotoxenobactin E (2-OH-PxbE, **8**).

MS/MS fragmentation studies of Pcb-Fe complex (**1-Fe**) were performed for the first time to confirm the proposed new structures and support future molecular networking works related to this type of compounds. The MS/MS fragmentation of **1-Fe** (Fig. 2B) displayed the fragment ions at *m/z* 463.0141 and *m/z* 460.9983, originating from the neutral losses of CO_2_ (a) and CH_2_O_2_ (a’) from the terminal carboxylic acid, respectively. The neutral loss of 159.0353 Da (fragment **b**, *m/z* 347.9684) represented the bond cleavage between positions C-12 and C-13, a pattern also observed in yersiniabactin metal complexes (33). This cleavage was supported by the formation of the fragment ion detected at *m/z* 160.0426 (**f-1**) (Fig. 2B). Fragments **c–d** were tentatively proposed as depicted in Fig. 2B. This fragmentation sequence (fragments **a–f**, Fig. 2C and D) was conserved among the different Pcb/Pxb derivatives. Notably, 2-OH-Pcb-Fe (**7-Fe**) and 2-OH-PxbE-Fe (**8-Fe**) showed the same neutral losses, resulting in fragments that were 15.9947 Da higher compared to those of Pcb-Fe (**1-Fe**) and PxbE-Fe (**6-Fe**) (Fig. S23). These new analogs shared the fragments **f-2** and **f-3** (Fig. 2C and D) with Pcb and Pxb, respectively, which agreed with the proposed structure for the new Pcb/Pxb analogs **7** and **8**.

In summary, the new analogs 2-OH-Pcb (**7**) and 2-OH-PxbE (**8**), bearing a 2,3-dihydroxybenzoate moiety instead of the salicylate group, represent the first report of catecholate Pcb/Pxb-like siderophores (Fig. 2A).

### *irp*-HPI genomic island frequently coexists with other PKS/NRPS systems

**Fig 3:**
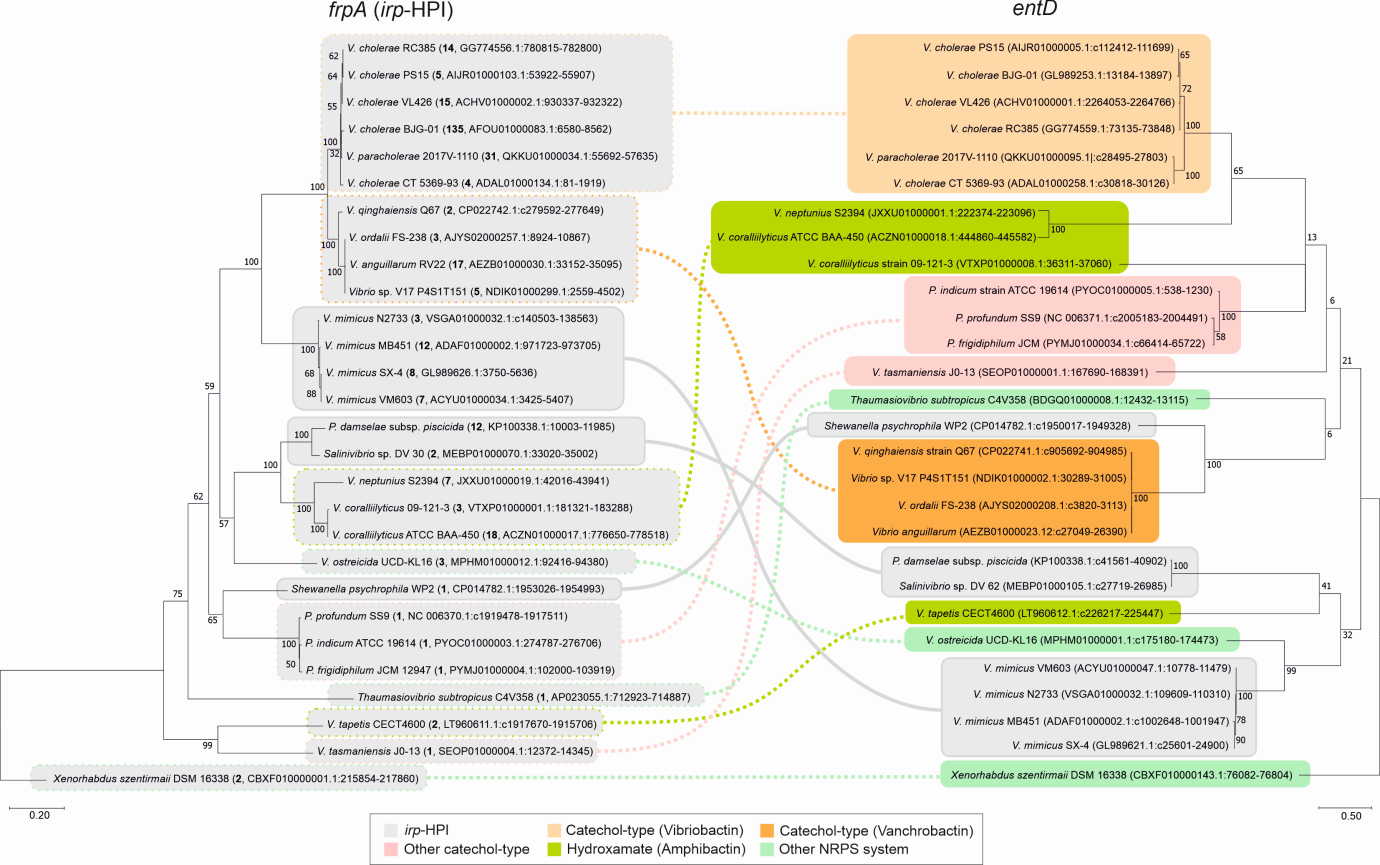
Phylogenetic relationships of the *irp*-HPI genomic island (inferred from a phylogenetic tree of *frpA*) and its counterpart *entD* gene across *Vibrionaceae* based on nucleotide sequences. Phylogenetic reconstruction of the *irp*-HPI island (*frpA*) and *entD* (PPTase) was inferred using the maximum likelihood method and the general time reversible model. The trees with the highest log likelihood (−23034.17 for *frpA* and −12610.04 for *entD*) are shown. Branch lengths are scaled to the number of substitutions per site. Colors indicate the NRPS system in which *frpA* or *entD* is present. A continuous line denotes that the *irp*-HPI element and the PPTase *entD* gene are encoded in the same gene cluster, while a discontinuous line indicates that PPTase is encoded elsewhere in the bacterial genome, outside the *irp*-HPI element. Labels denote species and strain names, the number of identical FrpA sequences (100% coverage and ≥99% identity), and the GenBank accession number.

To explore the diversity of the *irp*-HPI genomic island and its coexistence with other siderophore BGCs, with a specific focus on the location of *entD* homologs encoding PPTase, BLAST searches and genome analyses were conducted in the whole-genome shotgun (WGS) NCBI database. The Pcb/Pxb gene clusters of the genus *Photorhabdus* and the *Vibrionaceae* family are phylogenetically distant, sharing only a slight similarity. Therefore, *frpA* homolog sequences, which encode the Pcb/Pxb TBDT (22), were used to infer the *irp*-HPI phylogenetic relationships using the *Photorhabdus frpA* homolog as outgroup. Subsequently, the *frpA* phylogeny was compared with the phylogeny of the *entD* homologs found in the genome of each *irp*-HPI carrier bacterium (Fig. 3). The results show that EntD functions (PPTase) are typically encoded outside of the *irp*-HPI genomic island, within the bacterial recipient genome. This arrangement results in significant differences between the phylogenies of *frpA* and *entD*. In addition, although some versions of the *irp*-HPI genomic island have closely located *entD* genes, such as those found in *V. mimicus*, *P. damselae* subsp. *piscicida*, *Salinivibrio* sp., or *Shewanella psychrophila*, their phylogenies do not match with their respective *irp*-HPI element (Fig. 3). These results also show that the *irp*-HPI genomic island frequently coexists with other PKS/NRPS systems, primarily catecholate-type siderophores, such as vanchrobactin or vibriobactin (Fig. 3).

### Increased availability of DHBA facilitates the production of catecholate-type Pcb/Pxb

**Fig 4:**
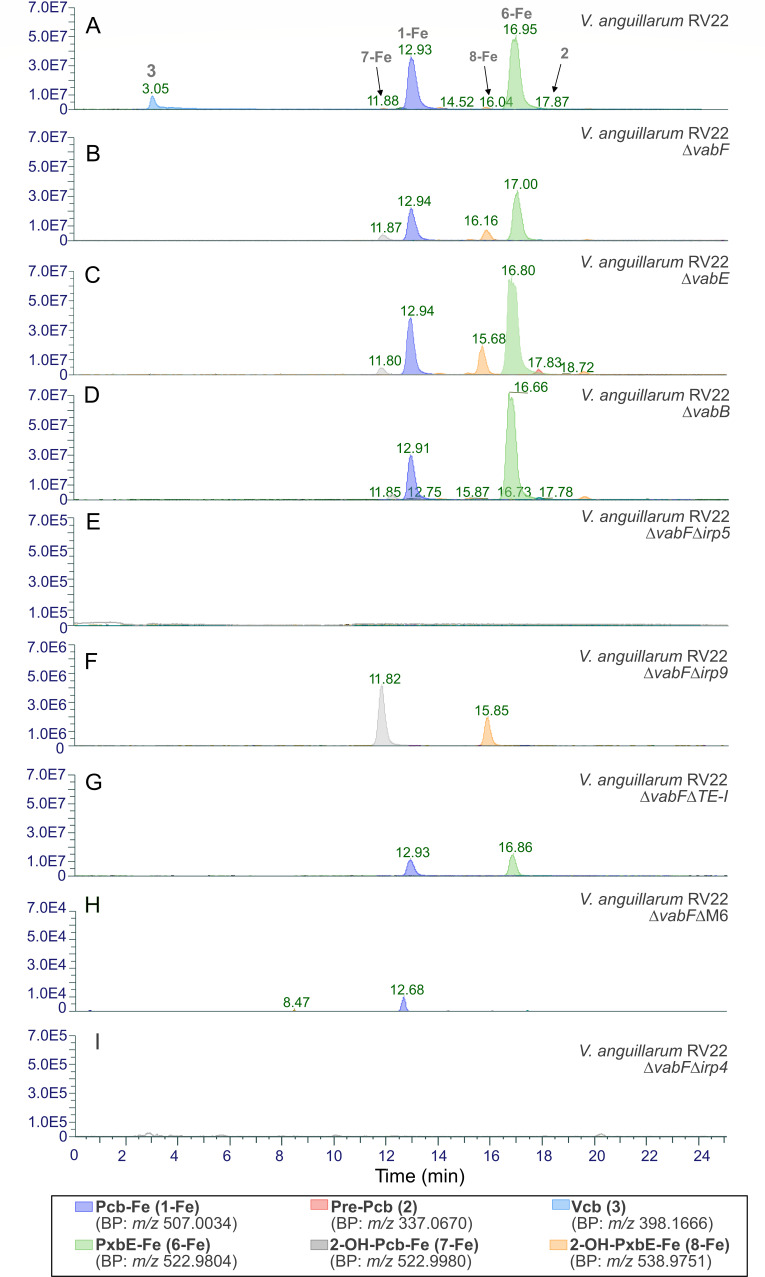
Siderophore composition from the H3 fraction of the *V. anguillarum* RV22 wild type and its defective mutant strains. Overlayed expansions (0–25 min) of extracted ion chromatograms of Pcb-Fe (**1-Fe**), PxbE-Fe (**6-Fe**), 2-OH-Pcb-Fe (**7-Fe**), 2-OH-PxbE-Fe (**8-Fe**), Pre-Pcb (**2**), and Vcb (**3**) of *V. anguillarum* RV22 wild type (**A**), and *V. anguillarum* RV22 Δ*vabF* (**B**), Δ*vabE* (**C**), Δ*vabB* (**D**), Δ*vabF*Δ*irp5* (**E**), Δ*vabF*Δi*rp9* (**F**), Δ*vabF*Δ*TE-I* (**G**), Δ*vabF*Δ*M6* (**H**), and Δ*vabF*Δi*rp4* (**I**) mutant strains. Consistent siderophore profiles were observed across the three technical replicates.

To study the underlying production of the catecholate-type piscibactin (Pcb) and photoxenobactin (Pxb) and the interactions between the Pcb/Pxb and Vcb biosynthetic pathways, the siderome of *V. anguillarum* RV22 Δ*vabF*, *ΔvabE*, and Δ*vabB* mutant strains was analyzed in comparison with the wild-type strain (Fig. 4A through D; Fig. S24 - S27). VabF and VabE are the NRPS enzymes involved in the biosynthesis of Vcb (**3**) by sequentially assembling the intermediates 2,3-dihydroxybenzoate (DHBA), serine, and arginine, while the VabB enzyme is involved in the production of DHBA. Thus, a Δ*vabB* defective mutant only synthesizes residual amounts of DHBA (34).

Vcb (**3**) was not detected in the siderome of the Δ*vabF* and Δ*vabE* mutant strains, which exhibited nearly indistinguishable siderophore profiles (Fig. 4B and C; S25 and S27). They primarily produce Pcb (**1**) and PxbE (**6**) at almost equimolar ratios, constituting 30% and 40% of the siderophores detected in the HLB-H3 fraction, respectively. Most notably, both mutant strains exhibited a significant increase in the production of the catecholate-type Pcb/Pxb, 2-OH-Pcb-Fe (**7-Fe**) and 2-OH-PxbE-Fe (**8-Fe**) (Fig. 4B and C), compared to the wild type (Fig. 4A; Fig. S24). On the other hand, the siderophore metabolome analysis of the Δ*vabB* mutant showed that it only synthesizes minimal amounts of the catecholate-type siderophores Vcb (**3**), 2-OH-Pcb-Fe (**7-Fe**), and 2-OH-PxbE-Fe (**8-Fe**) (Fig. 4D; Fig. S26). These results suggest that the enhanced availability of DHBA in the mutants (Δ*vabF* and Δ*vabE*), which do not deplete it via Vcb (**3**) biosynthesis, facilitates metabolic cross-talk between the piscibactin and vanchrobactin siderophore pathways, leading to the production of novel catecholate-type Pcb/Pxb forms.

### The substrate promiscuity of Irp5 enables the production of catecholate-type Pcb/Pxb

To gain a deeper understanding of the enzymatic mechanisms driving the synthesis of Pcb/Pxb, the initial steps of the pathway were investigated. In particular, the roles of the putative salicylate synthase Irp9 and the aryl acid adenylation enzyme Irp5 were investigated by generating single mutant strains of genes *irp9* and *irp5* in the *V. anguillarum* Δ*vabF* genetic background.

**Fig 5:**
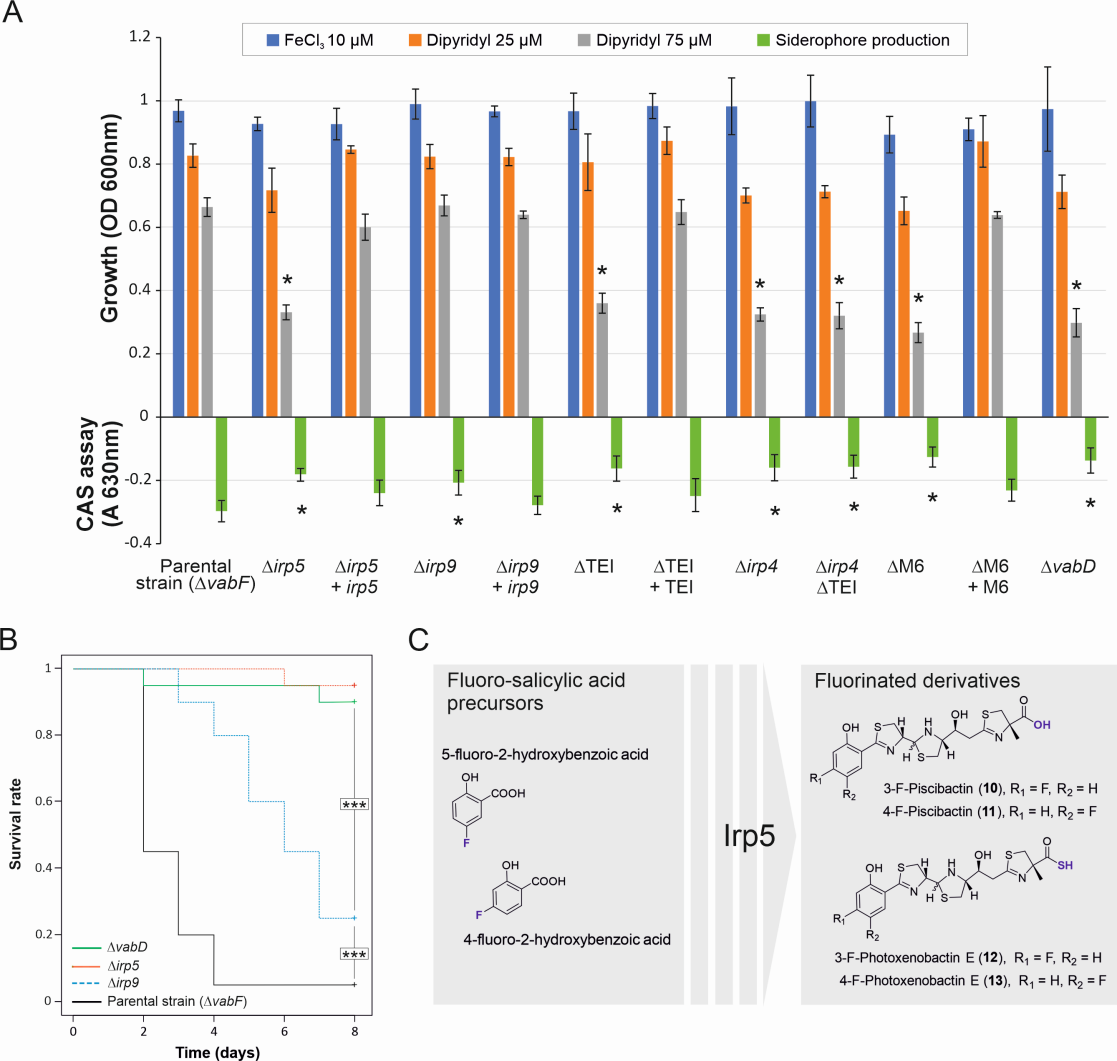
Biological behaviors of the *V. anguillarum* parental strain and its derivative mutants. (**A**) Growth ability and siderophore production (CAS assay) of the *V. anguillarum* parental strain (*ΔvabF*) and its derivative defective mutants. Mean and SD are shown (*n* = 6) and compared using one-way ANOVA. (**B**) Survival dynamics following infection challenge with the parental strain or its derivative mutants in sole fingerlings. Statistical differences were analyzed using the Kaplan–Meier method with the Mantel–Cox log-rank test. (**C**) The biosynthetic flexibility of the Irp5 can be used to produce structurally diverse fluorinated Pcb/PxbE analogs via precursor-directed biosynthesis. Asterisks denote the existence of statistical differences compared to parental strain (*P < 0.05, ***P < 0.001).

The parental Δ*vabF* strain and its derivative mutants, Δ*vabF*Δ*irp9* and Δ*vabF*Δ*irp5*, were challenged to grow under different conditions of iron availability. Parental and mutant strains exhibited similar growth ability under iron-excess conditions (Fig. 5A). However, under iron restriction, the Δ*vabF*Δ*irp5* mutant strain showed reduced growth, which correlates with a lower siderophore production (Fig. 5A). Surprisingly, inactivation of *irp9* resulted in only a slight reduction in siderophore production in the Δ*vabF*Δ*irp9* mutant strain, which retained the same growth ability under iron-restricted conditions as the wild type (Fig. 5A). The complementation of the Δ*vabF*Δ*irp5* and Δ*vabF*Δ*irp9* mutants with their respective wild-type alleles restored the parental phenotype (Fig. 5A). Finally, fish challenge experiments showed distinct virulence outcomes. While the *V. anguillarum* Δ*vabF*Δ*irp9* mutant retained virulence potential, the Δ*vabF*Δ*irp5* mutant was avirulent (Fig. 5B).

The changes in growth and virulence observed in the *ΔvabFΔirp5* and *ΔvabFΔirp9* mutants were found to be related to changes in the HPLC-HRMS analysis, which revealed significant different sideromes (Fig. 4E and F). While the inactivation of *irp5* (Δ*vabF*Δ*irp5* mutant) completely abolished the synthesis of both phenolate- and catecholate-type Pcb/Pxb, the Δ*vabF*Δ*irp9* mutant still produced the catecholate-type Pcb/Pxb **7** and **8** (Fig. 4E and F; Fig. S28 and S29). The results show that catecholate-type Pcb/Pxb **7** and **8** are sufficient to support growth under iron deficiency. They also confirm that Irp9 functions as the salicylate synthase necessary to produce canonical salicylate-like Pcb/Pxb siderophores, Pcb (**1**) and PxbE (**6**). Conversely, Irp5 is required for synthesizing both phenolate- and catecholate-type Pcb/Pxb siderophores. This finding strongly suggests that Irp5 is a versatile aryl acid adenylation enzyme capable of utilizing both the salicylate and the 2,3-dihydroxybenzoate (DHBA) groups as substrates. This versatility was corroborated with a precursor-directed biosynthesis (PDB) analysis in which 5-fluoro-2-hydroxybenzoic acid and 4-fluoro-2-hydroxybenzoic acid were added to two independent cultures of *V. anguillarum* Δ*vabF* (Fig. 5C). HPLC-HRMS studies of both cultures demonstrated the incorporation of both moieties to the structures of Pcb and Pxb-E (Fig. S33 and S34).

### The C-terminal thioesterase (TE) domain of Irp1 and Irp4 is needed to maximize Pcb/Pxb production

A previous study showed that Irp4 is a type II thioesterase (TE-II) required for Pcb (1) production, potentially mediating the release of the siderophore intermediate from module 5 of Irp1 (Fig. 1C) (21). However, the identification of PxbE as the most abundant siderophore (Fig. 4) suggests that this compound would be the final product of the Irp enzymatic pathway (6), which would be produced through a non-canonical release of nascent siderophore from the module 6 of Irp1 (Fig. 1C). In this context, the function of module 6 itself, as well as the role of C-terminal type I TE domain (TE-I) of Irp1 in Pcb/Pxb production, has not yet been studied.

The deletion of the entire module 6 (Δ*M6*) or just the TE-I domain of module 6 (Δ*TE-I*) of Irp1 resulted in a significant reduction of siderophore production when measured by CAS assay, which correlated with a decrease in growth ability under iron deficiency (Fig. 5A). Their phenotypes were equivalent to those observed after the inactivation of Irp4 (Fig. 5A). Further chemical analysis of the siderophore profile by LC-HRMS revealed that, while the deletion of module 6 (Δ*M6*) abolishes the production of PxbE (**6**), this mutant continues to produce Pcb (**1**), albeit at a very low level compared to the parental strain (three orders of magnitude lower) (Fig. 4H; Fig. S31). In contrast, production of Pcb (**1**) was reduced to ~50% in the Δ*TE-I* mutant, which retained production of both Pcb (**1**) and PxbE (**6**) (Fig. 4G; Fig. S30). Finally, evaluation of the *V. anguillarum* Irp4-defective mutant showed that the production of all Pcb/Pxb forms was abolished (Fig. 4I; Fig. S32). These results support the hypothesis that PxbE (**6**) is the result of a non-canonical release from module 6, while Pcb (**1**) is likely to originate from both an earlier release of the intermediate from module 5 and from the chemical conversion of PxbE. The spontaneous hydrolysis of PxbE (**6**) into Pcb (**1**) was recently reported by us (32). Our results also showed that the TE-II Irp4 is required to produce Pcb/Pxb siderophores, but both TE functions, Irp4 and TE-I, are needed to maximize siderophore production.

### Diversification of Pcb/Pxb biosynthesis is supported by the interaction at the uptake level between siderophore transporters

**Fig 6:**
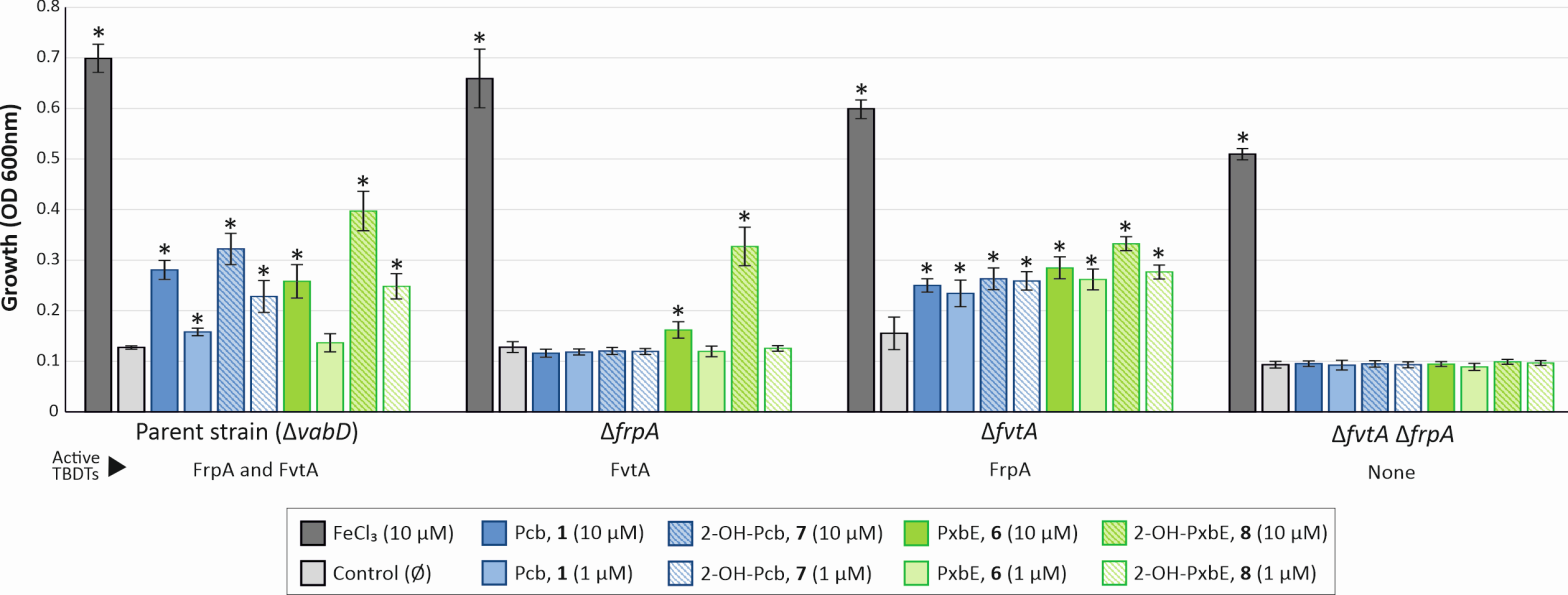
Ability to utilize Pcb/Pxb forms as an iron source of *V. anguillarum* parental strain and its derivative mutants. Growth induction test showing the ability of different *V. anguillarum* genetic backgrounds to utilize each Pcb/Pxb form as an iron source. Mean and SD are shown (*n* = 6) and compared using two-way ANOVA. Asterisks denote the existence of statistical differences compared to parental strain in iron-depleted media (Control) (*P < 0.05).

**Fig 7:**
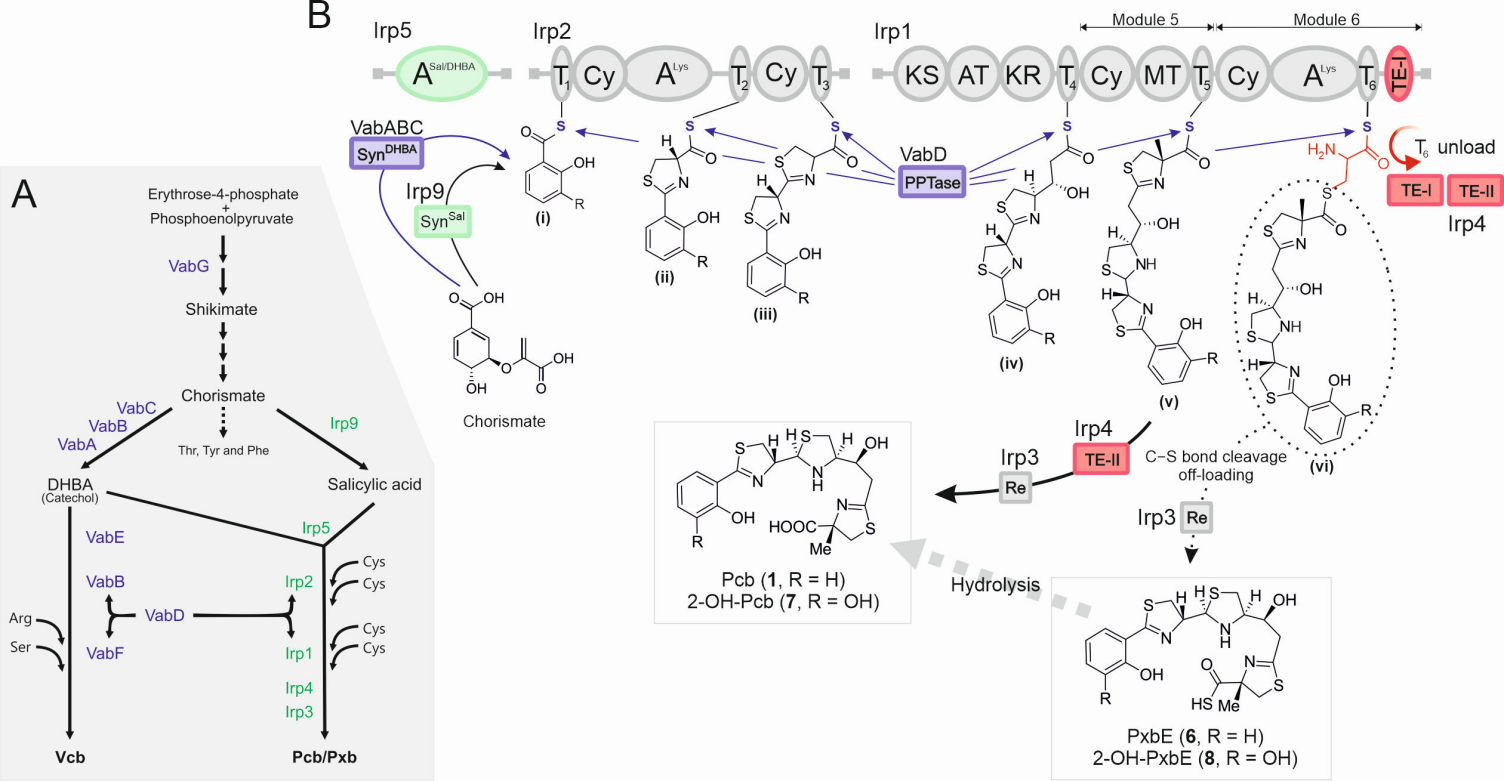
Model of siderophore interactions between piscibactin and vanchrobactin biosynthetic pathways. (**A**) Schematic representation of the interactions between vanchrobactin and piscibactin biosynthesis pathways. Functions encoded within the vanchrobactin gene cluster are denoted in purple, and those within piscibactin in green. (**B**) Updated model of the piscibactin biosynthesis pathway. Syn, synthase; A, adenylation domain; T, thyolation; Cy, heterocyclization; MT, methyltransferase; PCP, peptidyl carrier protein; PPTase, phosphopantetheinyl transferase; KS, ketosynthase; AT, acyltransferase; KR, ketoreductase; TE, thioesterase; TE-I, type I thioesterase; TE-II, type II thioesterase; Re, reductase.

To investigate the biological activity and uptake mechanism of each Pcb/Pxb form, growth induction tests were performed using the *V. anguillarum* Δ*vabD* mutant strain (Fig. 6). Due to VabD being the PPTase required by both Vcb and Pcb/Pxb biosynthetic routes (Fig. 7A), this strain was impaired in siderophore production but retained active piscibactin and vanchrobactin transport systems (12). Thus, the ability to use a compound as an iron source is directly related to the growth induction of the indicator strain, as shown in Fig. 6. The addition of iron (FeCl_3_) or any of the purified Pcb/Pxb forms (Pcb (**1**), 2-OH-Pcb (**7**), PxbE (**6**), or 2-OH-PxbE (**8**)) to the culture medium caused a concentration-dependent growth induction in the *V. anguillarum* Δ*vabD* strain (Fig. 6).

In addition, its derivative TBDT mutants Δ*fvtA*, Δ*frpA*, and Δ*fvtA*Δ*frpA* were also used as indicators considering that FvtA and FrpA are the TBDTs required for the uptake of Vcb and Pcb, respectively (21, 35). Consistently, deletion of *frpA* (*ΔfrpA* mutant) resulted in the inability to utilize Pcb as an iron source, with only residual activity observed for PxbE (**6**). The *ΔfrpA* mutant was also impaired in utilizing 2-OH-Pcb (**7**), but efficiently used 2-OH-PxbE (**8**) as iron source. These results suggest that the 2-OH-PxbE iron complex (**8-Fe**) is likely internalized via an alternative transporter(s) rather than through piscibactin TBDT FrpA. Evaluation of the Δ*fvtA* mutant strain, carrying a deletion on the vanchrobactin TBDT FvtA (35), showed its ability to use all Pcb/Pxb forms as iron sources (Fig. 6). Interestingly, while both Δ*frpA* and Δ*fvtA* single mutant strains utilized 2-OH-PxbE (**8**), the double mutant Δ*frpA*Δ*fvtA* was not able to use it. This indicates that Pcb (**1**), PxbE (**6**), and 2-OH-Pcb (**7**) utilize the piscibactin TBDT FrpA as their entry route, whereas 2-OH-PxbE (8) is efficiently internalized by both the piscibactin FrpA and vanchrobactin FvtA TBDTs.

## DISCUSSION

Insights into the metabolome of pathogenic bacteria can provide valuable information and may facilitate the identification of new therapeutic targets. In this study, the siderophore diversity (siderome) of the highly pathogenic *V. anguillarum* RV22 strain, harboring the *irp*-HPI pathogenicity island, was described using modern FBMN computational tools. This investigation unveiled the production of several Pcb/Pxb-type compounds by *V. anguillarum*, including Pcb (**1**), Pre-Pcb (**2**), PxbB (**4**), PxbD (**5**), and PxbE (**6**), as well as two novel derivatives, 2-OH-Pcb (**7**) and 2-OH-PxbE (**8**), characterized by the presence of an additional hydroxyl group in the aromatic moiety. PxbE (**6**), 2-OH-Pcb (**7**), and 2-OH-PxbE (**8**) exhibited siderophore activity, efficiently chelating both iron and gallium. Additionally, the MS/MS fragmentation behavior of these Pcb/Pxb type compounds was reported for the first time (Fig. 2B through D; Fig. S23). Interestingly, complete characterization by NMR and MS/MS of the two new Pcb/Pxb derivatives as their gallium complexes showed that they are the catecholate-type forms of Pcb and PxbE, which were named as 2-hydroxypiscibactin (2-OH-Pcb, **7**) and 2-hydroxyphotoxenobactin E (2-OH-PxbE, **8**) (Fig. 2A). Pxbs were recently identified in the insect symbiont *Photorhabdus* and are believed to be the result of the unusual release of intermediates from the piscibactin PKS/NRPS (Fig. 7B) (20, 26). Curiously, the earlier research did not indicate any metal-chelation properties for photoxenobactin E (**6**). This study is pioneering in showcasing siderophore activity for this compound and other derivatives.

In *V. anguillarum*, the production of Pcb (**1**) and PxbE (**6**) was observed at nearly equimolar ratios, whereas other piscibactin-like forms, such as PxbB (**4**) and PxbD (**5**), were detected in significantly smaller quantities (Fig. 4A). *Xenorhabdus szentirmaii* mainly produces PxbD (**5**), PxbC (**7**), and Pcb (**1**), while the production of PxbE (**6**) was only detected when the *irp* genes were overexpressed in a Δ*hfq* mutant (26, 36). *hfq* is a global post-transcriptional regulator widespread in bacteria, with pleiotropic effects on bacterial fitness and secondary metabolite production (36). Analysis of the *V. anguillarum* siderome strongly suggests that the final product of the *Vibrionaceae* piscibactin pathway is PxbE (**6**) (Fig. 7B). PxbE (**6**) differs from Pcb (**1**) by having unusual thiocarboxylic acid terminus instead of a carboxylic acid group (Fig. 1A). This terminus is rarely found in PKS/NRPS natural product biosynthesis (23). Its liberation from Irp1 was proposed to occur through a spontaneous C–S bond cleavage of the intermediate vi (Fig. 7B), arising from low catalytic efficiency of its *C*-terminal (type I) TE domain (20). This type of release mechanism would leave a T6 domain-bound residue attached to module 6 of Irp1, impeding subsequent rounds of biosynthesis. In the entomopathogenic bacteria *X. szentirmaii*, the effects of inactivating T_5_ (module 5) or T_6_ domains (module 6) on siderophore production differ. While the T_5_ domain (module 5) is required to produce Pcb and Pxb(s), the T_6_ domain (module 6) mutant strain retains the ability to produce Pcb (**1**) (20). Our results corroborate these findings, as the deletion of module 6 abolished the production of PxbE (**6**) but not Pcb (**1**). This is consistent with the early liberation of “intermediate v” by Irp4 (type II TE), resulting in the formation of Pcb (**1**) (Fig. 7B). However, the production of Pcb (**1**) is lower (three orders of magnitude) than in the parental strain (Fig. 4I), which suggests that most piscibactin is derived from post-assembly-line conversion of PxbE (**6**) (Fig. 7B)(20, 32). The spontaneous hydrolysis of the thiocarboxylic acid in PxbE (**6**) into the carboxylic acid in Pcb (**1**) was already demonstrated in the analysis of the tautomeric forms of our Ga coordination studies of PxbE (**6**) (32) and in other previous studies (37, 38).

TE functions mediate the canonical release of PKS/NRPS products by hydrolyzing the thioester bond that links the growing polyketide or peptide chain to the enzyme (23). Therefore, the piscibactin biosynthesis machinery includes Irp4, an external type II TE, along with a *C*-terminal (type I) TE domain within the enzyme Irp1 (TE-I) (21). In *X. szentirmaii*, type II TE (PxbI) inactivation only led to a decrease in production titers, indicating that it is not required to produce Pcb nor Pxb(s) (20). Conversely, our findings in *V. anguillarum* showed that Irp4 inactivation completely disables siderophore production (Fig. 4I). Therefore, we hypothesized that Irp4 plays a role in the liberation of the “intermediate v” (Fig. 7B) (21). Our results support this hypothesis and strongly suggest that Irp4 plays a dual role in piscibactin liberation (intermediate v) and in unloading (recycling) the T6 domain (module 6) of the Irp NRPS/PKS enzymatic pathway. Furthermore, our findings indicate that the *C*-terminal TE domain of Irp1 (TE-I) has low enzymatic activity but is essential for achieving maximal siderophore production (Fig. 4G). Thus, it likely cooperates with the broad-spectrum TE Irp4 in unloading the residue attached to the T6 domain after PxbE (**6**) release (Fig. 7B), which otherwise impedes subsequent rounds of biosynthesis.

Additionally, interaction(s) between piscibactin and vanchrobactin biosynthetic routes were observed, leading to the production of the catecholate forms of Pcb and PxbE, 2-OH-Pcb and 2-OH-PxbE (Fig. 7B). This was confirmed through mutant library screening. Our results showed that Irp9 is a bifunctional salicylate synthase required for salicylate synthesis, while Irp5 is a salicylate-activating enzyme that is involved in the initial steps of piscibactin NRPS/PKS assembling line. More notably, we proved that it is a promiscuous aryl acids adenylation enzyme that can adenylate both salicylate and DHBA moieties. The production of diverse siderophore-related compounds typically involves substrate-tolerant enzymes. Examples of such enzymes include the acyl transferase DesC (39) and adenylating enzyme AmoG (40), which play a role in the synthesis of deferoxamine and amonabactin, respectively. The substrate promiscuity of Irp5 enables the production of catecholate-type Pcb/Pxb, and in this study, it was used to generate piscibactin analogs of fluoro-salicylic compounds by precursor-directed biosynthesis. In summary, we have demonstrated that the biosynthetic flexibility of Irp5 offers a strategy for generating Pcb/Pxb analogs that can be used for drug development (41–43).

Ferri-siderophore complexes are acquired through highly specific transporters (44). Therefore, the synthesis of innovative siderophores must meet the requirements of the ferri-siderophore uptake machinery to be used as iron source. The main specificity resides in the outer membrane TBDTs, with each transporter exhibiting varying degrees of ligand flexibility (45). In *Aeromonas salmonicida* subsp. *salmonicida*, the production of a mixture of amonabactin analogs, each with significant structural differences, is supported by the broad ligand plasticity of the amonabactin transporter FstC (46). Prior research has shown that FrpA is the outer membrane (OM) TonB-dependent transporter (TBDT) required for the utilization of ferri-piscibactin (22), while FvtA is the OM TBDT for ferri-vanchrobactin (35). The Pcb/Pxbs-like analogs described here exhibit similar biological activities, as evidenced by their ability to promote the growth of *V. anguillarum* (Fig. 6). Our results confirmed that the piscibactin forms are specifically internalized through FrpA (Fig. 6). However, the catecholate siderophore 2-OH-PxbE-Fe (**6-Fe**) is also internalized through the vanchrobactin transporter FvtA (35) (Fig. 6). Thus, the interaction between Pcb/Pxb and catecholate-type vanchrobactin systems occurs at both biosynthesis and uptake levels.

The evolutionary advantage of harboring two siderophore systems is unclear, as it would impose a high energetic cost (47, 48). The simultaneous presence of *irp*-HPI and other active siderophore systems suggests that multiple systems enhance bacterial fitness. The ability to produce diverse siderophores with different iron-chelating groups may provide an advantage in fluctuating environments (49–51) and could enhance virulence capabilities (52–57). *In vivo* infection results are consistent with the phenotypes observed *in vitro*, as the production of catecholate Pcb/PxbE forms relatively maintains biological behaviors, including virulence properties (Fig. 5). This demonstrates that the interplay between siderophore systems, specifically piscibactin and vanchrobactin, not only enhances the *Vibrio* siderophore metabolome but can also preserve its virulence potential against the effects of inactivating mutations. While other examples exist of separate genetic loci encoding secondary metabolites and dependencies between siderophore systems (58, 59), the crosstalk reported here occurs at both biosynthetic and uptake levels, driving siderophore diversification. Collectively, genomic analysis showed that the phenolate-type Pcb/Pxb often coexists with other siderophore systems in the recipient genome, and that they are primarily catecholate-type siderophore systems, such as vibriobactin in *V. cholerae* (Fig. 3). Similarly, in other *Vibrionaceae* such as *V. neptunius* (14) and *V. corallyliticus*, piscibactin coexists with the hydroxamate-type siderophore amphibactin (Fig. 3). Vibriobactin and amphibactin are among the most widespread siderophores in marine bacteria (14, 60–62). Thus, the present results could be extended to a wide variety of *Vibrionaceae* harboring the *irp*-HPI genomic island. Additionally, although some versions of the *irp*-HPI genomic island have *entD* genes (PPTase functions) located closely, their phylogeny does not match that of the *irp*-HPI. This observation reinforces the hypothesis that they have been recruited by natural selection to be closely associated with the *irp*-HPI genomic island (16).

This work sheds light on the interactions between siderophore systems, leading to innovative compounds. By integrating modern new-generation metabolomic analyses, we achieved a comprehensive characterization of *V. anguillarum* siderome, which includes new catecholate Pcb/Pxb-like siderophores. The Pcb salicylate-activating enzyme Irp5 exhibits substrate promiscuity, leading to the formation of catechol Pcb/PxbE-type siderophores. Ecologically, the coexistence and interaction of different siderophore systems enhance bacterial fitness and protect against inactivating mutations. Most notably, the substrate promiscuity of Irp5 could be exploited to generate piscibactin analogs via precursor-directed biosynthesis, offering new alternatives for developing antimicrobials against diverse pathogenic *Vibrio* species.

## MATERIALS AND METHODS

### Bacterial strains, plasmids, and media

The bacterial strains and plasmids used in this work are listed in Table S1. Routinely, *V. anguillarum* strains were grown at 25°C or 15°C in Tryptic soy broth (TSB) or agar (TSA) (Condalab) supplemented with up to 1% NaCl. *E. coli* strains were grown in Luria-Bertani (LB) broth or agar (Condalab) at 37°C. When required, the medium was supplemented with the appropriate antibiotic at the final concentrations of ampicillin sodium salt (Amp) at 60 µg mL^−1^ or 100 µg mL^−1^ and kanamycin (Kan) at 50 µg mL^−1^.

### Construction of *irp5*, *irp9*, TE-I, and M6 defective mutants by allelic exchange and mutant complementation

In-frame deletion mutants of *irp5*, *irp9,* TE-I, and M6 were constructed by allelic exchange in *V. anguillarum* Δ*vabF* background (mutant strain impaired for vanchrobactin synthesis). The flanking regions of *irp5*, *irp9*, and the thioesterase domain, and module 6 of *irp1* were PCR amplified and cloned into the low-copy cloning vector pWKS30 (63). The constructions were digested with *NotI* and *ApaI* and ligated into the suicide vector pNidKan (64). The resultant plasmids were conjugated with *V. anguillarum* Δ*vabF*. After two recombination events, the transformants were selected based on 15% sucrose resistance. Plasmid loss was confirmed by screening growth in ampicillin and kanamycin plates. PCR was used to confirm the allelic exchange. This process led to the generation of the mutant strains RV22 Δ*vabF*Δ*irp5*, RV22 Δ*vabF*Δ*irp9*, RV22 Δ*vabF*Δ*TE-*I, and RV22Δ*vabF*Δ*M6*. For the generation of RV22 Δ*vabF*Δ*irp4*Δ*TE-I*, RV22 Δ*vabD*Δ*fvtA*, and RV22 Δ*vabD*Δ*frpA*Δ*fvtA* mutants, the plasmids pML1320 and pMB42 were mobilized by conjugation to ML178, MB67, and ML210, respectively, and we proceeded as described above. For mutant complementation, the wild-type genes were PCR amplified, digested with *NotI* and *ApaI*, and cloned into the suicide vector pNidKan. Gene complementation was performed as described above for mutant construction. The oligonucleotides used in this work are listed in Table S2.

### Growth ability and siderophore production under iron-restricted conditions

*V. anguillarum* parental strain RV22 Δ*vabF*, its derivative mutants Δ*irp5*, Δ*irp9*, Δ*TE-I*, Δ*M6*, Δ*irp4*, and Δ*irp4*Δ*TE-I*, and the corresponding complemented strains were grown in TSB-1 at 25°C overnight. The OD_600_ was measured and adjusted to 0.5, and a 1:50 dilution was inoculated in 5 mL of CM9 minimal medium. To achieve iron excess, the medium was supplemented with 10 µM FeCl_3_, whereas 25 or 75 µM 2,2’-dipyridyl was added to achieve mild or strong iron-deficient conditions. The cultures were incubated at 15°C, shaking at 120 rpm for 48 h. Final growth (OD_600_) was measured using a spectrophotometer (Hitachi). Additionally, the chrome azurol-S (CAS) liquid assay was used to quantify siderophore production (65). To normalize siderophore production to bacterial growth, an aliquot of cultures grown with 25 µM 2,2’-dipyridyl to an OD_600_ of 0.7 was centrifuged at 6,000 × *g*, and the resulting supernatant was mixed 1:1 with CAS reagent and incubated for 15 min at room temperature. The A_630_ was measured in a spectrophotometer (Hitachi). The mutant strain RV22 Δ*vabD*, unable to produce both piscibactin and vanchrobactin, was used as a control.

### Fish infection challenge

Experimental infections were performed using Senegalese sole (*Solea senegalensis*) fingerlings as model organisms. Fish with an average weight of 10 g were purchased from a fish hatchery in Spain. Upon arrival, they were acclimatized in 50 L tanks with filtered seawater in recirculation, continuous aeration at 18°C, and were fed a commercial diet. Colonies from a 24 h TSA-1 plate of RV22 Δ*vabF*, Δ*vabD,* Δ*vabF*Δ*irp5,* and Δ*vabF*Δ*irp9* were resuspended in saline solution (0.85% NaCl) to achieve a cell suspension with an OD_600_ = 0.5. Fish (*n* = 30) were intraperitoneally injected with 100 µL of the bacterial suspension at a final dose of 5 × 10^5^ CFU/fish of either *V. anguillarum* parental strain or derivative mutant. Bacterial dose was confirmed by plating serial dilutions of the suspension in TSA-1. A control group was injected with 100 µL of saline solution. Fish were monitored, and mortality was daily registered for eight days after injection. Statistical differences in percentage of survival were determined using the Kaplan-Meier method with Mantel-Cox log-rank test using R 4.3.0. *P*-values were significant when *P* < 0.05. The protocols for animal experimentation were performed according to the European and Spanish legislation and were approved and reviewed by the Animal Ethics Committee of the University of Santiago de Compostela (Protocol N° 15012/2022/001).

### General analytical methods and procedures

The solvents used in the HPLC-HRMS and HPLC analyses were LC/MS and LC grade, respectively. MeCN employed for the fractionation was LC analysis grade (Fisher). Deionized water (MQ-H_2_O) was obtained from a Direct-Q system (Merck). ^1^H, ^13^C, and 2D NMR spectra were recorded on a Bruker Avance 500 (500 MHz for ^1^H and 125 MHz for ^13^C) with a dual cryoprobe or a BBI probe. CD_3_OD, CDCl_3_, and DMSO-*d*_6_ were used as deuterated solvents. Chemical shifts are reported in *δ _*scale relative to CD_3_OD (*δ *3.31 ppm for ^1^H NMR, *δ *49.0 ppm for ^13^C NMR), DMSO-d_6_ (*δ *2.50 ppm for ^1^H NMR, *δ *39.52 ppm for ^13^C NMR), and CDCl_3_ (*δ *7.26 ppm for ^1^H NMR, *δ *77.16 ppm for ^13^C NMR). Multiplicities of ^13^C signals were obtained by DEPT. HPLC separation was performed on an Agilent 1100 and 1200 equipped with a solvent degasser, quaternary pump, and an UV detector (Agilent Technologies, Waldbronn, Germany) using an Atlantis dC18 (100 × 4.6 mm, 5 µm, Waters) column. HRMS, HPLC-HRMS, and HPLC-MS/MS data were acquired on a LTQ-Orbitrap Discovery mass spectrometer coupled to an Accela HPLC (Thermo Scientific). OASIS® HLB cartridge (35 cc, 6 g) was used for SPE fractionation.

### Extraction, fractionation, and HPLC-DAD-MS analysis of *V. anguillarum* wild-type strain and derivative mutants

*V. anguillarum* RV22 wild-type strain and derivative mutants Δ*vabE*, Δ*vabB*, Δ*vabF*, Δ*vabF*Δ*irp5*, Δ*vabF*Δ*irp9*, Δ*vabF*Δ*irp4*, Δ*vabF*Δ*TE-I*, and Δ*vabF*Δ*M6* were grown in TSB-1 at 25°C overnight. A 1:100 dilution was inoculated in 1 L of CM9 medium supplemented with 30 µM 2,2’-dipyridyl and incubated at 15°C with shaking at 120 rpm for four days. The cells were pelleted by centrifugation for 30 min at 6000 × g, and the cell-free supernatants were filtered using a PTHK 100 k polyethersulfone Prep/Scale TM- TFF cartridge (Millipore). One liter of cell-free supernatant was extracted and analyzed using our SPE-HLB/LC-HRMS methodology following the protocol described by Souto et al. (19). The CAS-active HLB-H3 fractions (1 mg·mL^−1^), eluted with a 1:1 MeCN:H_2_O mixture from a HLB cartridge, were profiled via HPLC-DAD-MS using an Atlantis dC18 (100 × 4.6 mm, 5 µm, Waters) column set at 20°C, 10 µL of injection volume, and a mobile phase consisting of an isocratic step of 2 min at 20% MeCN in H_2_O (v/v), 20% to 60% MeCN over 18 min, 60% to 95% MeCN over 5 min, 95% MeCN isocratic for 5 min, 95 to 20% MeCN over 2 min, and a final isocratic step at 20% MeCN over 3 min at a flow rate of 0.5 mL·min^−1^. Mass spectra were acquired in positive mode, with resolution set to 30,000 in the range of *m/z* 150–1,500. MS parameters were set as depicted in Table S3. The PDA was recorded in the range 200–400 nm, with a scan bandwidth of 9 nm, a scan rate of 20 Hz, and a scan step of 1 nm. Two biological replicates were analyzed for Δ*vabF*Δ*irp4* and Δ*vabF*Δ*irp5* mutants to confirm the complete loss of Pcb/Pxb production. The quantification of siderophores was based on relative LC-MS/MS peak area comparisons.

### Chemical analysis of the siderophore content of *V. anguillarum* RV22 by feature-based molecular networking (FBMN)

For untargeted metabolomics, HLB fractions H3 (eluted with a 1:1 MeCN:H_2_O mixture from a HLB cartridge) and H4 (eluted with a 7:3 MeCN:H_2_O), obtained from the centrifuged cell-free supernatant chelated with Fe^3+^ of the RV22 wild-type strain, were analyzed by LC-MS/MS using an Atlantis dC18 (100 × 4.6 mm, 5 µm, Waters) column with a mobile phase consisting of 20% to 90% MeCN in H_2_O (v/v) over 30 min, 90% MeCN isocratic over 15 min, 90 to 20% MeCN over 5 min, and a final isocratic step at 20% MeCN at a flow rate of 0.5 mL·min^−1^. MS parameters were set as indicated in Table S3. Data were collected in the data-dependent acquisition (DDA) mode, in which the five topmost intense ions, with a minimum signal threshold of 10^5^ counts, were submitted to high-resolution tandem mass spectrometry (HRMS/MS) analysis via collision-induced dissociation (CID) fragmentation (Table S3). A sample of HLB fractions H3, H4, and a blank was each analyzed in triplicate using the same chromatographic gradient. After each sample, a cleaning protocol of 20–90% aqueous MeCN + 0.1% formic acid (FA) over 20 min was added to avoid retention of siderophores on the column.

Data were analyzed using Thermo Xcalibur software, converted to mzXML using the software MSconvert, and imported to MZmine 3.3.0. Preprocessing in MZmine included mass detection, ADAP chromatogram building and deconvolution, alignment of feature lists, which were filtered twice to remove duplicate peaks and to retain only features with MS2 scans. The specific preprocessing parameters are listed in Table S4.

A Feature-Based Molecular Network (FBMN) was generated using the Global Natural Product Social Molecular Networking (GNPS) online platform (GNPS job link: https://gnps.ucsd.edu/ProteoSAFe/status.jsp?task=b553af7e2b92410ca231397cbd94a030). The parent mass tolerance and MS/MS fragment ion tolerance were set both at 0.02 Da, the cosine score at above 0.7, and matched peaks above 6. Spectra were retained only if the nodes appeared in each other’s respective top 10 most similar nodes. The spectra in the network were then searched against GNPS spectral libraries using a cosine score above 0.7 and at least six matched peaks. Relative siderophore quantification was based on the radii of the nodes displayed in the FBMN, which refers to the sum intensity of the precursor ion. The molecular network was visualized using Cytoscape software.

### Incorporation studies of 4-fluoro-2-hydroxybenzoic acid and 5-fluoro-2-hydroxybenzoic acid

For the determination of precursor utilization, *V. anguillarum* RV22 Δ*vabF* mutant strain was grown in CM9 minimal medium supplemented with 30 µM 2,2’-dipyridyl until mid-exponential phase. The substrates 4-fluoro-2-hydroxybenzoic acid or 5-fluoro-2-hydroxybenzoic acid (BLD PHARMATECH GmbH) were added as a solution in DMSO with a final concentration of 100 µM. The cultures were incubated at 15°C, shaking at 120 rpm. After 48 h, the cells were pelleted by centrifugation at 6,000 × *g* for 30 min, and the cell-free supernatant was filtered using a PTHK 100 k polyethersulfone Prep/Scale TM- TFF cartridge (Millipore). The cell-free supernatants were fractionated and analyzed as mentioned above. 3-Fluoro-piscibactin-Fe (**10-Fe**) was detected at a *t*_*R*_ 13.96 min, and 3-fluoro-photoxenobactin E-Fe (**12-Fe**) was detected at a *t*_*R*_ 17.61 min (see Fig. S34). Similarly, 4-fluoro-piscibactin-Fe (**11-Fe**) was detected at a *t*_*R*_ 13.68 min, and 4-fluoro-photoxenobactin E-Fe (**13-Fe**) was detected at a *t*_*R*_ 17.50 min (see Fig. S33 and S34).

3-Fluoro-piscibactin-Fe (**10-Fe**): HR-ESIMS *m/z* 524.9932 [M + H]^+^ (calculated for C_19_H_20_FN_3_O_4_S_3_Fe^+^, 524.9944).

3-Fluoro-photoxenobactin E-Fe (**12-Fe**): HR-ESIMS *m/z* 540.9705 [M + H]^+^ (calculated for C_19_H_20_FN_3_O_3_S_4_Fe^+^, 540.9715).

4-Fluoro-piscibactin-Fe (**11-Fe**): HR-ESIMS *m/z* 524.9929 [M + H]^+^ (calculated for C_19_H_20_FN_3_O_4_S_3_Fe^+^, 524.9944).

4-Fluoro-photoxenobactin E-Fe (**13-Fe**): HR-ESIMS *m/z* 540.9698 [M + H]^+^ (calculated for C_19_H_20_FN_3_O_3_S_4_Fe^+^, 540.9715).

### Isolation of the Fe(III) complexes of piscibactin (1-Fe), photoxenobactin E (6-Fe), and their catecholate derivatives (7-Fe and 8-Fe) from *V. anguillarum* wild-type strain 

Fraction HLB-H3, obtained from the centrifuged cell-free supernatant chelated with Fe^3+^ of the RV22 wild-type strain, was subjected to RP-HPLC on a semipreparative Atlantis dC18 (100 × 10 mm, 5 µm) with a gradient of MeCN in H_2_O from 15:85 to 60:40 over 30 min at a flow rate of 1.5 mL min^−1^ to yield four pure compounds: 2-OH-Pcb-Fe (**7-Fe**) (t_R_ 18.4 min), Pcb-Fe (**1-Fe**) (t_R_ 19.9 min), 2-OH-PxbE-Fe (**8-Fe**) (t_R_ 21.0 min), and PxbE-Fe (**6-Fe**) (t_R_ 25.9 min).

Piscibactin-Fe (**1-Fe**): HR-ESIMS *m/z* 507.0031 [M + H]^+^ (calculated for C_19_H_21_N_3_O_4_S_3_Fe^+^, 507.0044); UV λ_max_ 229 nm, 258 nm, 308 nm, 388 nm.

2-Hydroxypiscibactin-Fe (**7-Fe**): HR-ESIMS *m/z* 522.9980 [M + H]^+^ (calculated for C_19_H_21_N_3_O_5_S_3_Fe^+^, 522.9993); UV λ_max_ 231 nm, 275 nm, 346 nm.

Photoxenobactin E-Fe (**6-Fe**): HR-ESIMS *m/z* 522.9807 [M + H]^+^ (calculated for C_19_H_21_N_3_O_3_S_4_Fe^+^, 522.9815); UV λ_max_ 231 nm, 255 nm, 315 nm.

2-Hydroxyphotoxenobactin E-Fe (**8-Fe**): HR-ESIMS *m/z* 538.9753 [M + H]^+^ (calculated for C_19_H_21_N_3_O_4_S_4_Fe^+^, 538.9764); UV λ_max_ 229 nm, 269 nm, 347 nm.

### Isolation of the Ga(III) complexes of piscibactin (1-Ga), photoxenobactin E (6-Ga), and their catecholate derivatives (7-Ga and 8-Ga) from *V. anguillarum* Δ*vabF* mutant

Fraction HLB-H3, obtained from the centrifuged cell-free supernatant chelated with Ga^3+^ from the mutant strain *V. anguillarum* RV22 Δ*vabF*, was subjected to RP-HPLC in an Agilent 1100 HPLC coupled to a PDA detector. The purification was carried out in a semipreparative Atlantis dC18 column (100 × 10 mm, 5 µm, Waters) with a gradient of MeCN in H_2_O from 15:85 to 60:40 over 30 min at a flow rate of 1.5 mL min^−1^ to yield Ga-complexes 2-OH-Pcb-Ga (**7-Ga**, t_R_ 15.7 min), Pcb-Ga (**1-Ga**, t_R_ 17.2 min), 2-OH-PxbE-Ga (**8A/B-Ga**, t_R_ 22.2 min), and PxbE-Ga (**6A/B-Ga**, t_R_ 23.8 min).

Piscibactin-Ga (**1-Ga**): HR-ESIMS *m/z* 519.9935 [M + H]^+^ (calculated for C_19_H_21_N_3_O_4_S_3_Ga^+^, 519.9944); UV λ_max_ 230 nm, 272 nm, 354 nm; ^1^H NMR (500 MHz, CD_3_OD) and ^13^C NMR (125 MHz, CD_3_OD) data (see Table S5).

2-Hydroxypiscibactin-Ga (**7-Ga**): HR-ESIMS *m/z* 535.9887 [M + H]^+^ (calculated for C_19_H_21_N_3_O_5_S_3_Ga^+^, 535.9894); UV λ_max_ 227 nm, 278 nm, 371 nm; ^1^H NMR (500 MHz, CD_3_OD) and ^13^C NMR (125 MHz, CD_3_OD) data (see Table S5).

Photoxenobactin E-Ga (**6A/B-Ga**): HR-ESIMS *m/z* 535.9720 [M + H]^+^ (calculated for C_19_H_21_N_3_O_3_S_4_Ga^+^, 535.9722); UV λ_max_ 233 nm, 265 nm, 359 nm, 398 nm; ^1^H NMR (500 MHz, DMSO-*d_6_*) and ^13^C NMR (125 MHz, DMSO-*d_6_*) data (see Table S6).

2-Hydroxyphotoxenobactin E-Ga (**8A/B-Ga**): HR-ESIMS *m/z* 551.9665 [M + H]^+^ (calculated for C_19_H_21_N_3_O_4_S_4_Ga^+^, 551.9671), UV λ_max_ 228 nm, 276 nm, 377 nm; ^1^H NMR (500 MHz, CDCl_3_) and ^13^C NMR (125 MHz, CDCl_3_) data (see Table S6).

### Biological activity of piscibactin and derivatives

*V. anguillarum* siderophore non-producing strain Δ*vabD* and its derivative mutants Δ*frpA*, Δ*fvtA*, and Δ*frpA*Δ*fvtA* were grown at 25°C overnight. The OD_600_ of each culture was adjusted to 0.5, and a 1:40 dilution was inoculated in CM9 medium supplemented with 75 or 125 µM 2,2’-dipyridyl, using a 96-well microtiter plate with a final volume of 200 µL. Pcb/PxbE forms were added at the final concentration of 1 and 10 µM from a stock solution prepared with MeOH:MQ water (1:1). Cultures supplemented with 10 µM FeCl_3_ and 75 or 125 µM 2,2’-dipyridyl were used as controls. The plate was incubated at 15°C, shaking at 120 rpm for 48 h, and final growth was measured in an iMACK Microplate reader (Bio-Rad). The assay corresponding to each strain was performed in duplicate per plate, and the results shown are the mean values of three independent experiments.

### Phylogenetic reconstruction of *frpA* (*irp*-HPI) and *entD* genes and analysis of their genomic context

The sequences of *frpA* homologs were utilized to infer the diversity and phylogeny of the *irp*-HPI islands. BLASTn searches were conducted in the whole-genome shotgun (wgs) NCBI database using the *frpA* nucleotide sequence of *V. anguillarum* RV22 as a query. A sequence from each group of *frpA* homologs (≥99% identity) was downloaded. Subsequently, a Blast search was conducted in the whole-genome sequence of these *irp*-HPI carrier strains to determine the location of the *entD* gene(s). Following this, the genomic contexts of each *irp*-HPI island and their corresponding *entD* genes were manually inspected to determine whether they are closely associated or located in different regions of the genome. The alignments of respective *frpA* and *entD* sequences were carried out using MUSCLE (66). The phylogenetic trees were constructed by using the maximum likelihood method and general time reversible model using MEGA 11 software (67). These analyses involved 28 nucleotide sequences of each gene. Initial trees for the heuristic search were obtained automatically by applying Neighbor-Joining and BioNJ algorithms to a matrix of pairwise distances estimated using the maximum composite likelihood (MCL) approach and then selecting the topology with superior log likelihood value. A discrete Gamma distribution was employed to model evolutionary rate differences among sites, with five categories (+G). The parameter values were 1.4209 for *frpA* and 0.8528 for *entD*. Codon positions included were 1st + 2nd +3rd. All positions with less than 50% site coverage were eliminated from the data set (partial deletion option). The final data set of *frpA* comprised 1,983 nucleotide positions, while the data set of *entD* comprised 708 positions.

### Statistical analysis

Details on sample sizes, reproducibility, and statistical tests used are provided in the figure legends. Unless otherwise specified, data correspond to *n* = 6 independent biological replicates conducted on separate days. Feature-based molecular networking analyses include *n* = 3 technical replicates. Different biological replicates were analyzed independently on different days to confirm the data.

## SUPPLEMENTAL MATERIAL

Document S1 mbio.01412-25-s0001.pdf Fig. S1-S34 and Tables S1-S6.

ASM does not own the copyrights to Supplemental Material that may be linked to, or accessed through, an article. The authors have granted ASM a non-exclusive, world-wide license to publish the Supplemental Material files. Please contact the corresponding author directly for reuse.
