# Supplementary material for: Siderophore systems at play: system interactions as drivers of diversification in pathogenic Vibrio
Source: mBio. 2025 Aug 18;16(9):e01412-25. doi: 10.1128/mbio.01412-25 (PMC12421891; doi:10.1128/mbio.01412-25)
Supplement: Document S1 — Fig. S1-S34 and Tables S1-S6. [file mbio.01412-25-s0001.pdf]

Siderophore systems at play: systems interactions as drivers of diversification in pathogenic *Vibrio*

Marta. A. Lages,[1,‡] Lucía Ageitos,[2,‡] Larissa Buedenbender, [2,‡] Jaime Rodríguez,[2*] Manuel. L. Lemos,[1*] Carlos Jiménez,[2*] and Miguel Balado[1,*]

[1]Department of Microbiology and Parasitology, Aquatic One Health Research Center (iARCUS), Universidade de Santiago de Compostela, Santiago de Compostela, Spain.

[2]Department of Chemistry, Faculty of Sciences, CICA - Interdisciplinary Center for Chemistry and Biology, Universidade da Coruña, A Coruña, Spain.

‡ These authors contributed equally to this work

*Corresponding author

Index

1. Bacterial strains, plasmids and oligonucleotides 5

Table S1: Strains and plasmids used in this study...5

Table S2: Primers used in this study. Restriction sites are underlined. 6

2. Chemistry Methods 7

2.1 General information and procedures. 7

Table S3: MS and DDA acquisition parameters for *V. anguillarum* RV22 H3 and H4 fraction analyses 7

Table S4: Mzmine parameters for *V. anguillarum* RV22 H3 and H4 fraction analyses 8

3. Molecular networking analysis 9

Figure S1: Global molecular network from *V. anguillarum* RV22. Highlighted in green are those networks or single nodes related to Pcb, while in pink are those related to vanchrobactin. 9

4. Characterization of piscibactin and analogues 10

Figure S2: (+)-HRESIMS spectrum of Pcb-Ga (1-Ga) isolated from the H3 fraction, eluted at 1:1 MeCN:H_2O, from the mutant strain *V. anguillarum* RV22 Δ*vabF*...10

Figure S3: ^{1}H NMR spectrum (CD_3OD, 500 MHz) of Pcb-Ga (1-Ga) isolated from the H3 fraction, eluted with 1:1 MeCN:H_2O, from the mutant strain *V. anguillarum* RV22 Δ*vabF*.......................10

Figure S4: ^{13}C NMR spectrum (CD_3OD, 126 MHz) of Pcb-Ga (1-Ga) isolated from the H3 fraction, eluted with 1:1 MeCN:H_2O, from the mutant strain *V. anguillarum* RV22 Δ*vabF*.......................11

Figure S5: (+)-HRESIMS spectrum of PxbE-Ga (6-Ga) isolated from the H3 fraction, eluted with 1:1 MeCN:H_2O, from the mutant strain *V. anguillarum* RV22 Δ*vabF*...11

Figure S6: ^{1}H NMR spectrum (DMSO-D_6, 500 MHz) of PxbE-Ga (6-Ga) isolated from the H3 fraction, eluted with 1:1 MeCN:H_2O, from the mutant strain *V. anguillarum* RV22 Δ*vabF*.......................12

Figure S7: ^{13}C NMR spectrum (DMSO-D_6, 126 MHz) of PxbE-Ga (6-Ga) isolated from the H3 fraction, eluted with 1:1 MeCN:H_2O, from the mutant strain *V. anguillarum* RV22 Δ*vabF*. 12

Figure S8: ^{1}H-^{1}H COSY spectrum (DMSO-D_6, 500 MHz) of PxbE-Ga (6-Ga) isolated from the H3 fraction, eluted at 1:1 MeCN:H_2O, from the mutant strain *V. anguillarum* RV22 Δ*vabF*.............13

Figure S9: ^{1}H- ^{13}C HSQC NMR spectrum (DMSO-D_6, 126 MHz) of PxbE-Ga (6-Ga) isolated from the H3 fraction, eluted with 1:1 MeCN:H_2O, from the mutant strain *V. anguillarum* RV22 Δ*vabF*. 13

Figure S10: ^{1}H – ^{13}C HMBC NMR spectrum (DMSO-D_6, 126 MHz) of PxbE-Ga (6-Ga) isolated from the H3 fraction, eluted with 1:1 MeCN:H_2O, from the mutant strain *V. anguillarum* RV22 Δ*vabF*. 14

Figure S11: (+)-HRESIMS spectrum of 2-OH-Pcb-Ga (7-Ga) isolated from the H3 fraction, eluted with 1:1 MeCN:H_2O, from the mutant strain *V. anguillarum* RV22 Δ*vabF*. 14

Figure S12: ^{1}H NMR spectrum (CD_3OD, 126 MHz) of 2-OH-Pcb-Ga (7-Ga) isolated from the H3 fraction, eluted with 1:1 MeCN:H_2O, from the mutant strain *V. anguillarum* RV22 Δ*vabF*. 15

Figure S13: ^{13}C NMR spectrum (CD_3OD, 125 MHz) of 2-OH-Pcb-Ga (7-Ga) isolated from the H3 fraction, eluted with 1:1 MeCN:H_2O, from the mutant strain *V. anguillarum* RV22 Δ*vabF*. 15

Figure S14: ^{1}H – ^{1}H COSY NMR spectrum (CD_3OD, 500 MHz) of 2-OH-Pcb-Ga (7-Ga) isolated from the H3 fraction, eluted with 1:1 MeCN:H_2O, from the mutant strain *V. anguillarum* RV22 Δ*vabF*. 16

Figure S15: ^{1}H – ^{13}C HSQC NMR spectrum (MeOD, 500 MHz) of 2-OH-Pcb-Ga (7-Ga) isolated from the H3 fraction, eluted with 1:1 MeCN:H_2O, from the mutant strain *V. anguillarum* RV22 Δ*vabF*. 16

Figure S16: ^{1}H – ^{13}C HMBC NMR spectrum (MeOD, 500 MHz) of 2-OH-Pcb-Ga (7-Ga) isolated from the H3 fraction, eluted with 1:1 MeCN:H_2O, from the mutant strain *V. anguillarum* RV22 Δ*vabF*. 17

Figure S17: (+)-HRESIMS spectrum of 2-OH-PxbE-Ga (8-Ga) isolated from the H3 fraction, eluted with 1:1 MeCN:H_2O, from the mutant strain *V. anguillarum* RV22 Δ*vabF*. 17

Figure S18: ^{1}H NMR spectrum ($CDCl_3$, 500 MHz) of 2-OH-PxbE-Ga (8-Ga) isolated from the H3 fraction, eluted with 1:1 MeCN:H_2O, from the mutant strain *V. anguillarum* RV22 Δ*vabF*. 18

Figure S19: ^{13}C NMR spectrum ($CDCl_3$, 125 MHz) of 2-OH-PxbE-Ga (8-Ga) isolated from the H3 fraction, eluted with 1:1 MeCN:H_2O, from the mutant strain *V. anguillarum* RV22 Δ*vabF*. 18

Figure S20: ^{1}H – ^{1}H COSY NMR spectrum ($CDCl_3$, 500 MHz) of 2-OH-PxbE-Ga (8-Ga) isolated from the H3 fraction, eluted with 1:1 MeCN:H_2O, from the mutant strain *V. anguillarum* RV22 Δ*vabF*. 19

Figure S21: ^{1}H - ^{13}C HSQC NMR spectrum ($CDCl_3$, 500 MHz) of 2-OH-PxbE-Ga (8-Ga) isolated from the H3 fraction, eluted with 1:1 MeCN:H_2O, from the mutant strain *V. anguillarum* RV22 Δ*vabF*. 19

Figure S22: ^{1}H – ^{13}C HMBC NMR spectrum ($CDCl_3$, 500 MHz) of 2-OH-PxbE-Ga (8-Ga) isolated from the H3 fraction, eluted with 1:1 MeCN:H_2O, from the mutant strain *V. anguillarum* RV22 Δ*vabF*. 20

Table S5: ^{1}H and ^{13}C NMR data (500/125 MHz) assignments of Pcb-Ga (3-Ga, CD_3OD), and 2-OH-Pcb-Ga (CD_3OD). 21

Table S6: ^{1}H and ^{13}C NMR data (500/125 MHz) assignments of PxbE-Ga (6-Ga, CD_3SOCD_3), and 2-OH-PxbE-Ga (8-Ga, $CDCl_3$). 22

5. MS/MS fragmentation pattern of Pcb/Pxb catechol analogues 23

Figure S23: MS/MS fragmentation pattern of: A) 2-OH-Pcb-Fe (7-Fe) and B) 2-OH-PxbE-Fe (8-Fe) 23

6. Mutant profiling extracted ion chromatograms 24

Figure S24: Total Ion Chromatogram (TIC) of the H3 fraction chelated with iron from the wild-type strain *V. anguillarum* RV22 on the top, followed by the extracted ion chromatograms of the different siderophores reported within this work (Pcb-Fe (1-Fe), Pre-Pcb (2), Vcb (3), PxbB-Fe (4-Fe), PxbD-Fe (5-Fe), PxbE-Fe (6-Fe), 2-OH-Pcb-Fe (7-Fe), 2-OH-PxbE-Fe (8-Fe)). 24

Figure S25: Total Ion Chromatogram (TIC) of the H3 fraction chelated with iron from the mutant strain *V. anguillarum* RV22 Δ*vabF* on the top, followed by the extracted ion chromatograms of the different siderophores reported within this work (Pcb-Fe (1-Fe), Pre-Pcb (2), Vcb (3), PxbB-Fe (4-Fe), PxbD-Fe (5-Fe), PxbE-Fe (6-Fe), 2-OH-Pcb-Fe (7-Fe), 2-OH-PxbE-Fe (8-Fe)). 25

Figure S26: Total Ion Chromatogram (TIC) of the H3 fraction chelated with iron from the mutant strain *V. anguillarum* RV22 Δ*vabB* on the top, followed by the extracted ion chromatograms of the different siderophores reported within this work (Pcb-Fe (1-Fe), Pre-Pcb (2), Vcb (3), PxbB-Fe (4-Fe), PxbD-Fe (5-Fe), PxbE-Fe (6-Fe), 2-OH-Pcb-Fe (7-Fe), 2-OH-PxbE-Fe (8-Fe)). 26

Figure S27: Total Ion Chromatogram (TIC) of the H3 fraction chelated with iron from the mutant strain *V. anguillarum* RV22 Δ*vabE* on the top, followed by extracted ion chromatograms of the different siderophores reported within this work (Pcb-Fe (1-Fe), Pre-Pcb (2), Vcb (3), PxbB-Fe (4-Fe), PxbD-Fe (5-Fe), PxbE-Fe (6-Fe), 2-OH-Pcb-Fe (7-Fe), 2-OH-PxbE-Fe (8-Fe)). 27

Figure S28: Total Ion Chromatogram (TIC) of the H3 fraction chelated with iron from the mutant strain *V. anguillarum* RV22 Δ*vabF*Δ*irp5* on the top, followed by the extracted ion chromatograms of the different siderophores reported within this work (Pcb-Fe (1-Fe), Pre-Pcb (2), Vcb (3), PxbB-Fe (4-Fe), PxbD-Fe (5-Fe), PxbE-Fe (6-Fe), 2-OH-Pcb-Fe (7-Fe), 2-OH-PxbE-Fe (8-Fe)). 28

Figure S29: Total Ion Chromatogram (TIC) of the H3 fraction chelated with iron from the mutant strain *V. anguillarum* RV22 Δ*vabF*Δ*irp9* on the top, followed by the extracted ion chromatograms of the different siderophores reported within this work (Pcb-Fe (1-Fe), Pre-Pcb (2), Vcb (3), PxbB-Fe (4-Fe), PxbD-Fe (5-Fe), PxbE-Fe (6-Fe), 2-OH-Pcb-Fe (7-Fe), 2-OH-PxbE-Fe (8-Fe)). 29

Figure S30: Total Ion Chromatogram (TIC) of the H3 fraction chelated with iron from the mutant strain *V. anguillarum* RV22 Δ*vabF*Δ*TE-I* on the top, followed by the extracted ion chromatograms of the different siderophores reported within this work (Pcb-Fe (1-Fe), Pre-Pcb (2), Vcb (3), PxbB-Fe (4-Fe), PxbD-Fe (5-Fe), PxbE-Fe (6-Fe), 2-OH-Pcb-Fe (7-Fe), 2-OH-PxbE-Fe (8-Fe)). 30

Figure S31: Total Ion Chromatogram (TIC) of the H3 fraction chelated with iron from the mutant strain *V. anguillarum* RV22 Δ*vabF*Δ*M6* on the top, followed by the extracted ion chromatograms of the different siderophores reported within this work (Pcb-Fe (1-Fe), Pre-Pcb (2), Vcb (3), PxbB-Fe (4-Fe), PxbD-Fe (5-Fe), PxbE-Fe (6-Fe), 2-OH-Pcb-Fe (7-Fe), 2-OH-PxbE-Fe (8-Fe))..................31

Figure S32: Total Ion Chromatogram (TIC) of the H3 fraction chelated with iron from the mutant strain *V. anguillarum* RV22 Δ*vabF*Δ*irp4* on the top, followed by the extracted ion chromatograms of the different siderophores reported within this work (Pcb-Fe (1-Fe), Pre-Pcb (2), Vcb (3), PxbB-Fe (4-Fe), PxbD-Fe (5-Fe), PxbE-Fe (6-Fe), 2-OH-Pcb-Fe (7-Fe), 2-OH-PxbE-Fe (8-Fe)). 32

7. Precursor-directed biosynthesis (PDB) analyses 33

Figure S33: Precursor directed biosynthesis (PDB) analysis by addition of 4-Fluoro-2-hydroxybenzoic acid (4-F-2-HBA) in the culture of *V. anguillarum* RV22 Δ*vabF*. A) Total ion chromatogram of *V. anguillarum* RV22 ΔvabF H3 fraction followed by the extracted ion chromatogram of the expected new analogues with Pcb and PxbE bearing the introduced in the structure. B) (+)-HRESIMS of the peak eluting at 13.96 min corresponding to Pcb bearing 4-F-2-HBA in its structure. C) (+)-HRESIMS of the peak eluting at 17.61 min corresponding to PxbE bearing 4-F-2-HBA in its structure. 33

Figure S34: Precursor directed biosynthesis (PDB) analysis by addition of 5-Fluoro-2-hydroxybenzoic acid (5-F-2-HBA) to the culture of *V. anguillarum* RV22 Δ*vabF*. A) Total ion chromatogram of *V. anguillarum* RV22 ΔvabF H3 fraction followed by the extracted ion chromatogram of the expected new analogues with Pcb and PxbE bearing the introduced in the structure. B) (+)-HRESIMS of the peak eluting at 13.68 min corresponding to Pcb bearing 5-F-2-HBA in its structure. C) (+)-HRESIMS of the peak eluting at 17.50 min corresponding to PxbE bearing 5-F-2-HBA in its structure. 34

1. Bacterial strains, plasmids and oligonucleotides

Table S1: Strains and plasmids used in this study.

Strain	Relevant characteristics	Source
V. anguillarum		
RV22	Wild-type serotype O2 strain isolated from diseased turbot (Spain)	(37)
MB14	RV22 with in-frame deletion of *vabF* gene	(37)
MB11	RV22 with in-frame deletion of *vabB* gene	(37)
MB3	RV22 with in-frame deletion of *vabE* gene	(37)
MB67	RV22 with in-frame deletion of *vabD* gene	(12)
ML1261	RV22 with in-frame deletion of *vabF* and *irp5* genes	This study
ML1332	RV22 with in-frame deletion of *vabF* and *irp9* genes	This study
ML1325	RV22 with in-frame deletion of *vabF* and *TE-I* genes	This study
ML178	RV22 with in-frame deletion of *vabF* and *TE-II* genes	(20)
ML1352	RV22 with in-frame deletion of *vabF, TE-I* and *TE-II* genes	This study
ML1427	RV22 with in-frame deletion of *vabF* and *M6* genes	This study
ML210	RV22 with in-frame deletion of *vabD* and *frpA*	(36)
ML1198	RV22 with in-frame deletion of *vabD* and *fvtA*	This study
ML1237	RV22 with in-frame deletion of *vabD, frpA* and *fvtA*	This study
ML1279	ML1261 revertant strain by the reintroduction of *irp5* wild type gene	This study
ML1344	ML1332 revertant strain by the reintroduction of *irp9* wild type gene	This study
ML1361	ML1325 revertant strain by the reintroduction of *TE-I* wild type gene	This study
ML1494	ML1427 revertant strain by the reintroduction of *M6* wild type gene	This study
E. coli		
DH5α	Cloning strain	Laboratory strain
S17-1-*λpir*	RP4 (Km::Tn7, Tc::Mu-1) *pro-82 λpir recA1 end A1 thiE1 hsdR17 creC510*	Laboratory strain
**Plasmids**		
pWKS30	Low-copy cloning vector	(63)
pNidKan	Suicide vector derived from pCVD442	(37)
pML1247	S17 λpir pCAR109 1-4 *irp5*	This study
pML1295	S17 λpir pCAR109 1-4 *irp9*	This study
pML1320	S17 λpir pCAR109 1-4 *TE-I*	This study
pML144	S17 λpir pCAR109 1-4 *TE-II*	(20)
pML1392	S17 λpir pCAR109 1-4 *M6*	This study
pML187	S17 λpir pCAR109 1-4 *frpA*	(36)
pMB42	S17 λpir pCAR109 1-4 *fvtA*	(65)
pML1275	S17 λpir pCAR109 1-4 *irp5* comp	This study
pML1338	S17 λpir pCAR109 1-4 *irp9* comp	This study
pML136	S17 λpir pCAR109 1-4 *TE-I* comp	This study
pML1461	S17 λpir pCAR109 1-4 *M6* comp	This study

Table S2: Primers used in this study. Restriction sites are underlined.

**Oligonucleotide (5' -> 3')**		**Size (bp)**
irp5 mutant construction		
1_Irp5_F_BamHI	CCGGGATCCGTATCATGCTTACCTGCGCC	1001
2_Irp5_R_PstI	GCGCTGCAGCTGATAACGCATCGCTTGTG	
3_Irp5V2_F_PstI	GCGCTGCAGGCACTCATCTCCAAAGACTC	944
4_Irp5v2_R_XhoI	CGCCTCGAGGAGTTAGTGGCTAAGTTGCC	
irp9 mutant construction		
1_Irp9_F_XbaI	CGCTCTAGAGCCCAACCTCGTGAAATATG	967
2_Irp9_R_BamHI	CCGGGATCCCGCCCATTCATTCTCATGCT	
3_Irp9_F_BamHI	GCGGGATCCCCTCAACGAGAATGGGAAGA	1078
4_Irp9_R_XhoI	GGCCTCGAGAAAGCATCAGTGTAGGCCAG	
TE-I mutant construction		
1_TEI_XbaI_F	GCGTCTAGAACGCCTCAGGCTAAATTGCT	1011
2_TEI_PstI_R	CGGCTGCAGGTGTCCGCCAATCGGATGCA	
3_TEv2_F_PstI	GCGCTGCAGTGGAGTGATTGCTGTCATCC	1000
4_TEv2_R_XhoI	CGCCTCGAGCTGCGTAGTCGGTTGATTGA	
M6 mutant construction		
1_XbaI_Mod6_F	GCCTCTAGATTGGCCGTCAATGTTCTGCA	939
2_XhoI_Mod6_R	CGCCTCGAGTTGTGGTTCATCCCATGAGC	
3_XhoI_Mod6_F	CGGCTCGAGCACGAGATCTCTACTCTGCT	925
4_KpnI_Mod6_R	GCGGGTACCGTCGTAAAGTGTCTGCGTAG	
irp5 complementation		
1_Irp5_F_NotI	CCGGCGGCCGCGTATCATGCTTACCTGCGCC	3307
4_Irp5v2_R_ApaI	CGCGGGCCCGAGTTAGTGGCTAAGTTGCC	
irp9 complementation		
Irp9_comp_F_NotI	CGCGCGGCCGCGCCCAACCTCGTGAAATATG	3185
Irp9_comp_R_ApaI	GCCGGGCCCAAAGCATCAGTGTAGGCCAG	
TE-I complementation		
TEI_comp_F_NotI	CGCGCGGCCGCACGCCTCAGGCTAAATTGCT	2638
TEI_comp_R_ApaI	CCGGGGCCCCTGCGTAGTCGGTTGATTGA	
M6 complementation		
1_NotI_Mod6_F	CGCGCGGCCGCTTGGCCGTCAATGTTCTGCA	5527
2_ApaI_Mod6_R	GCGGGGCCCGTCGTAAAGTGTCTGCGTAG	

2. Chemistry Methods

2.1 General information and procedures.

The solvents used in the HPLC-HRMS, and HPLC analyses were LC/MS or LC grade, respectively. MeCN employed for the fractionation was LC analysis grade (Fisher®). Deionized water (MQ-H_2O) was obtained from a Direct-Q ® system (Merck). ^{1}H, ^{13}C, and 2D NMR spectra were recorded on a Bruker Avance 500 (500 MHz for ^{1}H and 125 MHz for ^{13}C) with a dual cryoprobe or a BBI probe. CD_3OD, $CDCl_3$, and DMSO-d_6 were used as deuterated solvents. Chemical shifts are reported in δ _scale relative to CD_3OD (δ _3.31 ppm for ^{1}H NMR, δ _49.0 ppm for ^{13}C NMR), DMSO-d6 (δ _2.50 ppm for ^{1}H NMR, δ _39.52 ppm for ^{13}C NMR), and $CDCl_3$ (δ _7.26 ppm for ^{1}H NMR, δ _77.16 ppm for ^{13}C NMR). Multiplicities of ^{13}C signals were obtained by DEPT. HPLC separation was performed on an Agilent 1100 and 1200 equipped with a solvent degasser, quaternary pump and an UV detector (Agilent Technologies, Waldbronn, Germany) using an Atlantis dC18 (100 x 4.6 mm, 5 µm, Waters) column. HRMS, HPLC-HRMS, and HPLC-MS/MS data were acquired on a LTQ-Orbitrap Discovery mass spectrometer coupled to an Accela HPLC (Thermo Scientific). OASIS® HLB cartridge (35 cc, 6 g) were used for SPE fractionation.

Table S3: MS and DDA acquisition parameters for *V. anguillarum* RV22 H3 and H4 fraction analyses

**Parameter**	**Value**
**MS parameters**	
Spray voltage	3.90 kV
Capillary temperature	350 °C
Sheat gas	50 units N_2 (ca. 500 mL min^{-1})
Auxiliary gas rate	10 units N_2 (ca. 100 mL min^{-1})
Noise Level	5E4
**Data Dependent Acquisition (DDA)**	Five topmost intense ions
Maximum ion injection time MS^1	100 ms
Maximum ion injection time MS^2	100 ms
CID isolation width	3
CID normalized collision energy	35.0 eV
Activation Q	0.250
Activation time	30 ms
**Dynamic exclusion**	3
Repeat count	5
Repeat duration	10 s
Exclusion duration	20 s
Exclusion mass width	-0.01 to +2.1

Table S4: Mzmine parameters for *V. anguillarum* RV22 H3 and H4 fraction analyses

Parameter	Value
**Mass detection**	
RT range	2-45 min
MS Level	1
Mass detector	Centroid
Noise Level	5E4
**Mass detection**	
RT range	2-40min
MS Level	2
Mass detector	Centroid
Noise Level	500
**ADAP Chromatogram builder**	
MS Level	1
Min. group size in # of scans	3
Group intensity threshold	5E4
Min highest intensity	5E4
m/z tolerance	0.005 *m/z* or 10 ppm
**Chromatogram resolving:** Local minimum resolver	
Chromatographic threshold	90%
search minimum RT range	0.05 min
minimum rel. Height	0
minimum absol. Height	6E4
ratio top/edge	1.9
peak duration	0.01 - 2 min
Min # data points	3
m/z range for MS2 scan pairing (Da)	0.5
RT tolerance for MS2 scan pairing (min)	0.05
**Join aligner**	
**Deisotoping**	
m/z tolerance	0.005 *m/z* or 5 ppm
weight for m/z	70
retention time tolerance	0.5 min
weight for retention time	30
**Feature list filtering**	
Isotope tolerance	0.005/5ppm; 1E4, 80%
**Feature list blank filtering**	Min # detection 2
**Peak list rows filter:** Feature with MS2 scan (GNPS)	Yes
**Peak list rows filter:** Min features in row	2
**Duplicate feature filter**	0.0005/5ppm; 2.0min, new average
Removed known system contaminants	*m/z* 359; 254, 288
**Export for/Submit to GNPS**	

3. Molecular networking analysis

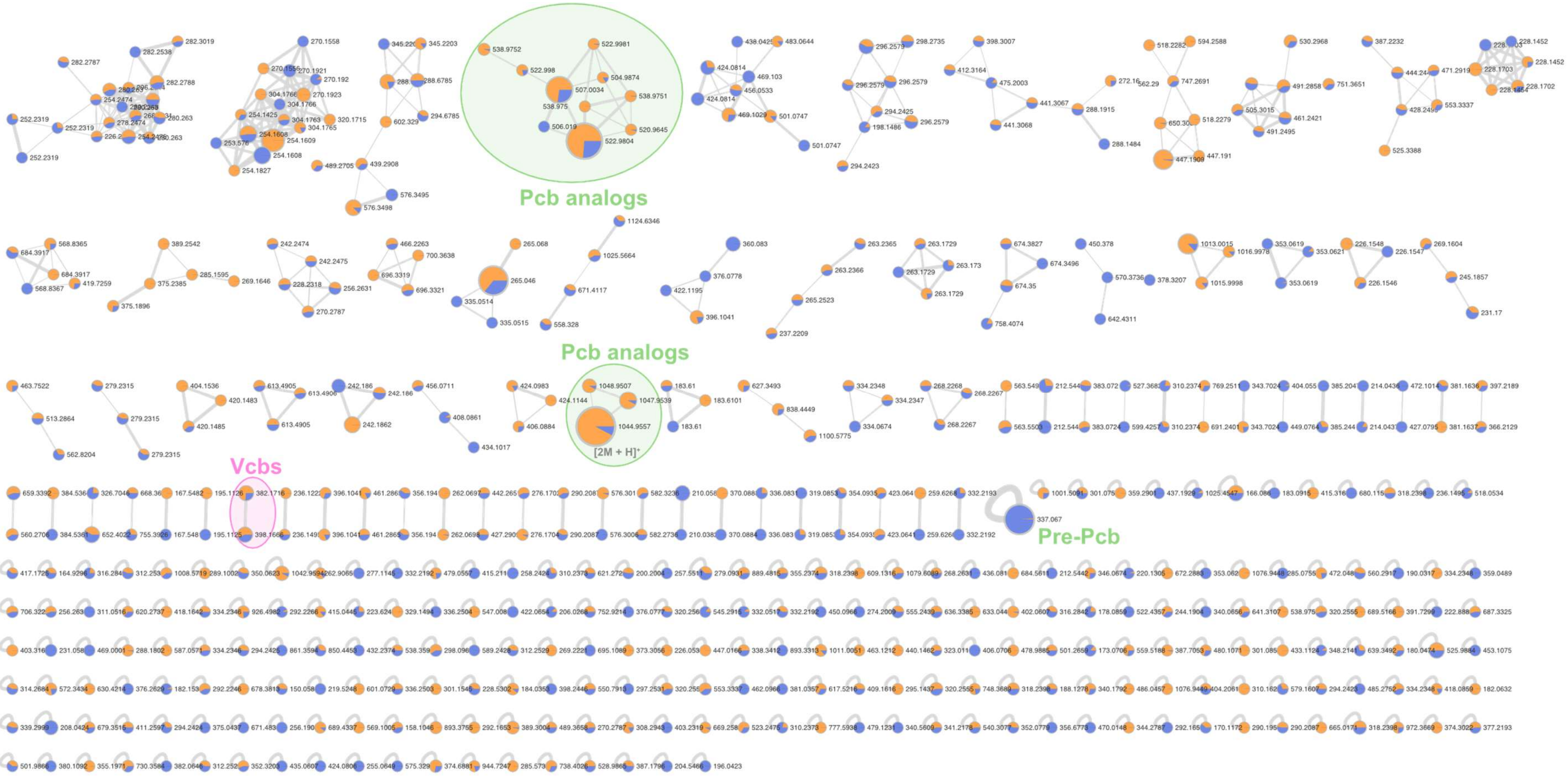

Figure S1: Global molecular network from *V. anguillarum* RV22. Highlighted in green are those networks or single nodes related to Pcb, while in pink are those related to vanchrobactin.

4. Characterization of piscibactin and analogues

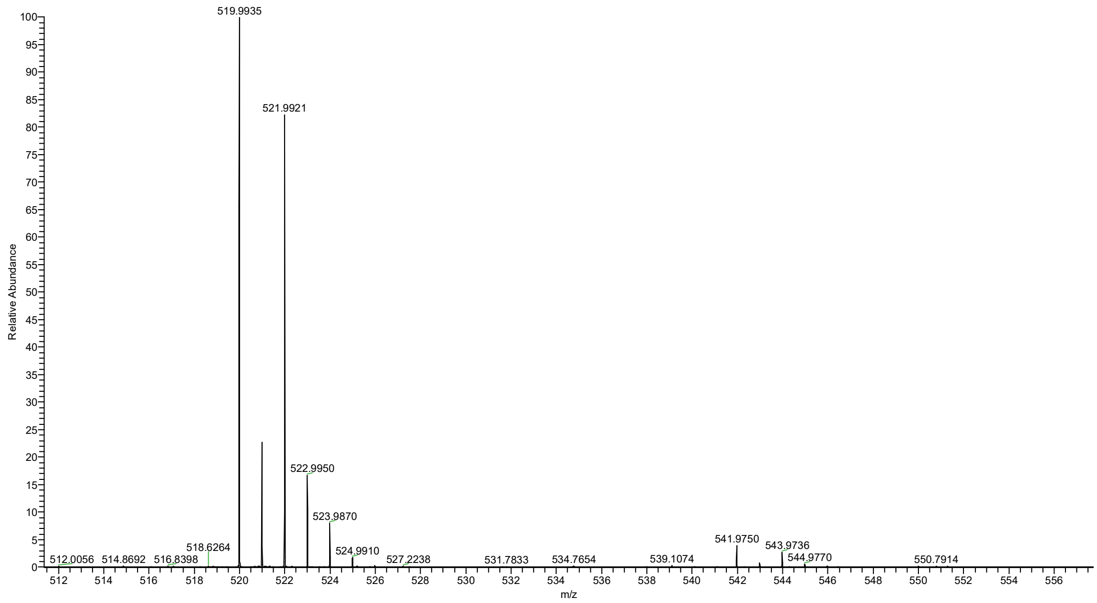

Figure S2: (+)-HRESIMS spectrum of Pcb-Ga (**1-Ga**) isolated from the H3 fraction, eluted at 1:1 MeCN:H_2O, from the mutant strain *V. anguillarum* RV22 Δ*vabF*.

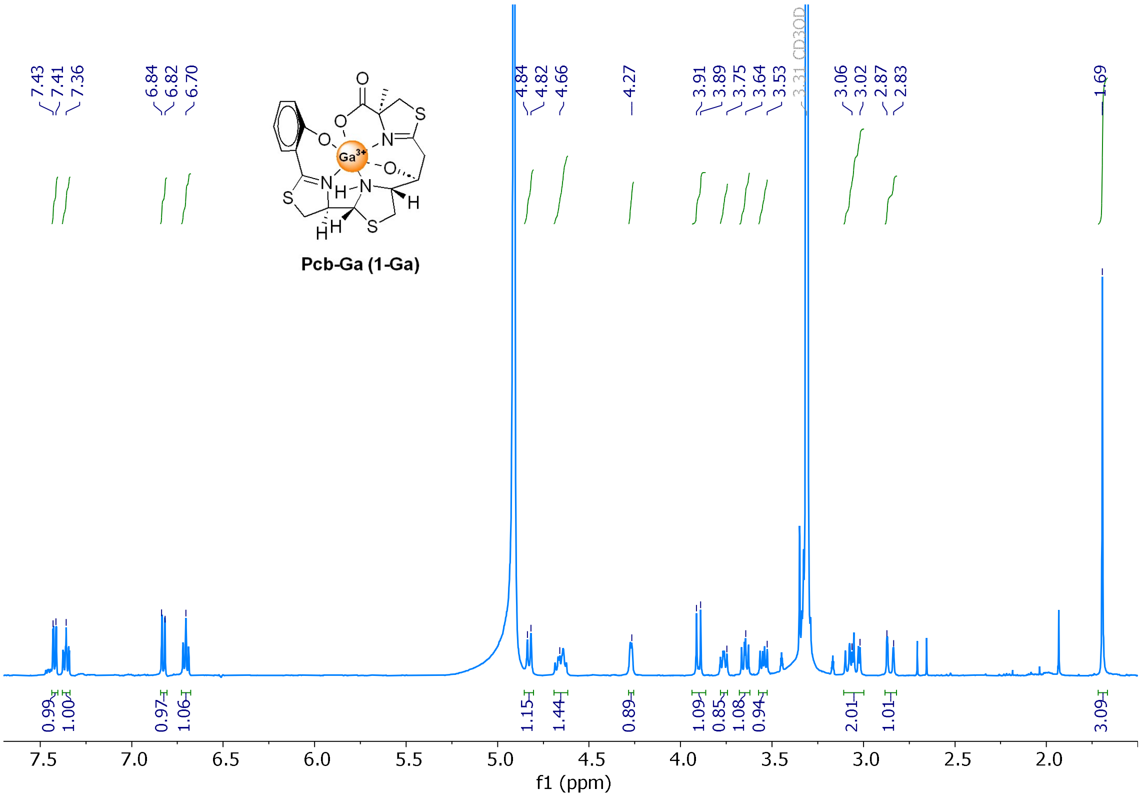

Figure S3: ^{1}H NMR spectrum (CD_3OD, 500 MHz) of Pcb-Ga (**1-Ga**) isolated from the H3 fraction, eluted with 1:1 MeCN:H_2O, from the mutant strain *V. anguillarum* RV22 Δ*vabF*.

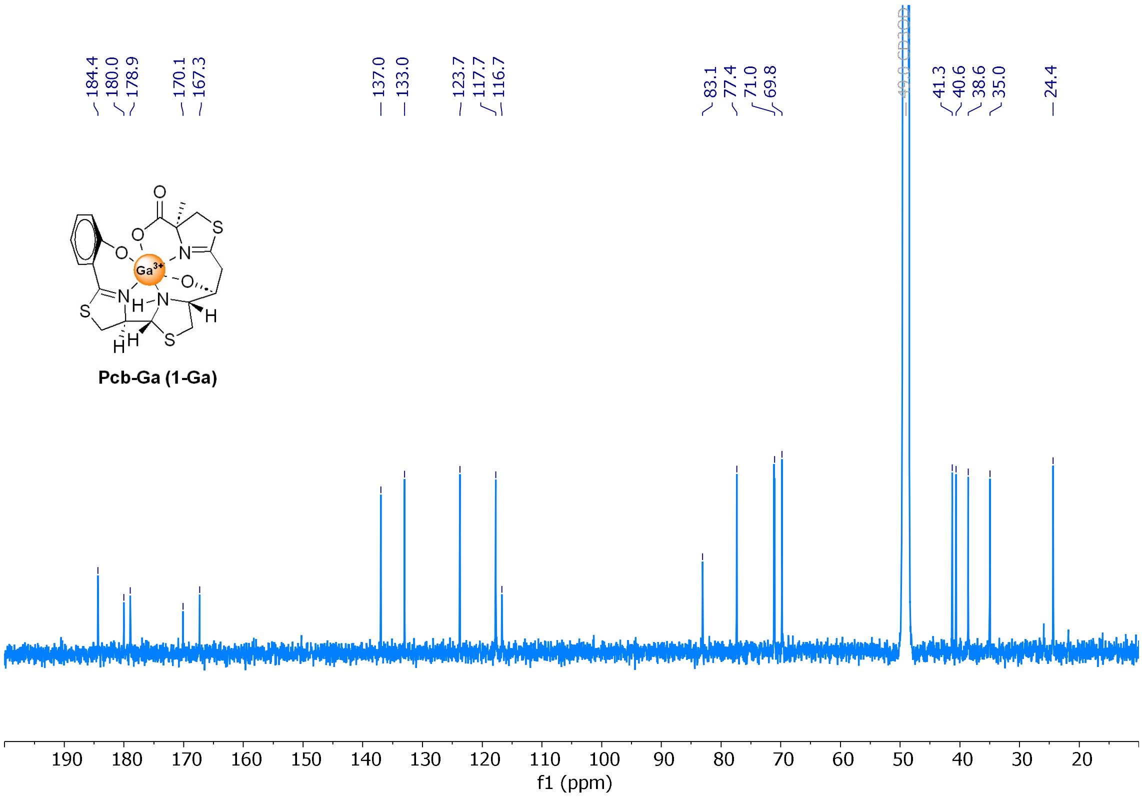

Figure S4: ^{13}C NMR spectrum (CD_3OD, 126 MHz) of Pcb-Ga (**1-Ga**) isolated from the H3 fraction, eluted with 1:1 MeCN:H_2O, from the mutant strain *V. anguillarum* RV22 Δ*vabF*.

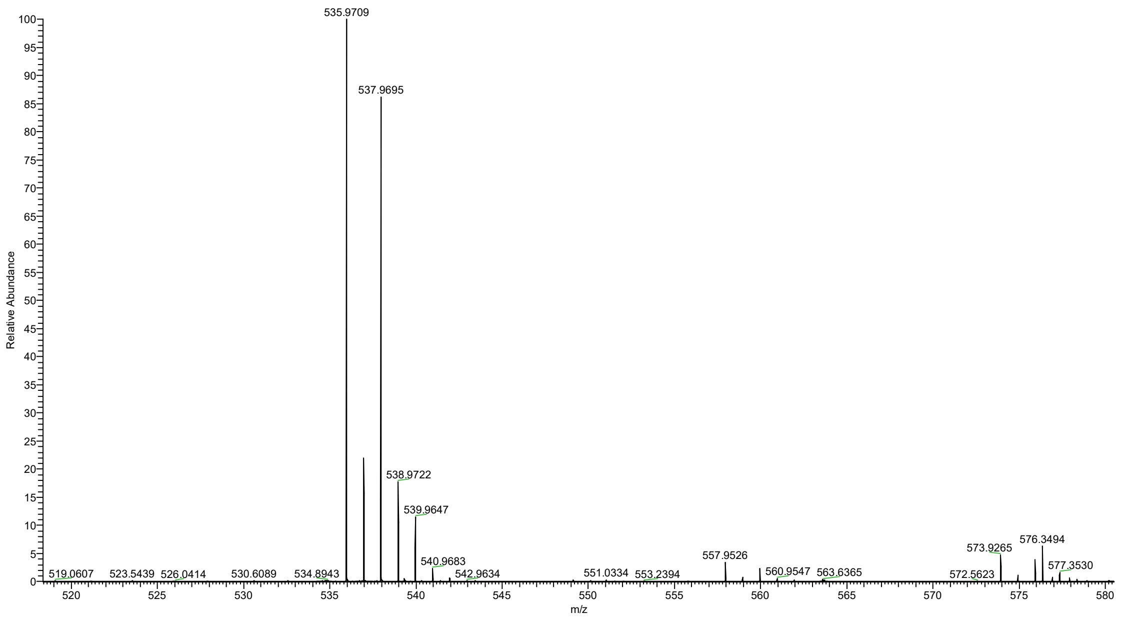

Figure S5: (+)-HRESIMS spectrum of PxbE-Ga (**6-Ga**) isolated from the H3 fraction, eluted with 1:1 MeCN:H_2O, from the mutant strain *V. anguillarum* RV22 Δ*vabF*.

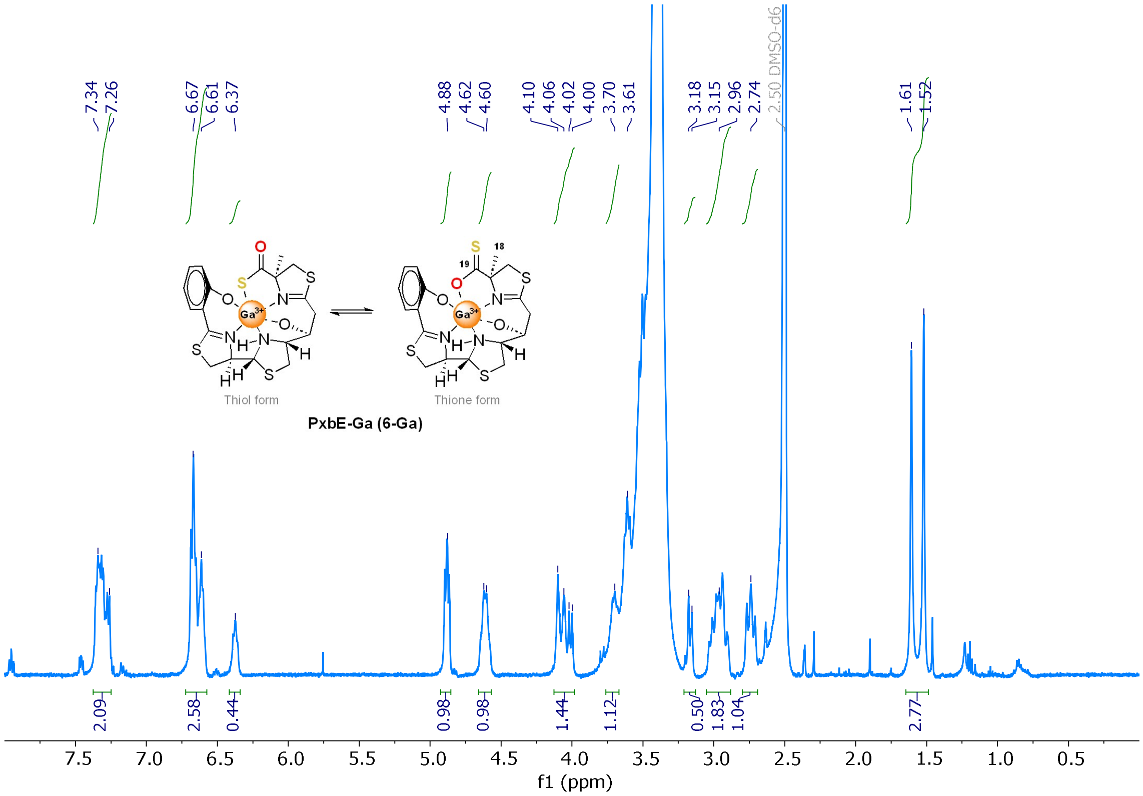

Figure S6: ^{1}H NMR spectrum (DMSO-D$_6$, 500 MHz) of PxbE-Ga (**6-Ga**) isolated from the H3 fraction, eluted with 1:1 MeCN:H_2O, from the mutant strain *V. anguillarum* RV22 Δ*vabF*.

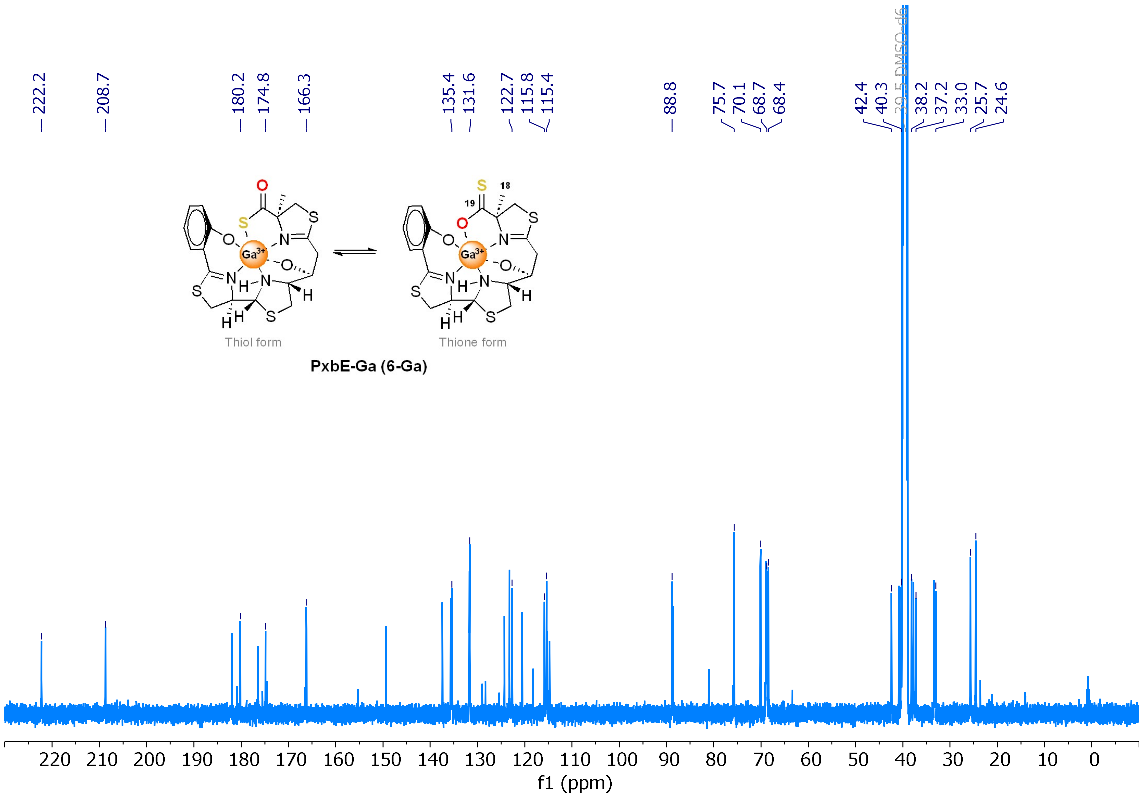

Figure S7: ^{13}C NMR spectrum (DMSO-D$_6$, 126 MHz) of PxbE-Ga (**6-Ga**) isolated from the H3 fraction, eluted with 1:1 MeCN:H_2O, from the mutant strain *V. anguillarum* RV22 Δ*vabF*.

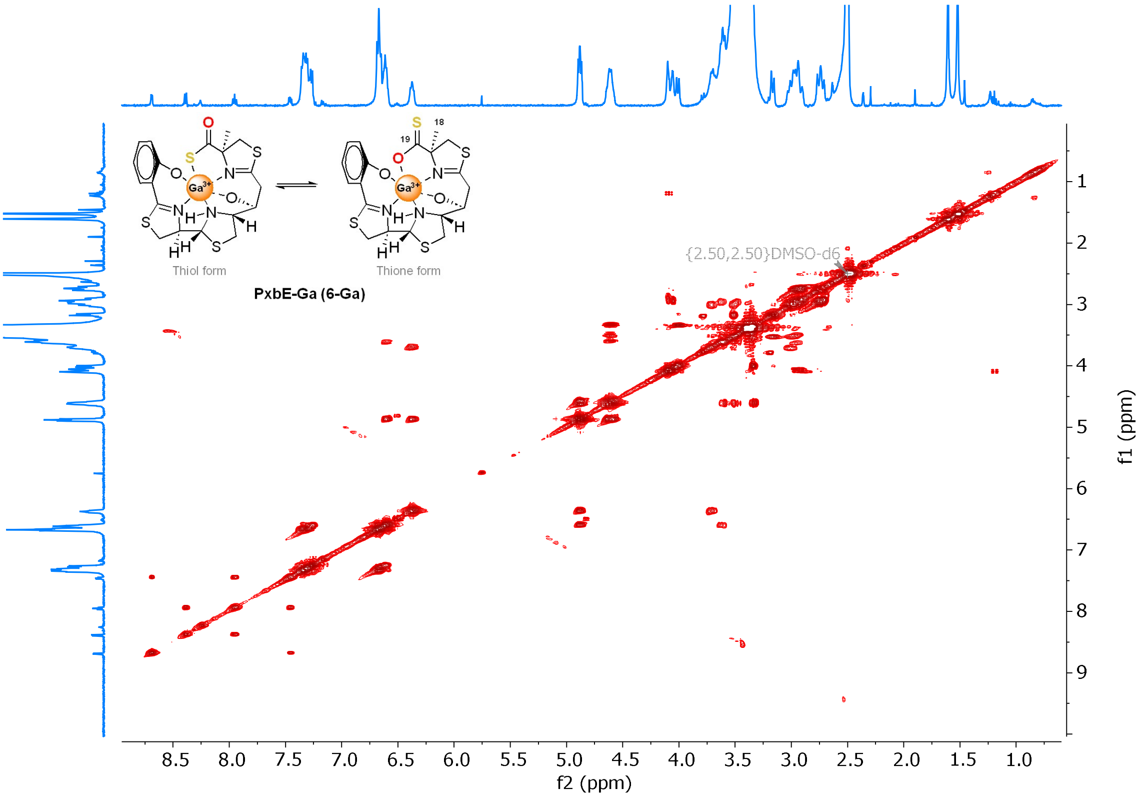

Figure S8: ^{1}H-^{1}H COSY spectrum (DMSO-D$_6$, 500 MHz) of PxbE-Ga (**6-Ga**) isolated from the H3 fraction, eluted at 1:1 MeCN:H_2O, from the mutant strain *V. anguillarum* RV22 Δ*vabF*.

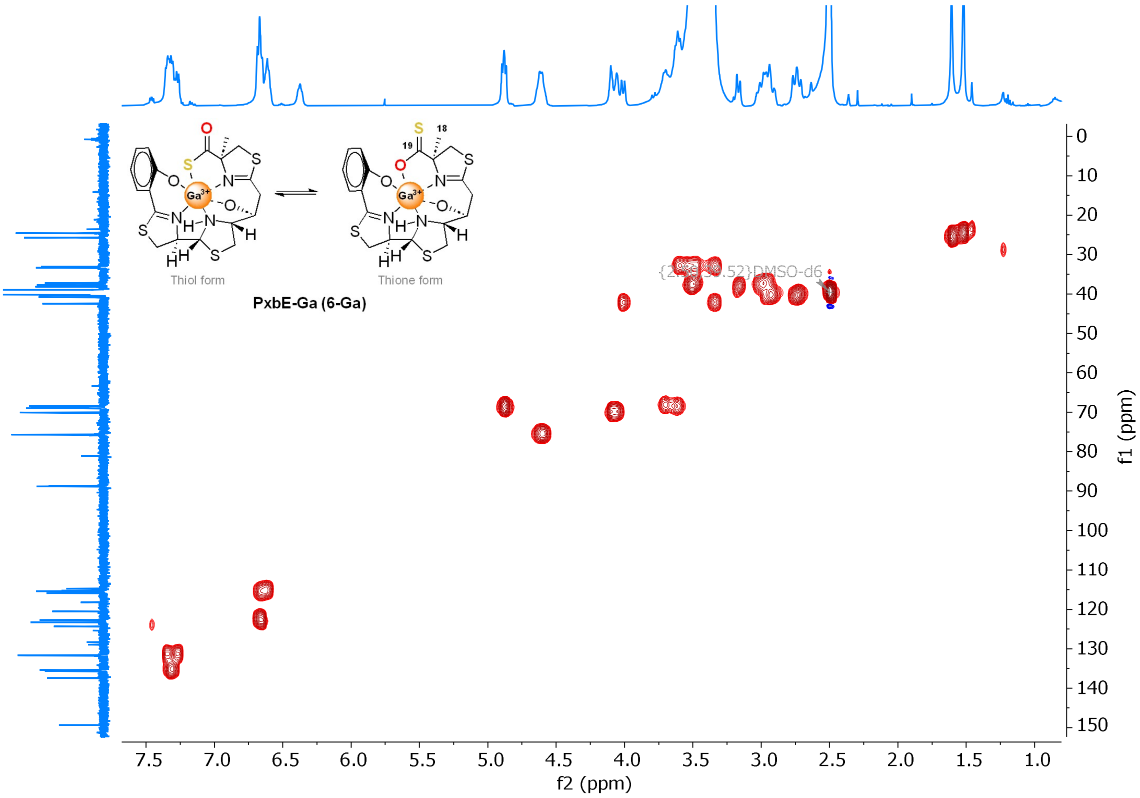

Figure S9: ^{1}H- ^{13}C HSQC NMR spectrum (DMSO-D$_6$, 126 MHz) of PxbE-Ga (**6-Ga**) isolated from the H3 fraction, eluted with 1:1 MeCN:H_2O, from the mutant strain *V. anguillarum* RV22 Δ*vabF*.

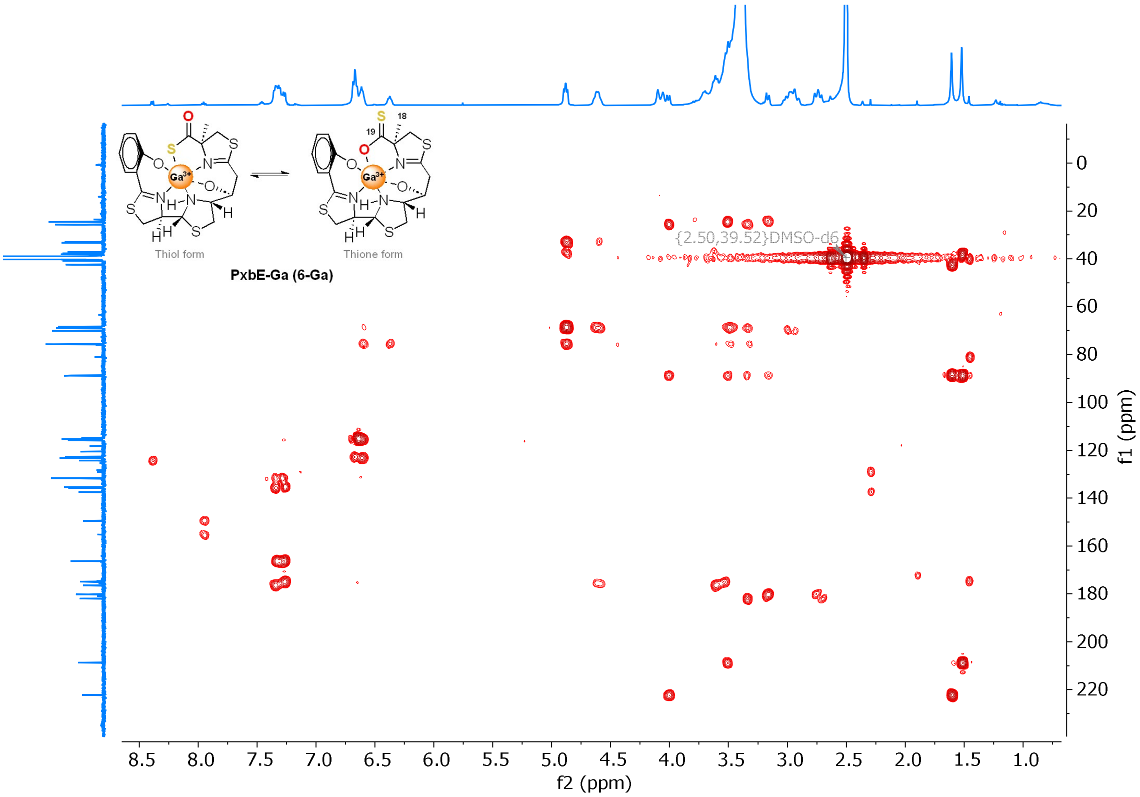

Figure S10: ^{1}H – ^{13}C HMBC NMR spectrum (DMSO-D$_6$, 126 MHz) of PxbE-Ga (**6-Ga**) isolated from the H3 fraction, eluted with 1:1 MeCN:H_2O, from the mutant strain *V. anguillarum* RV22 Δ*vabF*.

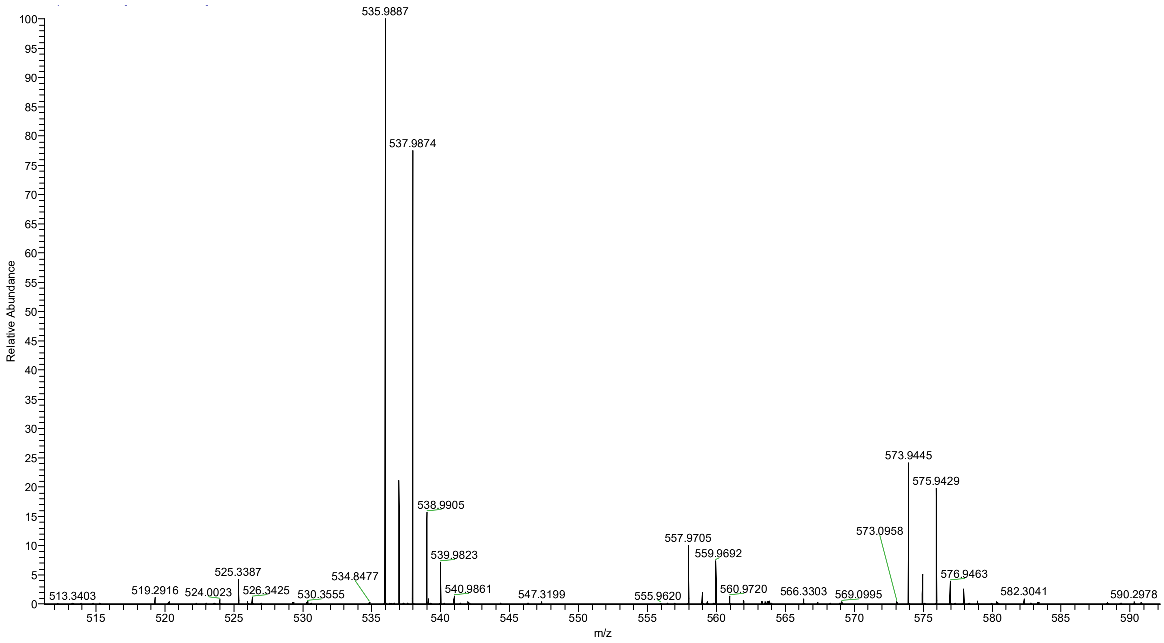

Figure S11: (+)-HRESIMS spectrum of 2-OH-Pcb-Ga (**7-Ga**) isolated from the H3 fraction, eluted with 1:1 MeCN:H_2O, from the mutant strain *V. anguillarum* RV22 Δ*vabF*.

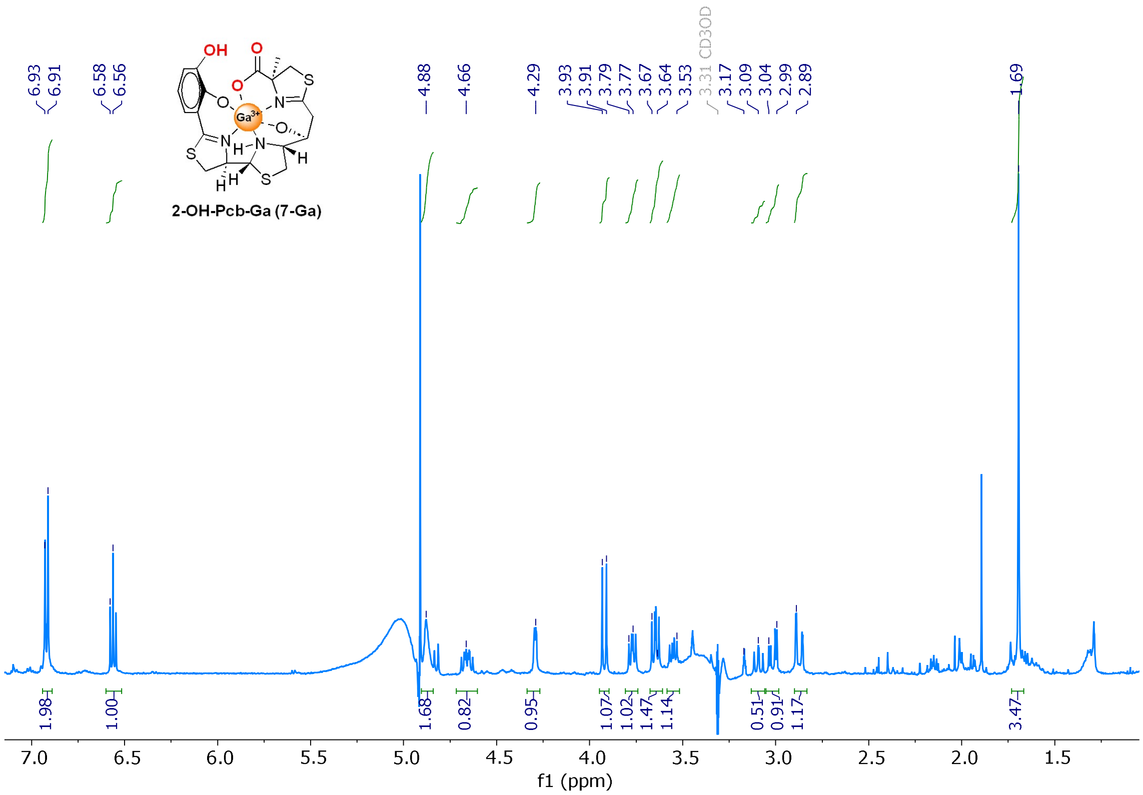

Figure S12: ^{1}H NMR spectrum (CD_3OD, 126 MHz) of 2-OH-Pcb-Ga (**7-Ga**) isolated from the H3 fraction, eluted with 1:1 MeCN:H_2O, from the mutant strain *V. anguillarum* RV22 Δ*vabF*.

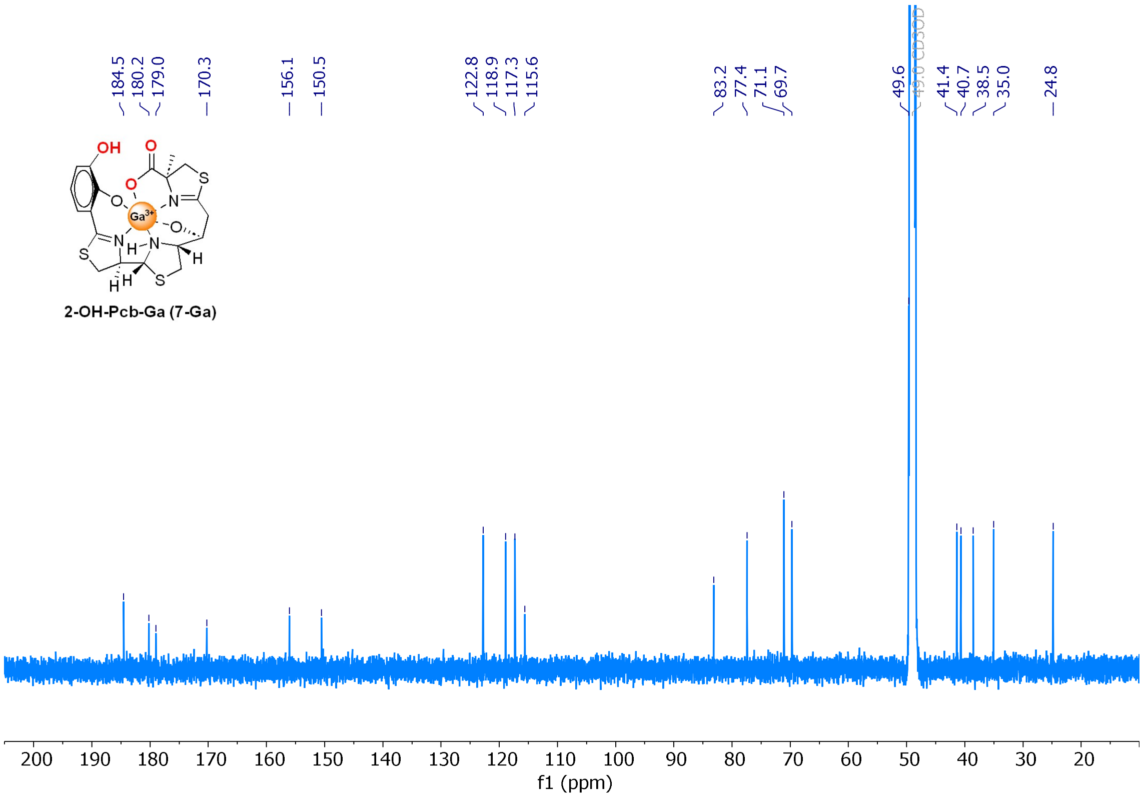

Figure S13: ^{13}C NMR spectrum (CD_3OD, 125 MHz) of 2-OH-Pcb-Ga (**7-Ga**) isolated from the H3 fraction, eluted with 1:1 MeCN:H_2O, from the mutant strain *V. anguillarum* RV22 Δ*vabF*.

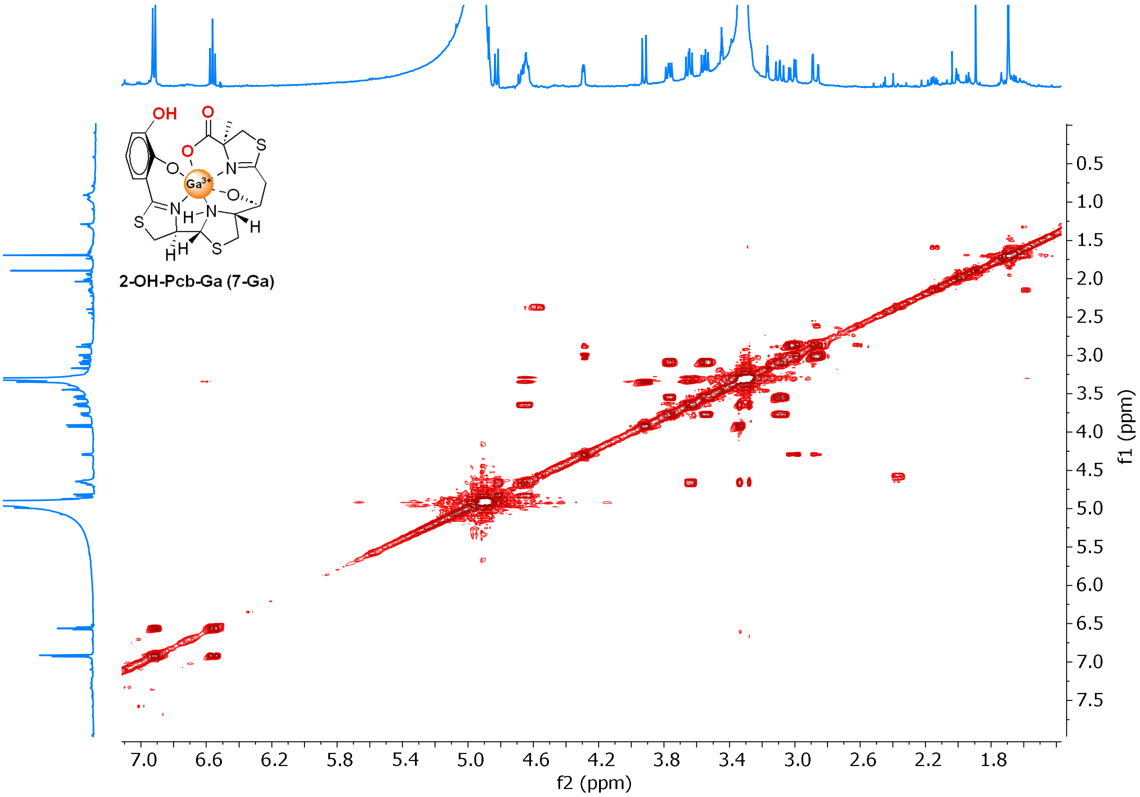

Figure S14: $^{1}H - ^{1}H$ COSY NMR spectrum (CD_3OD, 500 MHz) of 2-OH-Pcb-Ga (**7-Ga**) isolated from the H3 fraction, eluted with 1:1 MeCN:H_2O, from the mutant strain *V. anguillarum* RV22 Δ*vabF*.

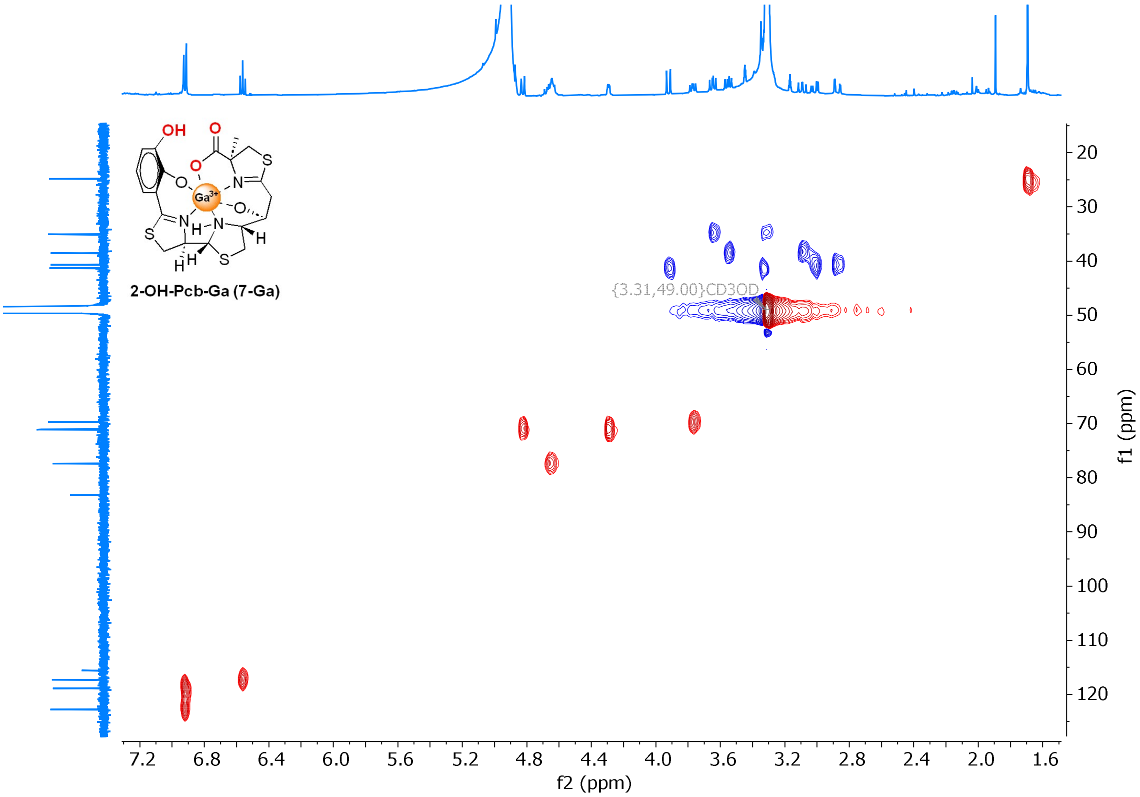

Figure S15: $^{1}H - ^{13}C$ HSQC NMR spectrum (MeOD, 500 MHz) of 2-OH-Pcb-Ga (**7-Ga**) isolated from the H3 fraction, eluted with 1:1 MeCN:H_2O, from the mutant strain *V. anguillarum* RV22 Δ*vabF*.

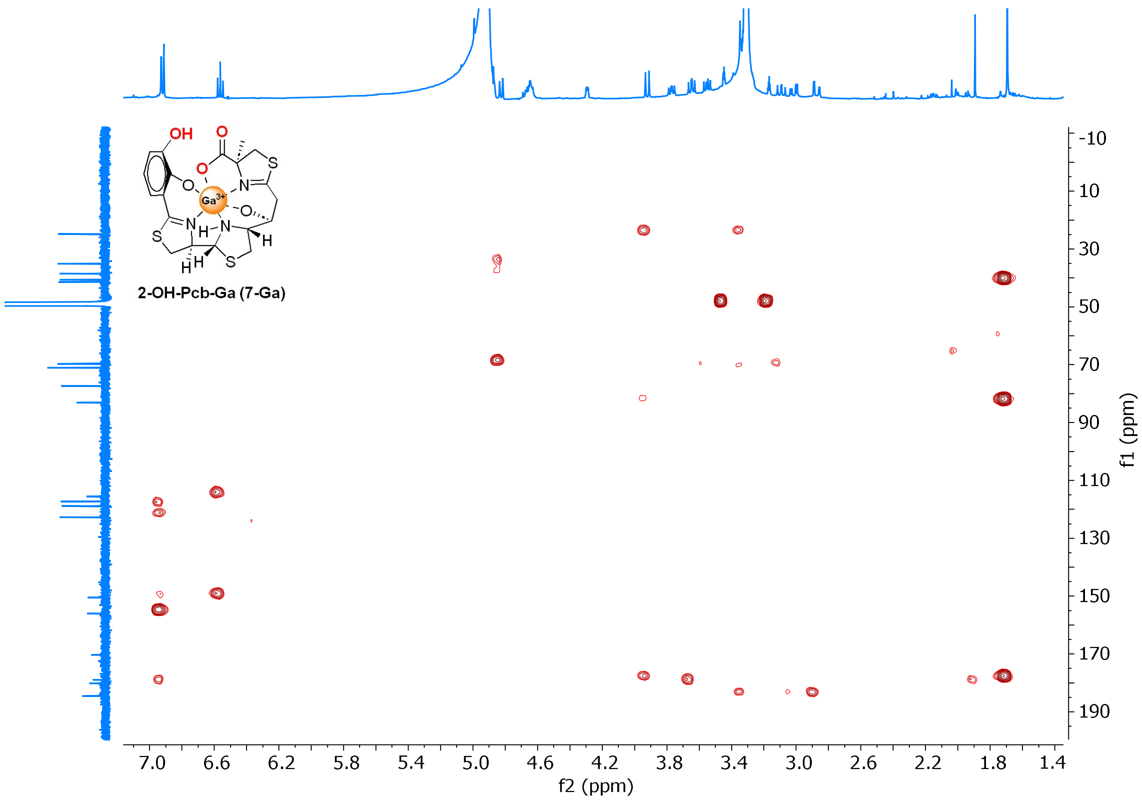

Figure S16: $^1H - ^{13}C$ HMBC NMR spectrum (MeOD, 500 MHz) of 2-OH-Pcb-Ga (**7-Ga**) isolated from the H3 fraction, eluted with 1:1 MeCN:H_2O, from the mutant strain *V. anguillarum* RV22 Δ*vabF*.

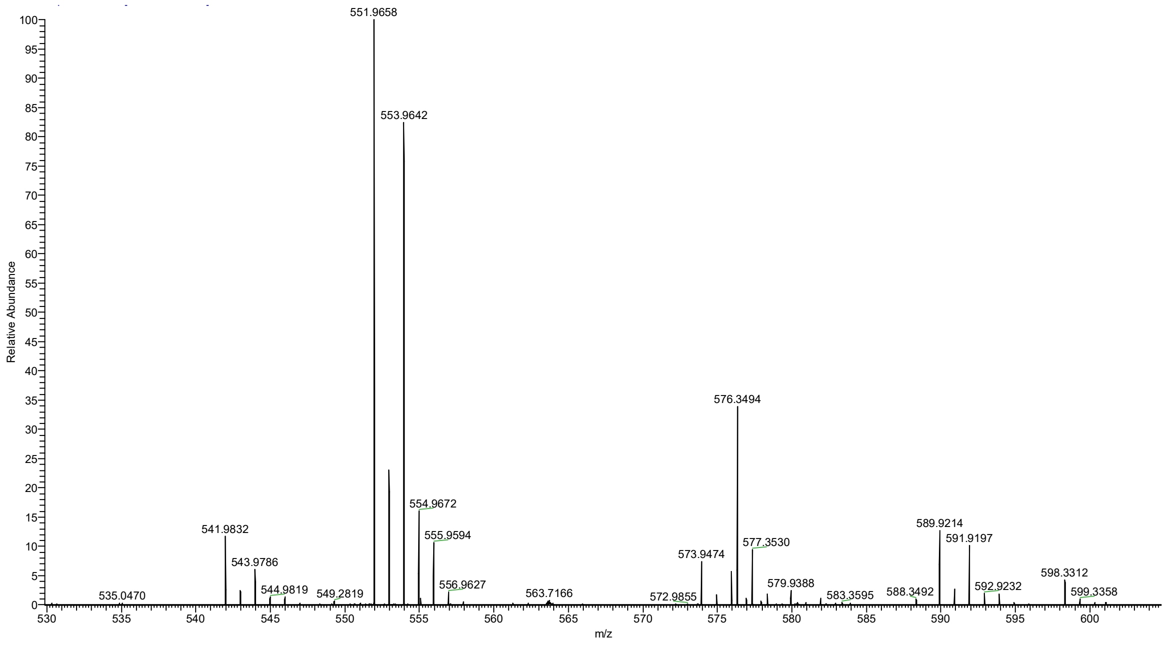

Figure S17: (+)-HRESIMS spectrum of 2-OH-PxbE-Ga (**8-Ga**) isolated from the H3 fraction, eluted with 1:1 MeCN:H_2O, from the mutant strain *V. anguillarum* RV22 Δ*vabF*.

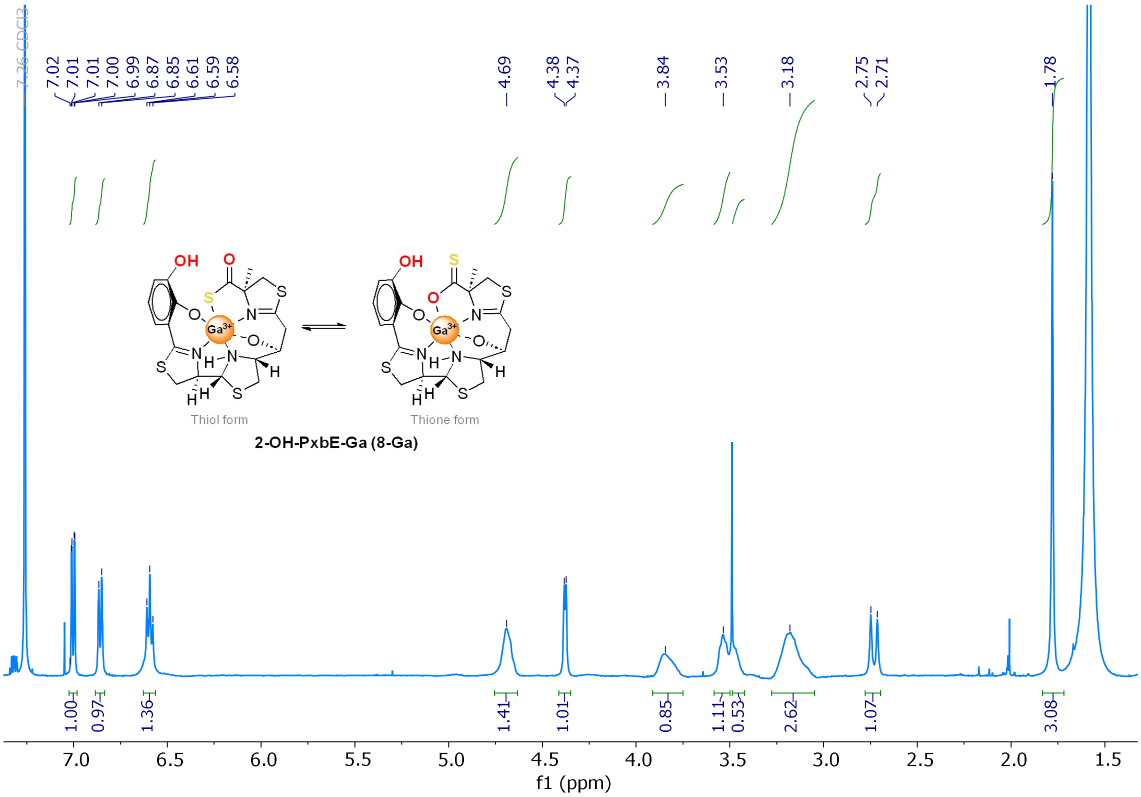

Figure S18: ^{1}H NMR spectrum ($CDCl_3$, 500 MHz) of 2-OH-PxbE-Ga (**8-Ga**) isolated from the H3 fraction, eluted with 1:1 MeCN:H_2O, from the mutant strain *V. anguillarum* RV22 Δ*vabF*.

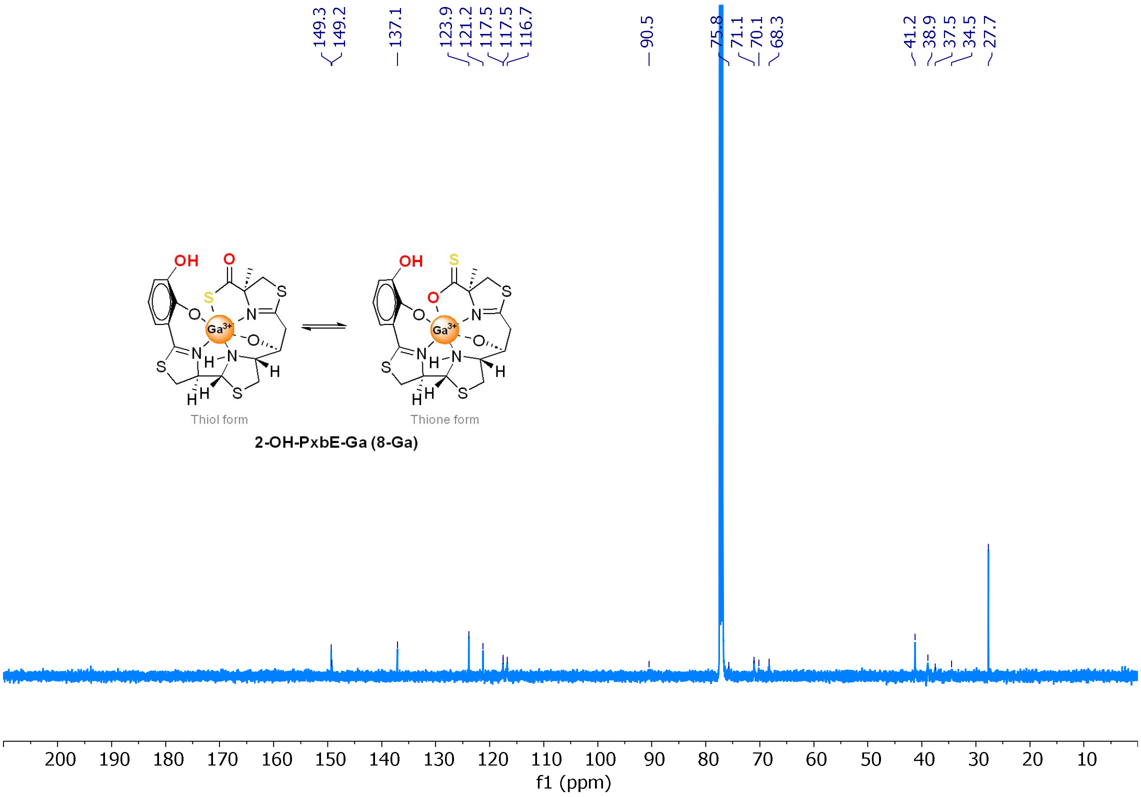

Figure S19: ^{13}C NMR spectrum ($CDCl_3$, 125 MHz) of 2-OH-PxbE-Ga (**8-Ga**) isolated from the H3 fraction, eluted with 1:1 MeCN:H_2O, from the mutant strain *V. anguillarum* RV22 Δ*vabF*.

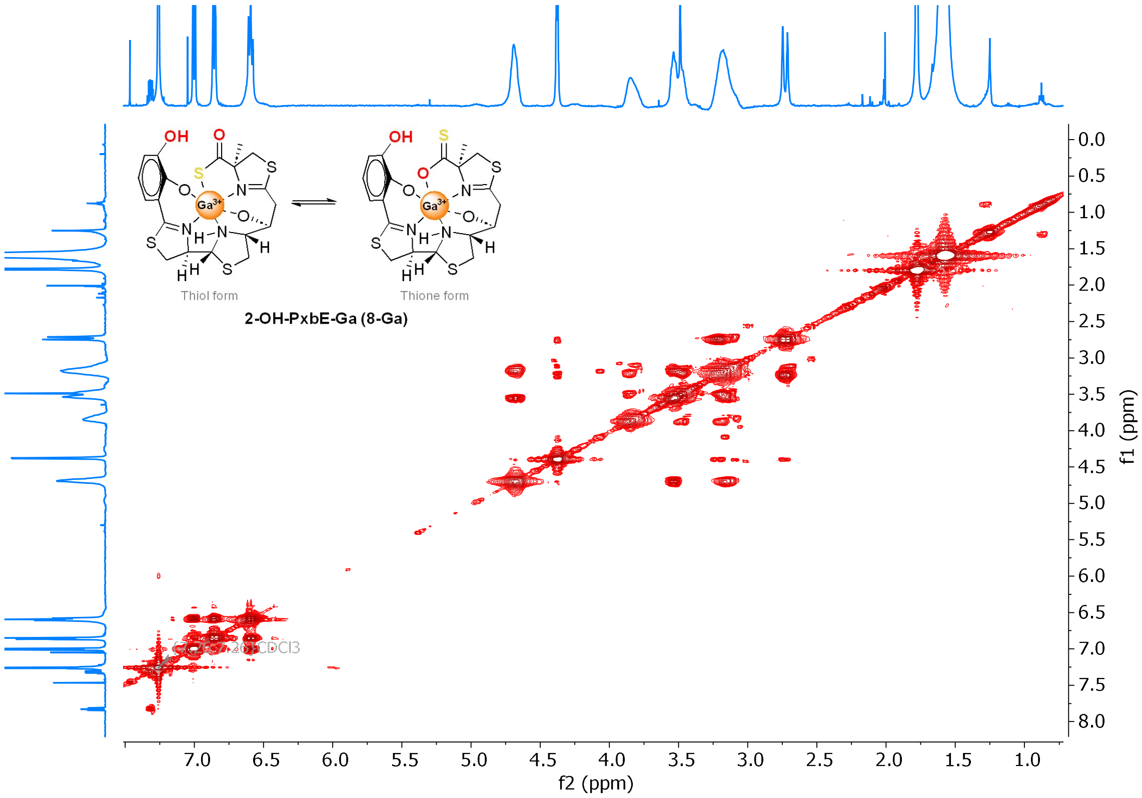

Figure S20: ^{1}H – ^{1}H COSY NMR spectrum ($CDCl_3$, 500 MHz) of 2-OH-PxbE-Ga (**8-Ga**) isolated from the H3 fraction, eluted with 1:1 MeCN:H_2O, from the mutant strain *V. anguillarum* RV22 Δ*vabF*.

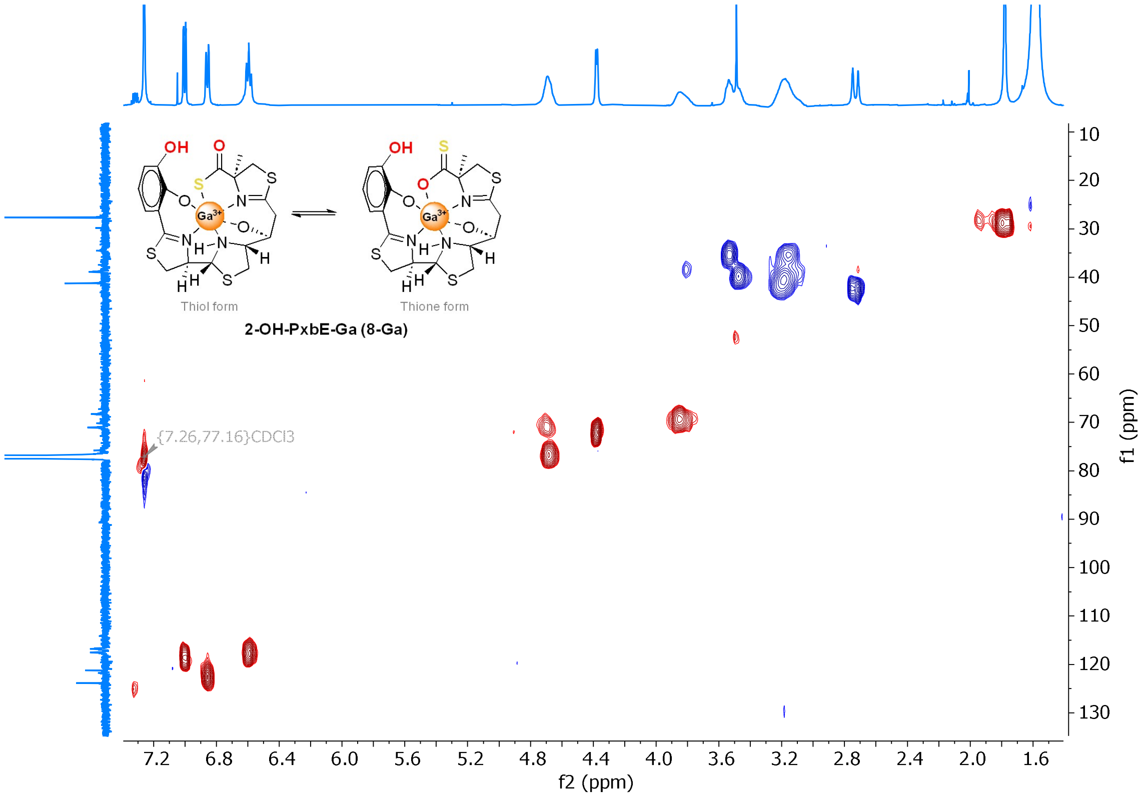

Figure S21: ^{1}H - ^{13}C HSQC NMR spectrum ($CDCl_3$, 500 MHz) of 2-OH-PxbE-Ga (**8-Ga**) isolated from the H3 fraction, eluted with 1:1 MeCN:H_2O, from the mutant strain *V. anguillarum* RV22 Δ*vabF*.

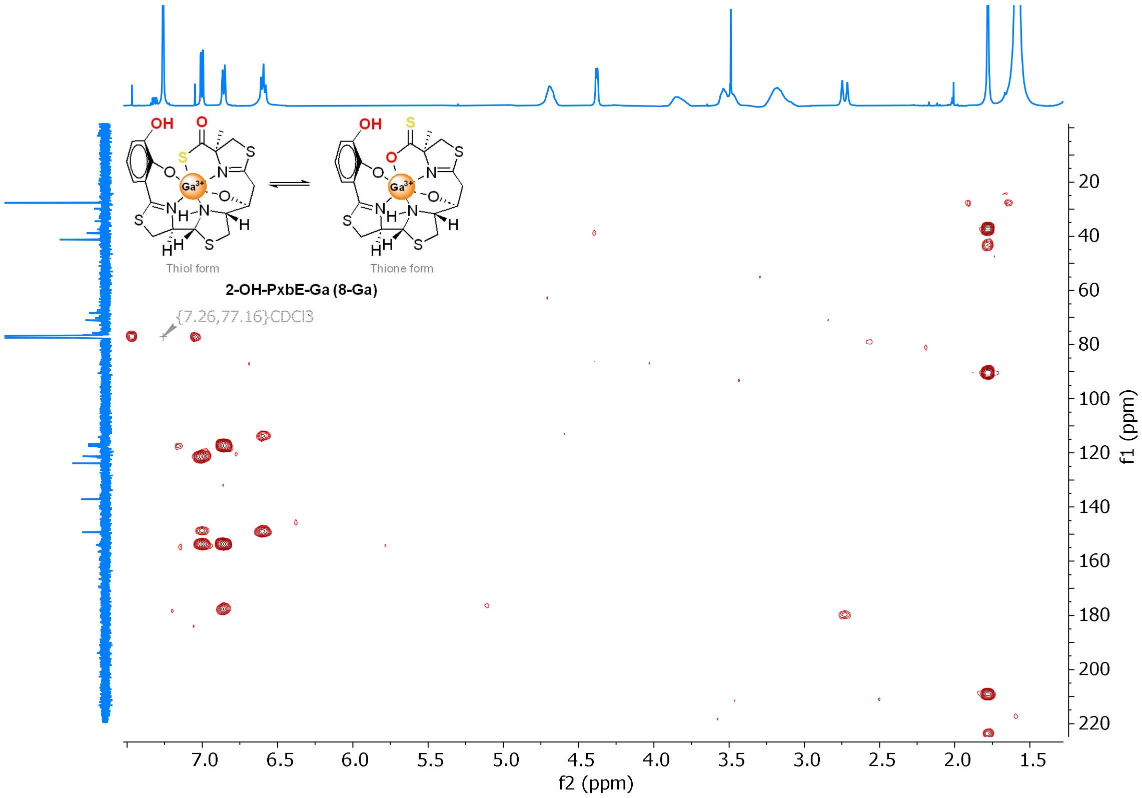

Figure S22: $^{1}H - ^{13}C$ HMBC NMR spectrum ($CDCl_3$, 500 MHz) of 2-OH-PxbE-Ga (**8-Ga**) isolated from the H3 fraction, eluted with 1:1 MeCN:H_2O, from the mutant strain *V. anguillarum* RV22 Δ*vabF*.

Table S5: ^{1}H and ^{13}C NMR data (500/125 MHz) assignments of Pcb-Ga (**3-Ga**, CD_3OD), and 2-OH-Pcb-Ga (CD_3OD).

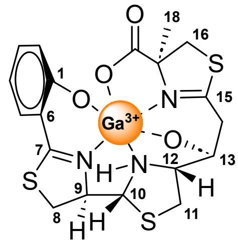

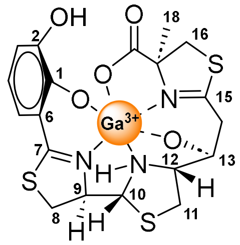

	**Pcb-Ga (1-Ga)**		**2-OH-Pcb-Ga (7-Ga)**	
	δ_H (mult., *J*)	δ_C, mult.	δ_H (mult., *J*)	δ_C, mult.
1	-	167.3, C	-	156.1, C
2	6.83 (dd, 8.5, 1.2)	123.7, C	-	150.5, C
3	7.36 (ddd, 8.7, 7.1, 1.8)	137.0, CH	6.92 (dd, 7.9, 1.8)	118.9, CH
4	6.70 (ddd, 8.1, 7.1, 1.2)	117.7, CH	6.56 (t, 7.9)	117.3, CH
5	7.42 (dd, 8.0, 1.8)	133.0, CH	6.93 (dd, 7.9, 0.8)	122.8, CH
6	-	116.7, C	-	115.6, C
7	-	180.0, C	-	180.2, C
8	3.3 (ov); 3.65 (dd, 10.9, 7.9)	35.0, CH_2	3.33 (ov); 3.65 (dd, 10.9, 7.8)	35.1, CH_2
9	4.65 (ddd, 13.0, 10.0, 8.2)	77.4, CH	4.66 (ddd, 13.0, 9.9, 7.9)	77.4, CH
10	4.83 (d, 9.9)	71.0, CH	4.82 (d, 9.9)	71.0, CH
10-NH	-	-	-	-
11	3.08 (dd, 12.3, 10.9), 3.55 (dd, 12.5, 7.0)	38.6, CH_2	3.09 (dd, 12.3, 10.9); 3.55 (dd 12.5, 7.0)	38.5, CH_2
12	3.76 (dd, 10.6, 7.2)	69.8, CH	3.77 (dd, 10.8, 7.1)	69.7, CH
13	4.27 (d, 4.0)	71.1, CH	4.30 (d, 5.3, 1.6)	71.1, CH
14	2.85 (dd, 16.8, 2.0); 3.03 (dd, 16.9, 5.0)	40.6, CH_2	2.87 (dd, 16.9, 2.1); 3.01 (dd 16.8, 5.0)	40.7, CH_2
15	-	184.4, C	-	184.5, C
16	3.34 (ov); 3.90 (d, 11.3)	41.3, CH_2	3.34 (ov); 3.92 (d, 11.4)	41.4, CH_2
17	-	83.1, C	-	83.2, C
18	1.69, s	24.4, CH_3	1.69 (s)	24.8, CH_3
19	-	178.9, C	-	179.0, C

ov = overlapped resonances

Table S6: ^{1}H and ^{13}C NMR data (500/125 MHz) assignments of PxbE-Ga (**6-Ga**, CD_3SOCD_3), and 2-OH-PxbE-Ga (**8-Ga**, $CDCl_3$).

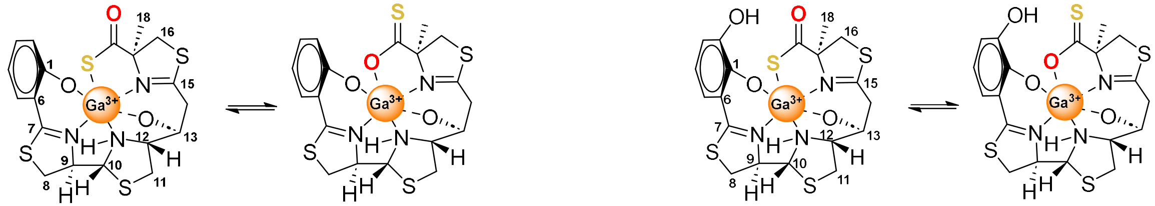

Thiol form — Thione form — Thiol form — Thione form

	**PxbE-Ga (6-Ga)**				**2-OH-PxbE-Ga (8-Ga)**			
	δ_H (mult., *J*)	δ_C, mult.	δ_H (mult., *J*)	δ_C, mult.	δ_H (mult., *J*)	δ_C, mult.	δ_H (mult., *J*)	δ_C, mult.
1	-	166.3, C	-	166.3, C	-	153.6, C	-	153.6, C
2	6.67 (m)	122.9, CH	6.67 (m)	122.7, CH	-	148.5, C	-	148.5, C
3	7.32 (m)	135.3, CH	7.33 (m)	135.4, CH	7.00 (dd, 7.6, 1.5)	117.6, CH	7.00 (dd, 7.6, 1.5)	117.6, CH
4	6.61 (m)	115.3, CH	6.63 (m)	115.3, CH	6.59 (t, 7.6)	116.7, CH	6.59 (t, 7.6)	116.7, CH
5	7.34 (m)	131.6, CH	7.27 (m)	131.6, CH	6.86 (d, 8.2)	121.8, CH	6.86 (d, 8.2)	121.8, CH
6	-	115.8, C	-	115.0, C	-	117.1, C	-	117.1, C
7	-	176.4, C	-	174.8, C	-	177.5, C	-	177.5, C
8	3.34 (ov), 3.59 (ov)	33.0, CH_2	3.34 (ov), 3.53 (ov)	33.4, CH_2	3.15 (ov); 3.54 (ov)	34.5, CH_2	3.15 (ov); 3.54 (ov)	34.5, CH_2
9	4.62 (m)	75.6, CH	4.61 (m)	75.6, CH	4.69 (ov)	75.8, CH	4.68 (ov)	75., CH
10	4.86 (d, 9.4)	68.8, CH	4.89 (d, 9.4)	68.7, CH	4.70 (ov)	70.0, CH	4.70 (ov)	70.0, CH
10-NH	6.37 (brs)*		6.64 (brs)*	-				
11	2.99 (m), 3.50 (ov)	37.4, CH_2	2.99 (m), 3.50 (ov)	37.4, CH_2	3.18 (ov); 3.47 (ov)	38.8, CH_2	3.18 (ov); 3.46 (ov)	38.8, CH_2
12	3.70 (m)**	68.3, CH	3.62 (m)**	68.6, CH	3.84 (m)	68.4, CH	3.84 (m)	68.4, CH
13	4.06 (brs)	70.1, CH	4.10 (brs)	70.2, CH	4.37 (d, 4.9)	71.1, CH	4.37 (d, 4.9)	71.1, CH
14	2.70 (d, 14.7), 2.92 (m)	40.3, CH_2	2.76 (d, 14.7), 2.92 (m)	40.3, CH_2	2.71 (d, 17.4); 3.22 (ov)	41.2, CH_2	2.71 (d, 17.4); 3.22 (ov)	41.2, CH_2
15	-	180.2, C	-	181.9, C	-	179.9, C	-	179.1, C
16	3.16, (d 11.5), 3.50 (ov)	38.2, CH_2	3.34 (ov), 4.01 (d, 11.1)	42.2, CH_2	3.10 (ov); 3.80 (ov);	37.7, CH_2	2.74 (s); 3.20 (ov)	43.0, CH_2
17	-	88.8	-	88.8	-	90.3, C	-	90.3, C
18	1.51 (s)	24.4, CH_3	1.61 (s)	25.4, CH_3	1.78, s	27.6, CH_3	1.78, s	27.6, CH_3
19	-	208.7, C	-	222.2, C	-	209.0, C		223.5, C

ov = overlapped resonances
*,** = interchangeable signals

5. MS/MS fragmentation pattern of Pcb/Pxb catechol analogues

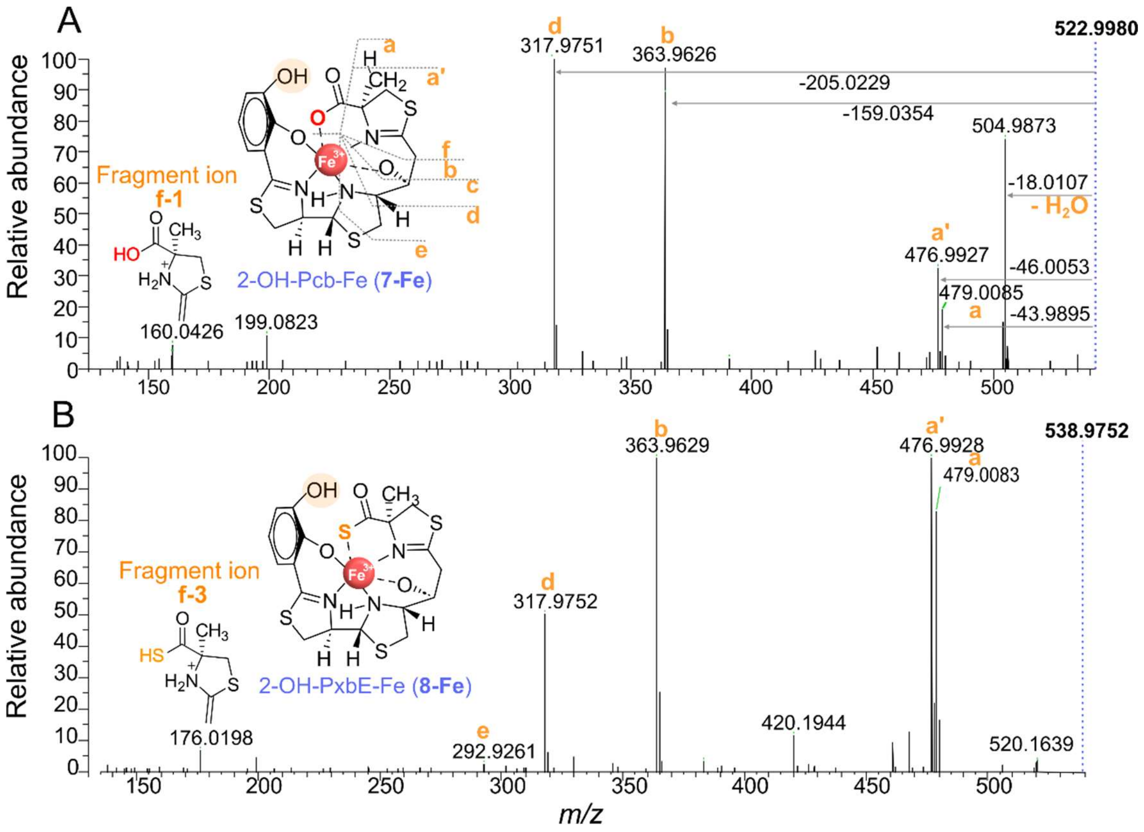

Figure S23: MS/MS fragmentation pattern of: **A)** 2-OH-Pcb-Fe (**7-Fe**) and **B)** 2-OH-PxbE-Fe (**8-Fe**)

6. Mutant profiling extracted ion chromatograms

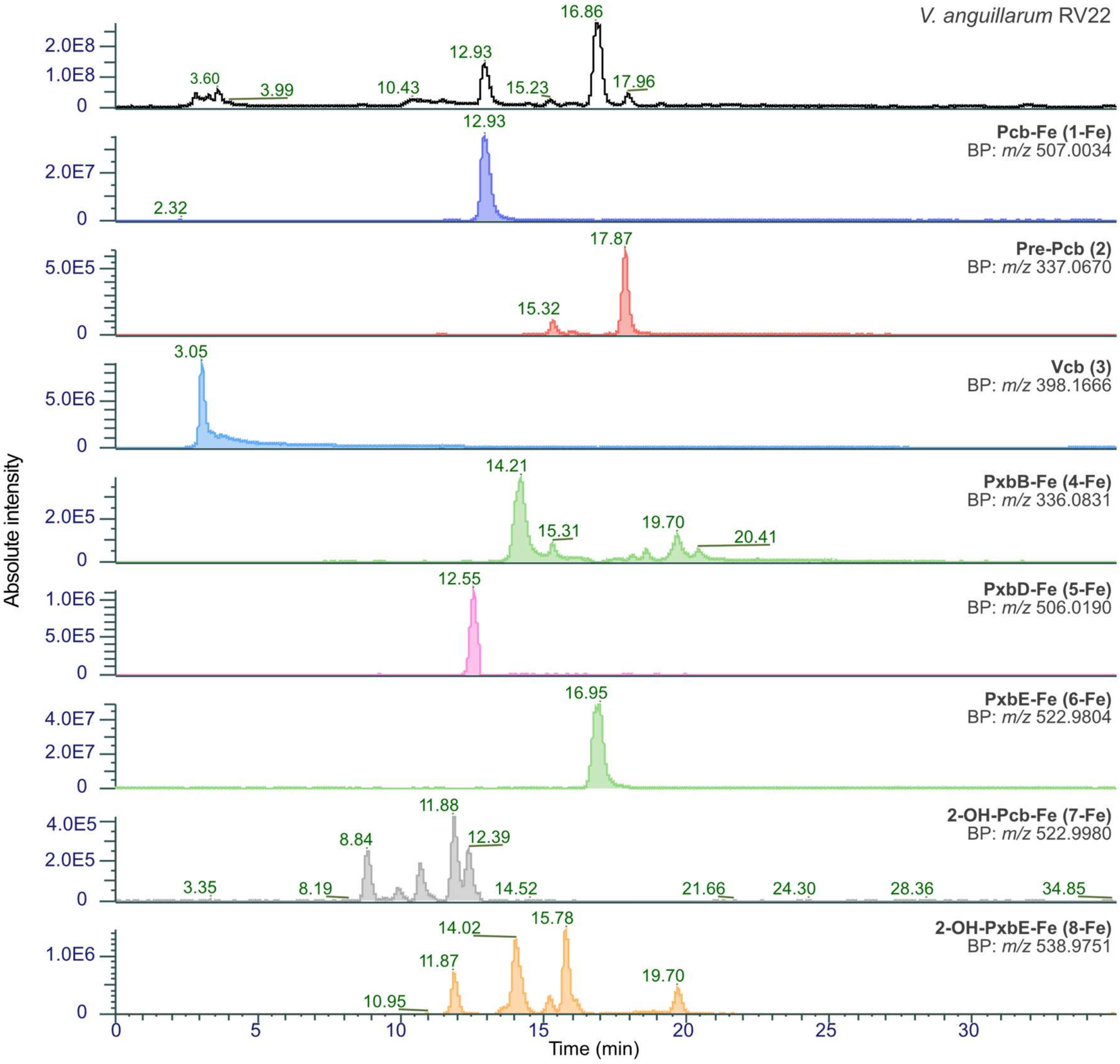

Figure S24: Total Ion Chromatogram (TIC) of the H3 fraction chelated with iron from the wild-type strain *V. anguillarum* RV22 on the top, followed by the extracted ion chromatograms of the different siderophores reported within this work (Pcb-Fe (**1-Fe**), Pre-Pcb (**2**), Vcb (**3**), PxbB-Fe (**4-Fe**), PxbD-Fe (**5-Fe**), PxbE-Fe (**6-Fe**), 2-OH-Pcb-Fe (**7-Fe**), 2-OH-PxbE-Fe (**8-Fe**)).

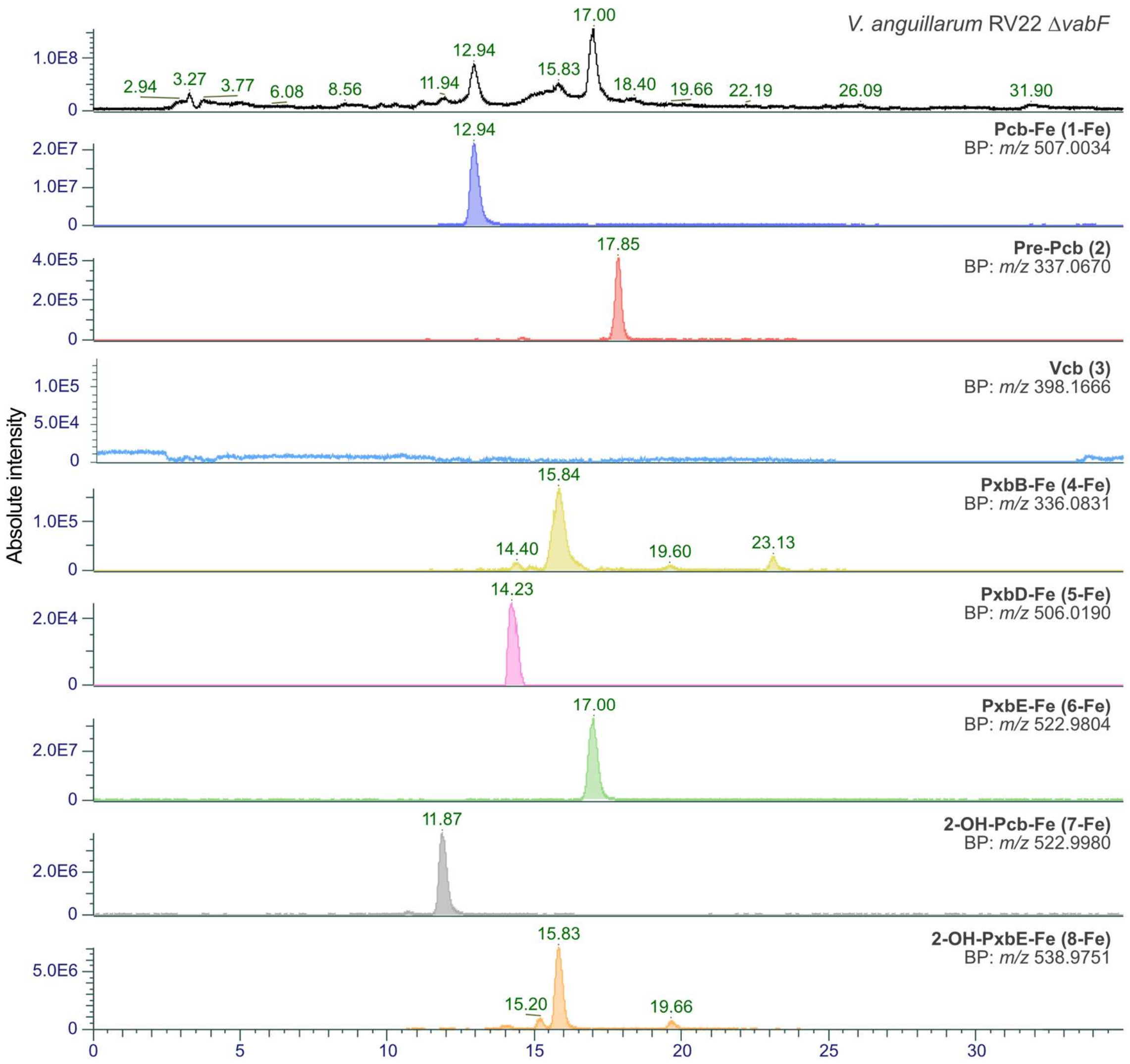

Figure S25: Total Ion Chromatogram (TIC) of the H3 fraction chelated with iron from the mutant strain *V. anguillarum* RV22 Δ*vabF* on the top, followed by the extracted ion chromatograms of the different siderophores reported within this work (Pcb-Fe (**1-Fe**)**,** Pre-Pcb (**2**), Vcb (**3**), PxbB-Fe (**4-Fe**), PxbD-Fe (**5-Fe**), PxbE-Fe (**6-Fe**), 2-OH-Pcb-Fe (**7-Fe**), 2-OH-PxbE-Fe (**8-Fe**)).

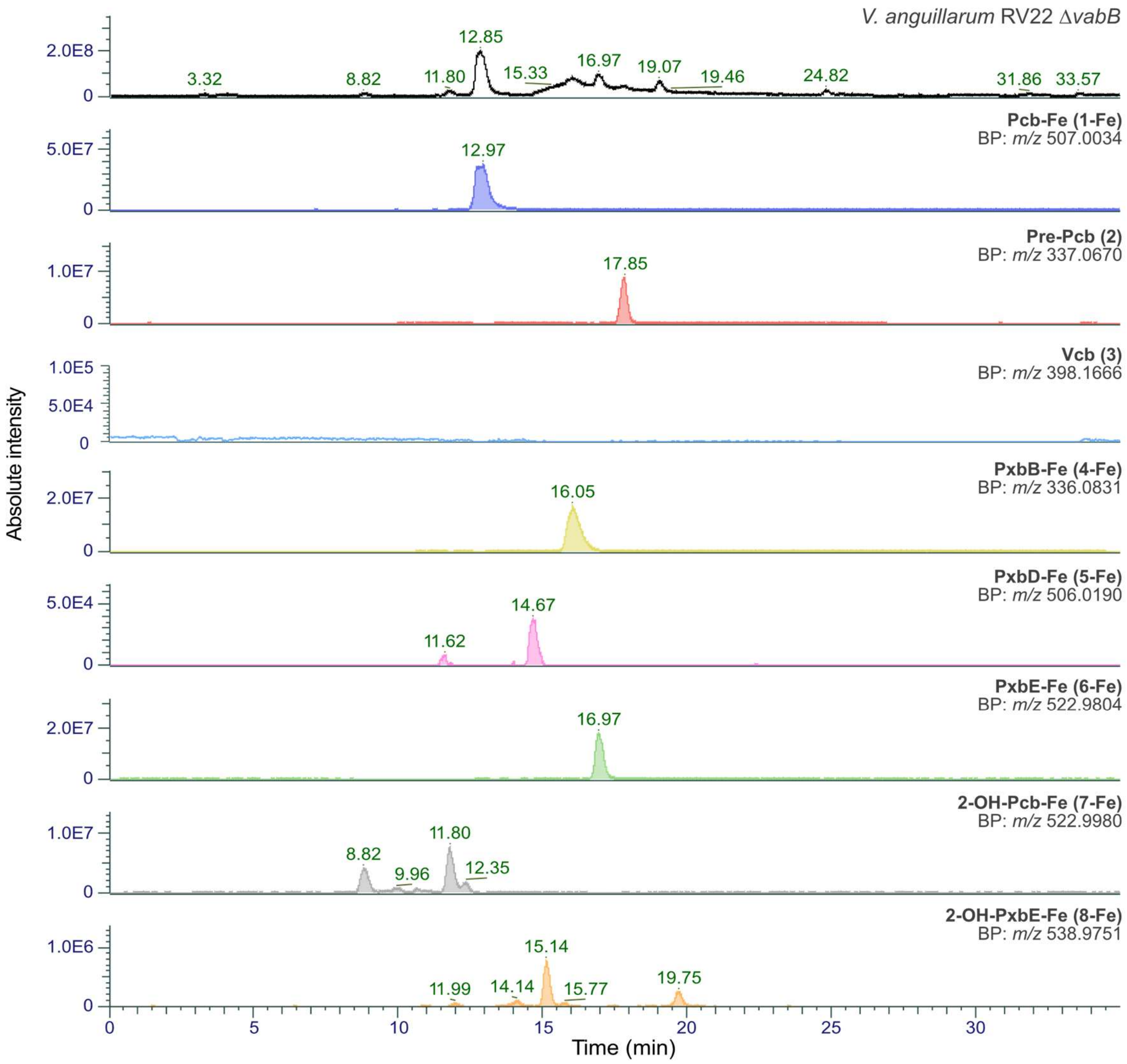

Figure S26: Total Ion Chromatogram (TIC) of the H3 fraction chelated with iron from the mutant strain *V. anguillarum* RV22 Δ*vabB* on the top, followed by the extracted ion chromatograms of the different siderophores reported within this work (Pcb-Fe (**1-Fe**), Pre-Pcb (**2**), Vcb (**3**), PxbB-Fe (**4-Fe**), PxbD-Fe (**5-Fe**), PxbE-Fe (**6-Fe**), 2-OH-Pcb-Fe (**7-Fe**), 2-OH-PxbE-Fe (**8-Fe**)).

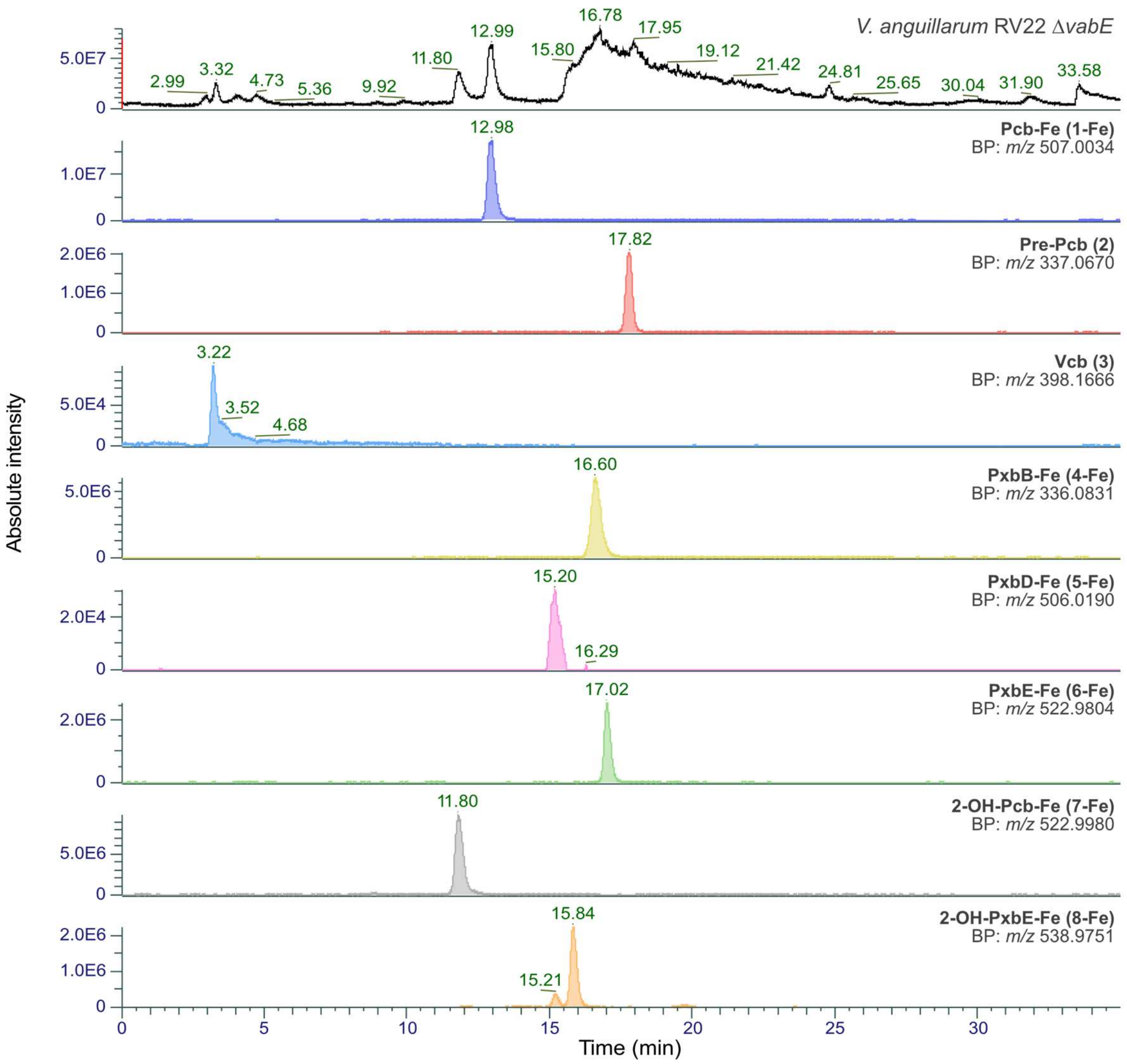

Figure S27: Total Ion Chromatogram (TIC) of the H3 fraction chelated with iron from the mutant strain *V. anguillarum* RV22 Δ*vabE* on the top, followed by extracted ion chromatograms of the different siderophores reported within this work (Pcb-Fe (**1-Fe),** Pre-Pcb (**2**), Vcb (**3**), PxbB-Fe (**4-Fe**), PxbD-Fe (**5-Fe**), PxbE-Fe (**6-Fe**), 2-OH-Pcb-Fe (**7-Fe**), 2-OH-PxbE-Fe (**8-Fe**)).

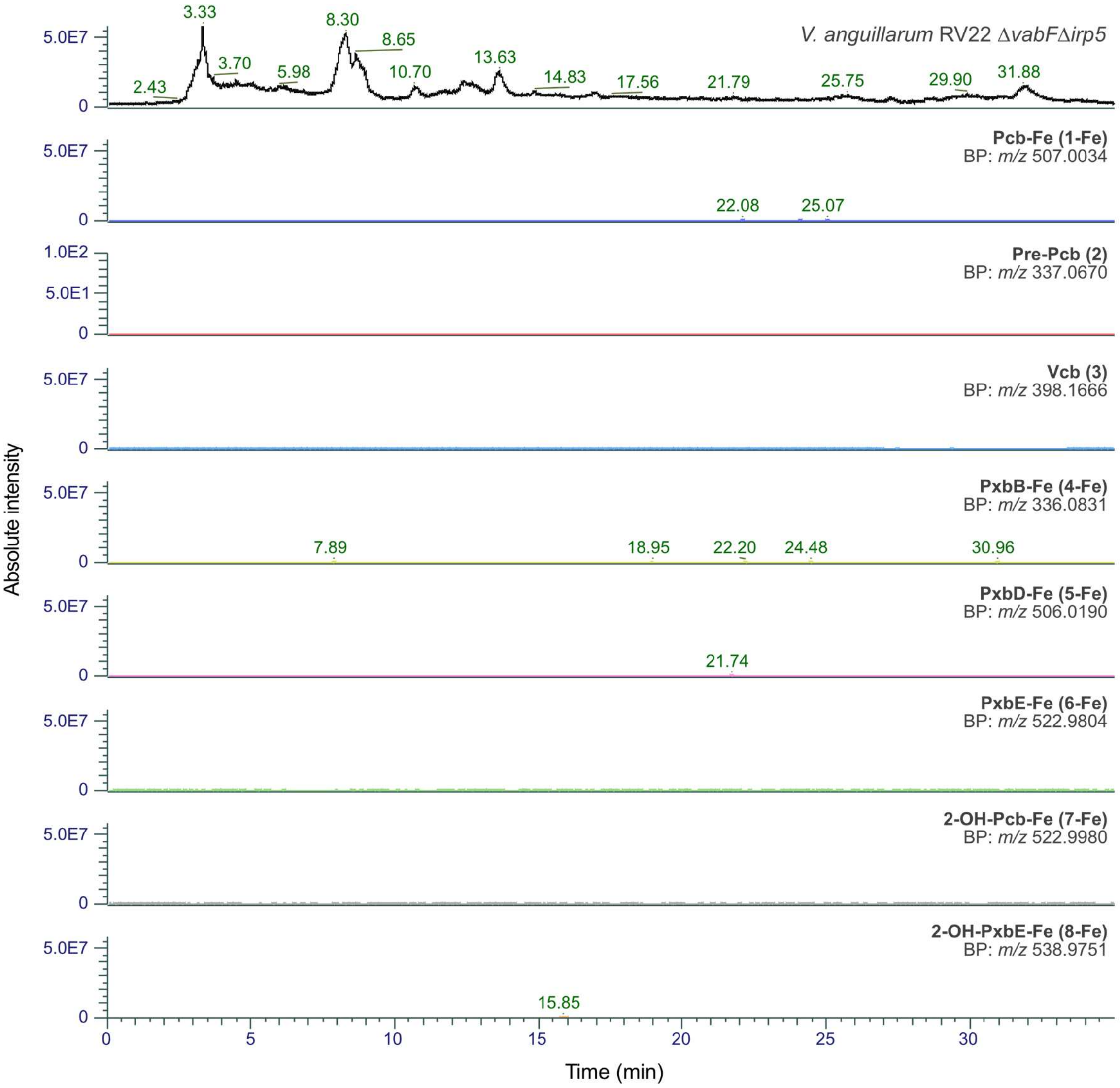

Figure S28: Total Ion Chromatogram (TIC) of the H3 fraction chelated with iron from the mutant strain *V. anguillarum* RV22 Δ*vabF*Δ*irp5* on the top, followed by the extracted ion chromatograms of the different siderophores reported within this work (Pcb-Fe (**1-Fe**), Pre-Pcb (**2**), Vcb (**3**), PxbB-Fe (**4-Fe**), PxbD-Fe (**5-Fe**), PxbE-Fe (**6-Fe**), 2-OH-Pcb-Fe (**7-Fe**), 2-OH-PxbE-Fe (**8-Fe**)).

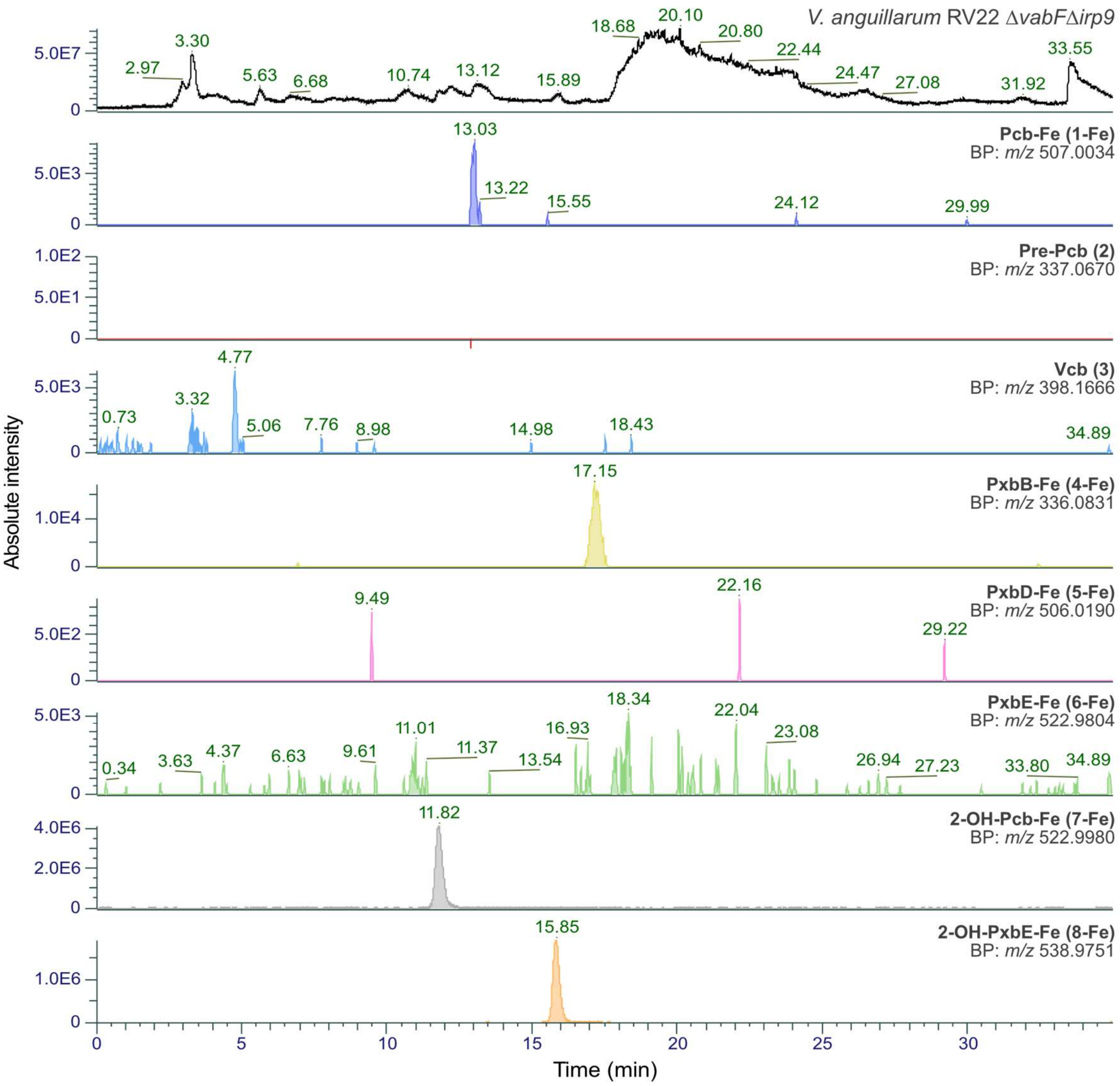

Figure S29: Total Ion Chromatogram (TIC) of the H3 fraction chelated with iron from the mutant strain *V. anguillarum* RV22 Δ*vabF*Δ*irp9* on the top, followed by the extracted ion chromatograms of the different siderophores reported within this work (Pcb-Fe (**1-Fe**)**,** Pre-Pcb (**2**), Vcb (**3**), PxbB-Fe (**4-Fe**), PxbD-Fe (**5-Fe**), PxbE-Fe (**6-Fe**), 2-OH-Pcb-Fe (**7-Fe**), 2-OH-PxbE-Fe (**8-Fe**)).

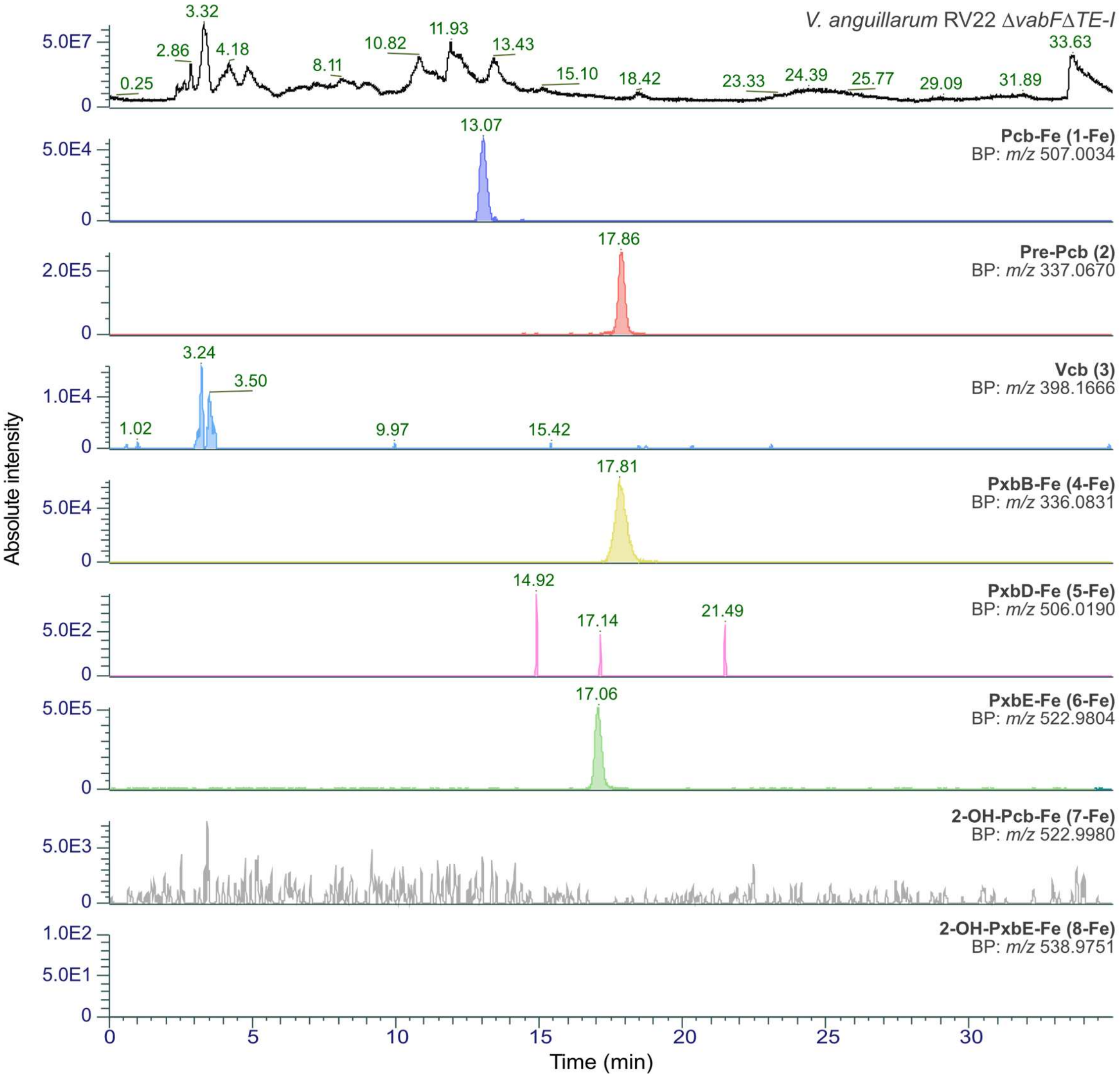

Figure S30: Total Ion Chromatogram (TIC) of the H3 fraction chelated with iron from the mutant strain *V. anguillarum* RV22 Δ*vabF*Δ*TE-I* on the top, followed by the extracted ion chromatograms of the different siderophores reported within this work (Pcb-Fe (**1-Fe**)**,** Pre-Pcb (**2**), Vcb (**3**), PxbB-Fe (**4-Fe**), PxbD-Fe (**5-Fe**), PxbE-Fe (**6-Fe**), 2-OH-Pcb-Fe (**7-Fe**), 2-OH-PxbE-Fe (**8-Fe**)).

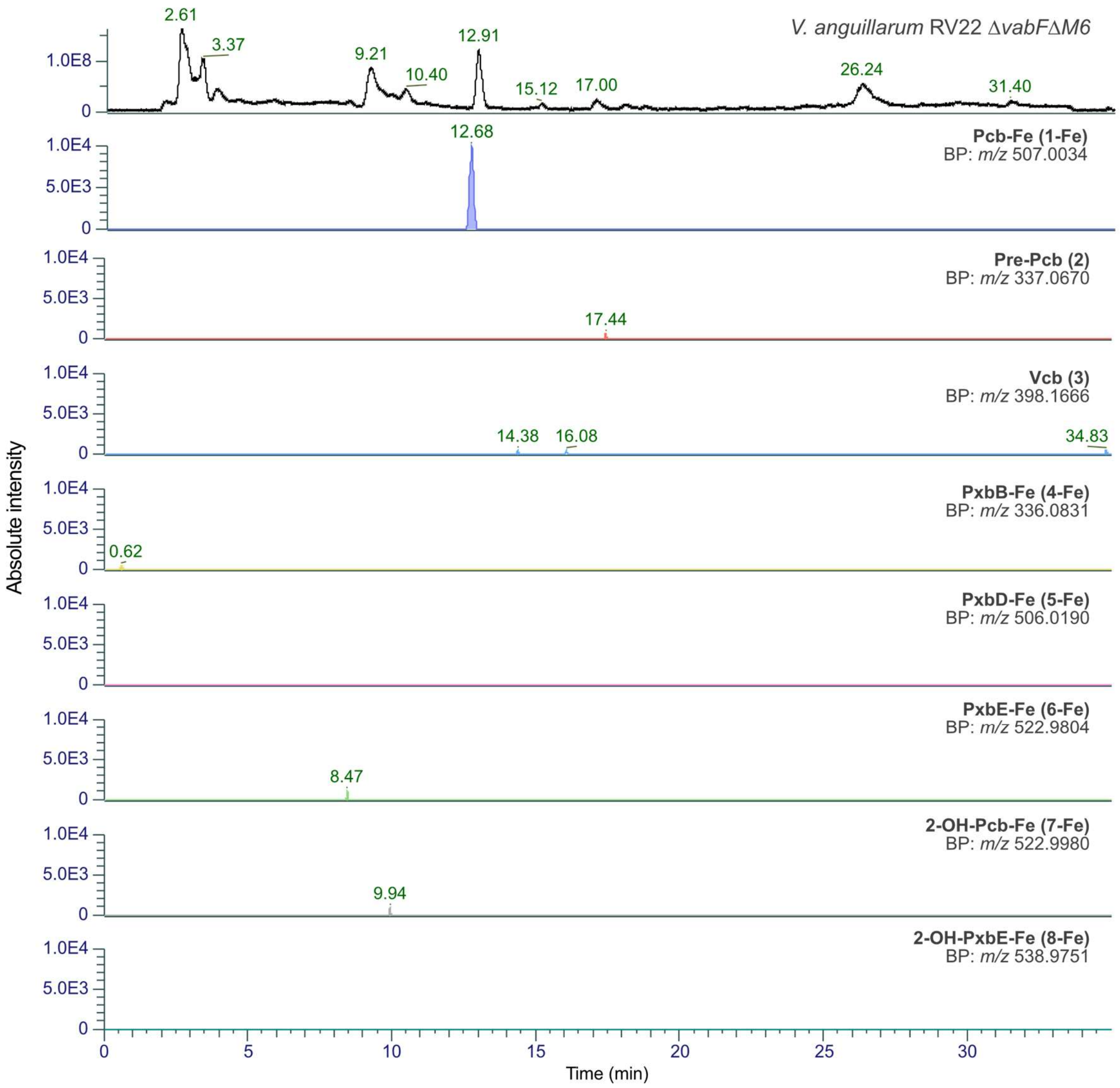

Figure S31: Total Ion Chromatogram (TIC) of the H3 fraction chelated with iron from the mutant strain *V. anguillarum* RV22 Δ*vabF*Δ*M6* on the top, followed by the extracted ion chromatograms of the different siderophores reported within this work (Pcb-Fe (**1-Fe**), Pre-Pcb (**2**), Vcb (**3**), PxbB-Fe (**4-Fe**), PxbD-Fe (**5-Fe**), PxbE-Fe (**6-Fe**), 2-OH-Pcb-Fe (**7-Fe**), 2-OH-PxbE-Fe (**8-Fe**)).

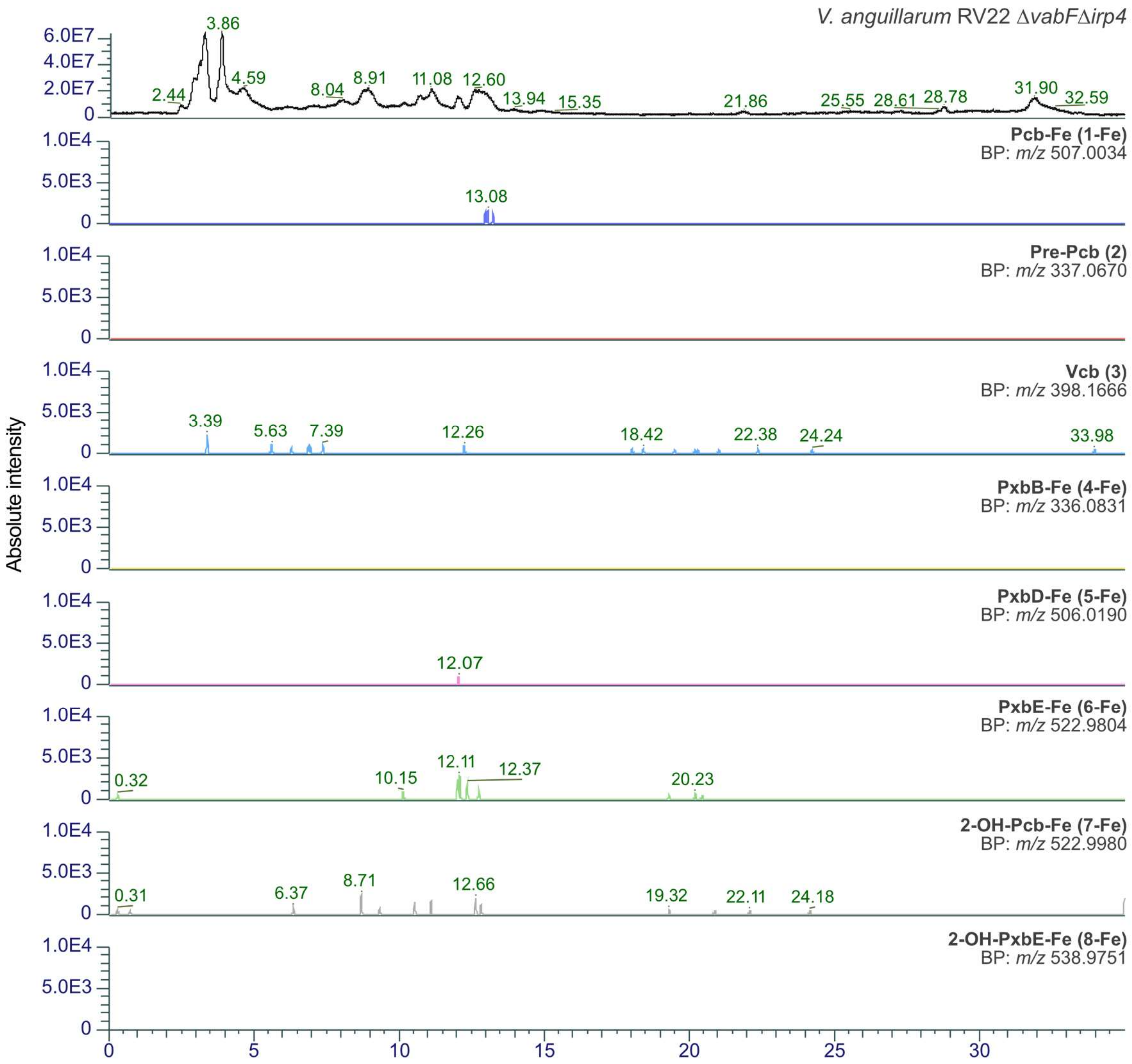

Figure S32: Total Ion Chromatogram (TIC) of the H3 fraction chelated with iron from the mutant strain *V. anguillarum* RV22 Δ*vabF*Δ*irp4* on the top, followed by the extracted ion chromatograms of the different siderophores reported within this work (Pcb-Fe (**1-Fe**)**,** Pre-Pcb (**2**), Vcb (**3**), PxbB-Fe (**4-Fe**), PxbD-Fe (**5-Fe**), PxbE-Fe (**6-Fe**), 2-OH-Pcb-Fe (**7-Fe**), 2-OH-PxbE-Fe (**8-Fe**)).

7. Precursor-directed biosynthesis (PDB) analyses

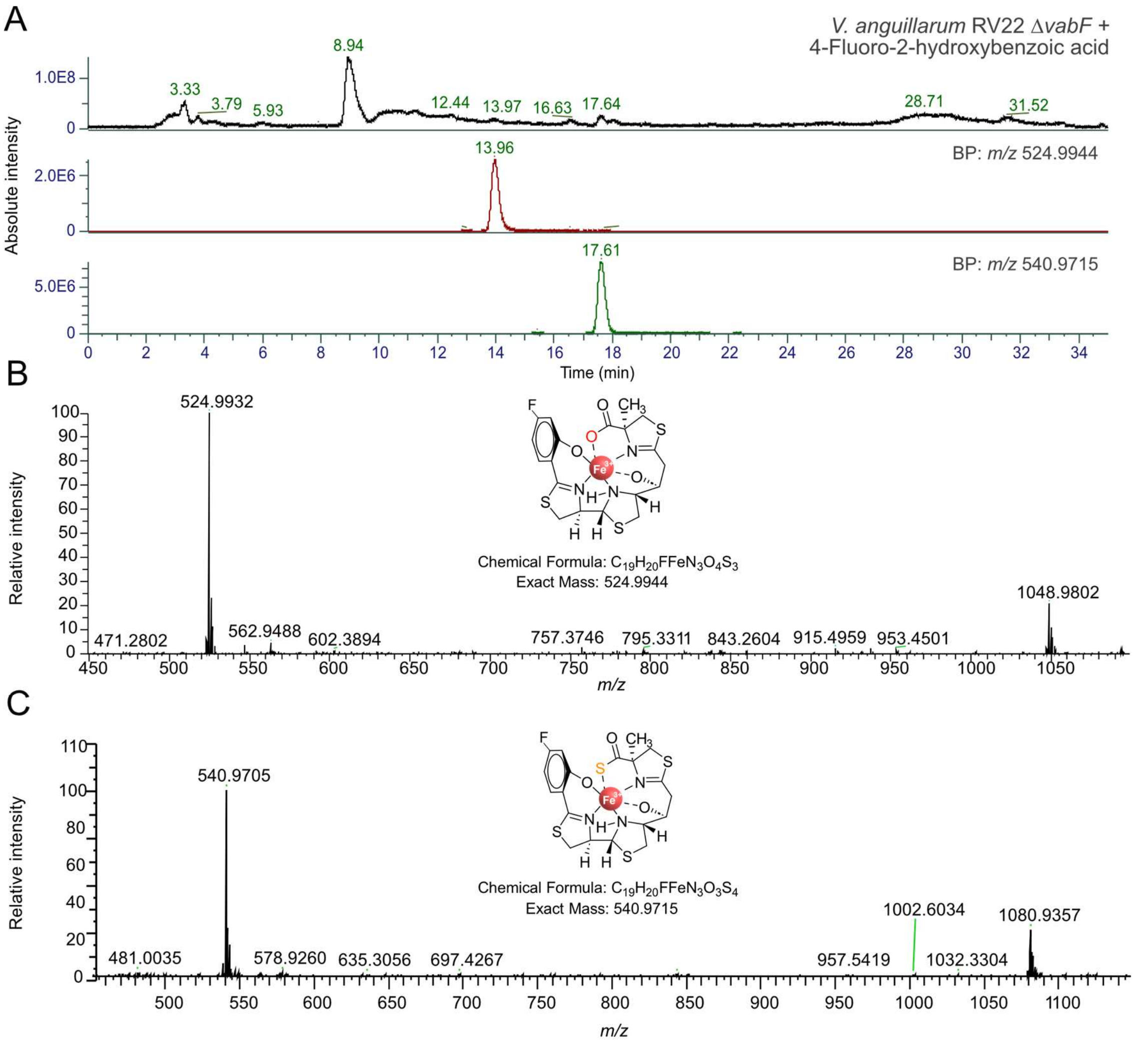

Figure S33: Precursor directed biosynthesis (PDB) analysis by addition of 4-Fluoro-2-hydroxybenzoic acid (4-F-2-HBA) in the culture of *V. anguillarum* RV22 Δ*vabF*. **A**) Total ion chromatogram of *V. anguillarum* RV22 ΔvabF H3 fraction followed by the extracted ion chromatogram of the expected new analogues with Pcb and PxbE bearing the introduced in the structure. **B**) (+)-HRESIMS of the peak eluting at 13.96 min corresponding to Pcb bearing 4-F-2-HBA in its structure. **C**) (+)-HRESIMS of the peak eluting at 17.61 min corresponding to PxbE bearing 4-F-2-HBA in its structure.

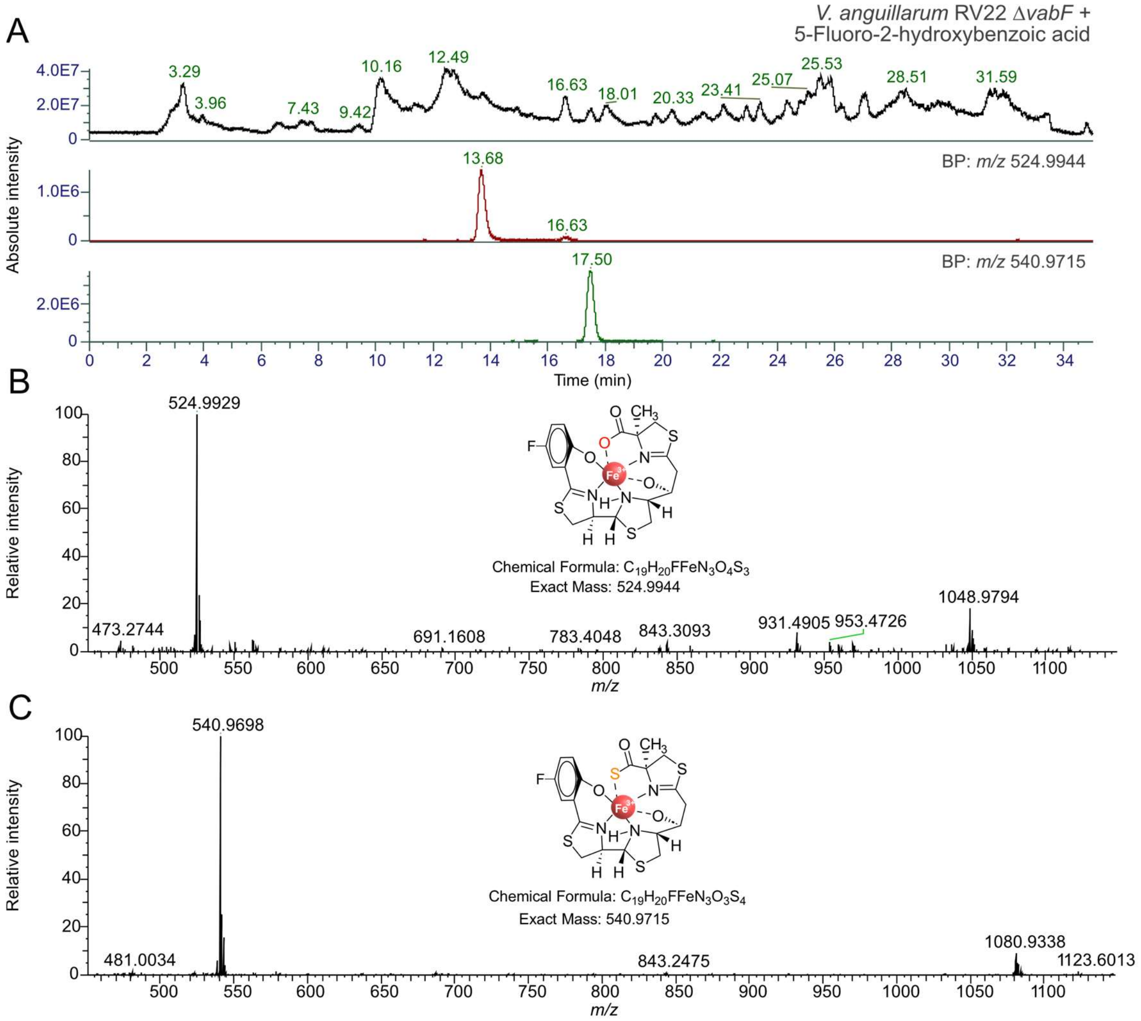

Figure S34: Precursor directed biosynthesis (PDB) analysis by addition of 5-Fluoro-2-hydroxybenzoic acid (5-F-2-HBA) to the culture of *V. anguillarum* RV22 Δ*vabF*. A) Total ion chromatogram of *V. anguillarum* RV22 ΔvabF H3 fraction followed by the extracted ion chromatogram of the expected new analogues with Pcb and PxbE bearing the introduced in the structure. **B**) (+)-HRESIMS of the peak eluting at 13.68 min corresponding to Pcb bearing 5-F-2-HBA in its structure. **C**) (+)-HRESIMS of the peak eluting at 17.50 min corresponding to PxbE bearing 5-F-2-HBA in its structure.
